# Safety, pharmacokinetics, and immunogenicity of the combination of the broadly neutralizing anti-HIV-1 antibodies 3BNC117 and 10-1074 in healthy adults: A randomized, phase 1 study

**DOI:** 10.1371/journal.pone.0219142

**Published:** 2019-08-08

**Authors:** Yehuda Z. Cohen, Allison L. Butler, Katrina Millard, Maggi Witmer-Pack, Rebeka Levin, Cecilia Unson-O’Brien, Roshni Patel, Irina Shimeliovich, Julio C. C. Lorenzi, Jill Horowitz, Stephen R. Walsh, Shu Lin, Joshua A. Weiner, Anna Tse, Alicia Sato, Chelsey Bennett, Bryan Mayer, Kelly E. Seaton, Nicole L. Yates, Lindsey R. Baden, Allan C. deCamp, Margaret E. Ackerman, Michael S. Seaman, Georgia D. Tomaras, Michel C. Nussenzweig, Marina Caskey

**Affiliations:** 1 Laboratory of Molecular Immunology, The Rockefeller University, New York, New York, United States of America; 2 Division of Infectious Diseases, Brigham and Women’s Hospital, Boston, Massachusetts, United States of America; 3 Thayer School of Engineering, Dartmouth College, Hanover, New Hampshire, United States of America; 4 Center for Virology and Vaccine Research, Beth Israel Deaconess Medical Center, Boston, Massachusetts, United States of America; 5 Vaccine and Infectious Disease Division and Statistical Center for HIV/AIDS Research and Prevention, Fred Hutchinson Cancer Research Center, Seattle, Washington, United States of America; 6 Duke Human Vaccine Institute, Duke University Medical Center, Durham, North Carolina, United States of America; 7 Departments of Surgery, Immunology, Molecular Genetics and Microbiology, Duke University Medical Center, Durham, North Carolina, United States of America; 8 Howard Hughes Medical Institute, Chevy Chase, Maryland, United States of America; Rush University, UNITED STATES

## Abstract

**Background:**

Additional forms of pre-exposure prophylaxis are needed to prevent HIV-1 infection. 3BNC117 and 10–1074 are broadly neutralizing anti-HIV-1 antibodies that target non-overlapping epitopes on the HIV-1 envelope. We investigated the safety, tolerability, pharmacokinetics, and immunogenicity of the intravenous administration of the combination of 3BNC117 and 10–1074 in healthy adults.

**Methods:**

This randomized, double-blind, placebo-controlled, single center, phase 1 study enrolled healthy adults aged 18–65 years to receive one infusion of 3BNC117 immediately followed by 10–1074 at 10 mg/kg, three infusions of 3BNC117 followed by 10–1074 at 3 mg/kg or 10 mg/kg every 8 weeks, or placebo infusions. The primary outcomes were safety and pharmacokinetics. This trial is registered with ClinicalTrials.gov, number NCT02824536.

**Findings:**

Twenty-four participants were enrolled in a 3:1 ratio to receive the study products or placebo. The combination of 3BNC117 and 10–1074 was safe and generally well tolerated. There were no serious adverse events considered related to the infusions. The mean elimination half-lives of 3BNC117 and 10–1074 were 16.4 ± 4.6 days and 23.0 ± 5.4 days, respectively, similar to what was observed in previous studies in which each antibody was administered alone. Anti-drug antibody responses were rare and without evidence of related adverse events or impact on elimination kinetics.

**Interpretation:**

Single and repeated doses of the combination of 3BNC117 and 10–1074 were well tolerated in healthy adults. These data support the further development of the combination of 3BNC117 and 10–1074 as a long-acting injectable form of pre-exposure prophylaxis for the prevention of HIV-1 infection.

## Introduction

Daily oral pre-exposure prophylaxis (PrEP) can significantly reduce the risk of HIV infection [1–4]. However, alternative forms of PrEP are needed, as oral PrEP did not demonstrate benefit in young women in Southern Africa in two large efficacy trials [5, 6]. Qualitative studies have revealed barriers to adherence to daily pills in this population, one of the foremost being the stigma associated with oral antiretrovirals [7]. Long-acting injectable therapy is an alternative form of PrEP that can overcome a number of these barriers and therefore may be more desirable and efficacious for certain populations [8].

Broadly neutralizing anti-HIV-1 antibodies (bNAbs) demonstrate excellent breadth and potency against diverse HIV-1 strains, and are currently being developed for the prevention and treatment of HIV-1 infection [9]. Antibodies typically have significantly longer half-lives than small molecule drugs, which allows for infrequent dosing in the outpatient setting. 3BNC117 and 10–1074 are bNAbs that target different epitopes on the HIV-1 envelope spike [10, 11]. In combination, they neutralize up to 90% of diverse HIV-1 strains in vitro at an IC_80_ < 5 μg/ml, and up to 85% of strains at an IC_80_ < 1 μg/ml [12]. 3BNC117 and 10–1074 have been shown to suppress viremia in animal models [13–15] and clinical trials [16–18]. When administered in the setting of analytical treatment interruption, the combination of 3BNC117 and 10–1074 delayed viral rebound for a median of 21 weeks in individuals harboring sensitive viruses [19]. Challenge studies performed in macaques demonstrated that a single intravenous infusion of 3BNC117 or 10–1074 protected against weekly SHIV_AD8_ challenge for a median of 13 and 12.5 weeks, respectively [20]. Additionally, a single subcutaneous injection of the combination of longer-acting variants of these antibodies resulted in protection for a median of 20 weeks, with one animal protected from weekly challenges for 37 weeks [21]. The combination of 3BNC117 and 10–1074 therefore represents a promising approach as a long-acting injectable modality for PrEP.

When administered alone in prior open-label studies, intravenous infusions of 3BNC117 and 10–1074 were well-tolerated and demonstrated half-lives of approximately 17 days and 24 days, respectively, in HIV-uninfected individuals [16, 17]. In this randomized, double-blind, placebo-controlled study, we assessed the safety, tolerability, pharmacokinetics, serum neutralizing activity, and immunogenicity of the combination of 3BNC117 and 10–1074 administered intravenously in healthy adults.

## Materials and methods

### Study design and participants

This phase 1, randomized, placebo-controlled, double-blind study was performed at The Rockefeller University Hospital in New York, NY, USA. The study protocol (YCO-0899) was approved by The Rockefeller University institutional review board. The study was conducted in accordance with the International Council for Harmonisation Good Clinical Practice guidelines and all applicable local regulatory requirements and laws. The study is registered with ClinicalTrials.gov, number NCT02824536.

The study included three groups each consisting of eight participants, six of whom received the study drugs and two of whom received placebo. Participants in Group 1 received an intravenous infusion of 3BNC117 immediately followed by 10–1074 at day 0 at a dose of 10 mg/kg. Participants in Groups 2 and 3 received three infusions of each antibody 8 weeks apart (weeks 0, 8, and 16) at a dose of 3 mg/kg and 10 mg/kg, respectively. These doses were selected because 3BNC117 and 10–1074 are being developed for subcutaneous administration in HIV-uninfected individuals, which will limit the maximum dose of each antibody to approximately 3 mg/kg. The higher dose of 10 mg/kg was evaluated to collect additional safety data. Following the enrollment of each group, enrollment in subsequent groups only began after review of day 28 safety data from all participants in the previous group. If greater than one grade 3 or higher adverse event deemed probably or definitely related to the study drugs occurred, the next group would not be enrolled before review of the study data by the Safety Monitoring Committee.

Participants provided written informed consent before screening. Eligible participants were healthy adults aged 18–65 years who were amenable to HIV risk reduction counseling and who agreed to use two methods of contraception for the duration of the study. Exclusion criteria included HIV infection, hepatitis B or C infection, history of sexually transmitted disease within 12 months prior to enrollment, any clinically significant medical condition that in the opinion of the investigator would preclude participation, vaccination within 14 days prior to infusion, pregnancy or lactation, receipt of any experimental HIV vaccine, history of severe reaction to a vaccine or drug or history of severe allergic reaction, participation in another clinical study of an investigational product within the past 12 weeks, and abnormalities on clinical laboratory assessments.

### Randomization and blinding

Randomized treatment assignments were generated by The Rockefeller University Hospital pharmacy. Randomization was generated using SAS 9.4. Each antibody infusion preparation was provided to study nurses for infusion under a coded, masked identification. The placebo (sterile saline) and active product preparations were indistinguishable. Nurses, study staff, investigators, and participants were blinded to the identity of the preparation.

### Procedures

A screening visit occurred within 49 days before enrollment, during which written informed consent was obtained. On the infusion days, 3BNC117 was administered first, and the 10–1074 infusion was begun once the 3BNC117 infusion was complete. Each antibody was diluted in normal saline to a total volume of 100 ml and administered over 60 minutes. Placebo infusions consisted of 100 ml normal saline. Study participants were followed for 24 weeks after the last antibody infusions. Assessments occurred at week 0, day 2, weeks 1, 2, 4, 8, 12, 16, 20 and 24 in Group 1, and at week 0, day 2, weeks 1, 2, 4, 8, day 58, weeks 9, 10, 12, 16, day 114, weeks 17, 18, 20, 24, 28, 32, 36 and 40 in Groups 2 and 3. Assessments included physical examinations and vital sign measurements, clinical and research laboratory tests, assessments of adverse events, and review of concomitant medications. In addition, serum samples were collected during all study visits, including samples collected prior to and at the end of each antibody infusion, for measurement of antibody concentrations. Solicited adverse events were recorded for 1 week following infusions. Unsolicited adverse events were recorded at all assessments. The Toxicity Grading Scale for Healthy Adult and Adolescent Volunteers Enrolled in Preventive Vaccine Clinical Trials (2007) was used to grade adverse events. The Common Terminology Criteria for Adverse Events (CTCAE) v4.03 grading scale was used to grade adverse events determined to be infusion reactions.

### Outcomes

The primary outcomes were safety, defined as the rate of signs, symptoms, and laboratory abnormalities, in addition to local and systemic reactogenicity adverse events 1 week after the combination of 3BNC117 and 10–1074 infusion in all study groups, and pharmacokinetics. The secondary outcomes were the frequency and concentrations of induced anti-drug antibodies, and the rate of signs, symptoms and laboratory abnormalities that occurred during study follow up after 3BNC117 and 10–1074 infusions in all study groups.

### TZM-bl neutralization assay to measure 3BNC117 and 10–1074 serum concentrations

The TZM-bl neutralization assay was performed as previously described [22]. Briefly, serum samples were heat-inactivated for one hour at 56°C and tested using a primary 1:20 dilution and a 5-fold titration series against HIV-1 Env pseudoviruses Q769.d22 and X2088_c9. These pseudoviruses are highly sensitive to neutralization by either 3BNC117 or 10–1074, respectively, and fully resistant against the other antibody. 3BNC117 and 10–1074 clinical drug products were tested in parallel at a starting concentration of 10 μg/ml with a 5-fold titration series. Serum concentrations of 3BNC117 and 10–1074 were calculated by multiplying the determined ID_50_ titer of the respective serum sample and the determined IC_50_ concentration of each monoclonal standard antibody. Viruses pseudotyped with the envelope protein murine leukemia virus (MuLV) were used as negative control. Based on the nature of this assay, measured ID_50_ titers may have < 3-fold variability[22].

### ELISA-based measurement of 3BNC117 and 10–1074 serum concentrations

3BNC117 and 10–1074 serum concentrations were determined by a validated sandwich ELISA from the average of duplicate wells with intraday and interday CV < 20%. High bind polystyrene plates were coated overnight at 2–8°C with 4 μg/ml of an anti-idiotypic antibody specifically recognizing 3BNC117 (anti-ID 1F1-2E3 mAb) or 2 μg/ml of an anti-idiotypic antibody specifically recognizing 10–1074 (anti-ID 3A1-4E11 mAb). After washing, plates were blocked with 5% Milk Blotto (w/v), 5% NGS (v/v), and 0.05% Tween 20 (v/v) in PBS. Serum samples, QCs and standards were added (1:50 minimum dilution in 5% Milk Blotto (w/v), 5% NGS (v/v), and 0.05% Tween 20 (v/v) in PBS) and incubated at room temperature. A horseradish peroxidase (HRP)-conjugated mouse anti-human IgG kappa-chain-specific antibody (Abcam) was used to detect 3BNC117 and an HRP-conjugated goat antihuman IgG Fc-specific antibody (Jackson ImmunoResearch) to detect 10–1074. For detection, the HRP substrate tetra-methylbenzidine was added. A 5-PL curve fitting-algorithm (Softmax Pro, v. 5.4.5) was used to calculate serum 3BNC117 and 10–1074 concentrations from respective standard curves run on the same plate. Standards and positive controls were created from the drug product lots of 3BNC117 and 10–1074 that were used in the clinical study. Starting concentrations on the standard curve were determined using A280 absorbance with an extinction coefficient of 1.39. Additionally, high, medium and low single concentration quality controls were made by spiking clinical drug product into HIV-1 seronegative human serum diluted in assay diluent, covering the range of sample dilution factors in the assay. Acceptable recovery ranges for spiked QC samples were set to 70–130% of expected concentration. The capture anti-idiotypic mAbs were produced in a stable hybridoma cell line (Duke Protein Production Facility) [16]. The lower limit of quantitation was determined to be 0.78 μg/ml and 0.41 μg/ml for the 3BNC117 ELISA and 10–1074 ELISA, respectively.

### Measurement of anti-drug antibodies

Electrochemiluminesnce bridging assays to detect anti-drug antibodies (ADAs) against either 3BNC117 or 10–1074 were developed and qualified. Briefly, covalently biotinylated and sulfo-tagged drug product were combined, mixed with test samples, and incubated at room temperature. After the incubation, the mixture was added to a proprietary streptavidin-functionalized plate (MSD), washed, and the presence of ADA responses, as indicated by the presence of ternary or “bridged” complexes of biotinylated and sulfo-tagged drug, was detected using an MSD SQ120 reader. Samples were characterized using a tiered approach to first define positivity in a screening assay (Tier 1), to then define the specificity of positive samples (Tier 2), and lastly to titer the responses of samples confirmed as specific (Tier 3). In Tier 1, a positivity cutpoint established during assay qualification (95^th^ percentile of signal observed among treatment naïve donors) was utilized to determine whether a sample was ADA screening assay positive. In Tier 2, specificity was determined by adding 10 μg/ml (final concentration in neat serum) of unlabeled drug and observing a decrease in signal greater than the 99^th^ percentile in signal reduction observed among treatment naïve donors as determined during qualification. Finally, titering was performed in three-fold dilution steps and the titer of each confirmed response was defined as the lowest dilution that remained above the positivity cutpoint. Each sample was tested in duplicate along with a panel of positive and negative controls, and results were evaluated according to predetermined acceptance criteria. Free drug has the potential to compete with labeled drug in ADA assays and lead to false negatives. In the presence of free drug concentrations at or below 10 μg/ml for 3BNC117 and 70 μg/ml for 10–1074, the ability to detect monoclonal anti-idiotypic antibody was confirmed to meet the FDA recommended sensitivity of 100 ng/ml. ADAs were defined as either treatment-induced, treatment-boosted, or treatment-independent. Treatment-induced responses were defined as those in which a participant was ADA negative at baseline and became ADA positive for at least one time point after the administration of drug. Treatment-boosted responses were defined as those in which a participant was ADA positive at baseline but one or more time points after the administration of drug was ADA positive with at least a three-fold increase in titer relative to baseline. Finally, treatment-independent responses were defined as those in which a participant was ADA positive at baseline and all post-treatment samples had a titer equal or less than the baseline titer.

### Functional anti-drug antibodies

A modification of the validated TZM-bl neutralization assay to quantitatively measure ADA as a function of reduced neutralizing activity of a target bNAb was optimized and qualified for both 10–1074 and 3BNC117. For measurements of ADA, serum samples from clinical trial participants were titrated in the presence of a single fixed concentration of bNAb, and neutralizing activity against a sensitive HIV-1 Env pseudovirus was assessed. ADA assays for 3BNC117 were performed using virus Q842.d12 and a fixed concentration of bNAb at 0.03 μg/ml, and assays for measuring 10–1074 ADA were performed using virus X2088_c9 and a fixed concentration of bNAb at 0.016 μg/ml. Results were expressed as the serum dilution in which 50% of the baseline bNAb neutralizing activity is inhibited (ID_50_ ADA titer). Mouse anti-idiotype antibodies against either 3BNC117 or 10–1074 were used as positive ADA controls.

### Statistical analysis

Power calculations were not performed for this phase 1 study. Categorical and continuous data were summarized descriptively. Safety data from participants receiving placebo in different groups were pooled.

### Pharmacokinetics analysis

All participants who received the study products were included in the pharmacokinetic (PK) analyses. Antibody serum concentrations measured by binding ELISA were used for PK analyses. Clearance from the central compartment (CL), volume of the central compartment (Vc), inter-compartmental distribution clearance (Q), and volume of the peripheral compartment (Vp) were computed using individual concentration two-compartmental models, a commonly used method to evaluate PK parameters of monoclonal antibodies [23, 24], in NONMEM 7.4.2. Given the estimated parameters (CL, Vc, Q, and Vp), distribution and elimination phase half-life estimates were computed for each individual. The area under the concentration curve (AUC) was calculated by integrating the area under the predicted concentration curves from time 0 to infinity. Peak concentration (Cmax) was computed as the maximum observed concentration.

Additional analyses were performed to compare pharmacokinetic parameters when each bNAb was administered alone (during two prior studies: ClinicalTrials.gov numbers NCT02018510 [16]) and NCT02511990 [17]) or in combination during this reported study. For each bNAb separately, elimination half-life was modeled using a full linear regression for each trial individually and across trials pooling participants. The potential effect of additional variables on pharmacokinetics parameters were also explored. These included: dose level (log_10_), number of doses, BMI, age (median-adjusted) and gender.

Lin’s concordance correlation coefficient was calculated for 3BNC117 and 10–1074 between log- transformed antibody concentrations measured via Tzm.bl assay (of X2088_c9 and Q769.d22, respectively) and binding ELISA across all time points for all participants. The concordance correlation coefficient combines measures of both precision and accuracy to determine how far the observed data deviate from the line of perfect concordance (that is, the line at 45 degrees on a square scatter plot).

## Results and discussion

**Fig 1:**
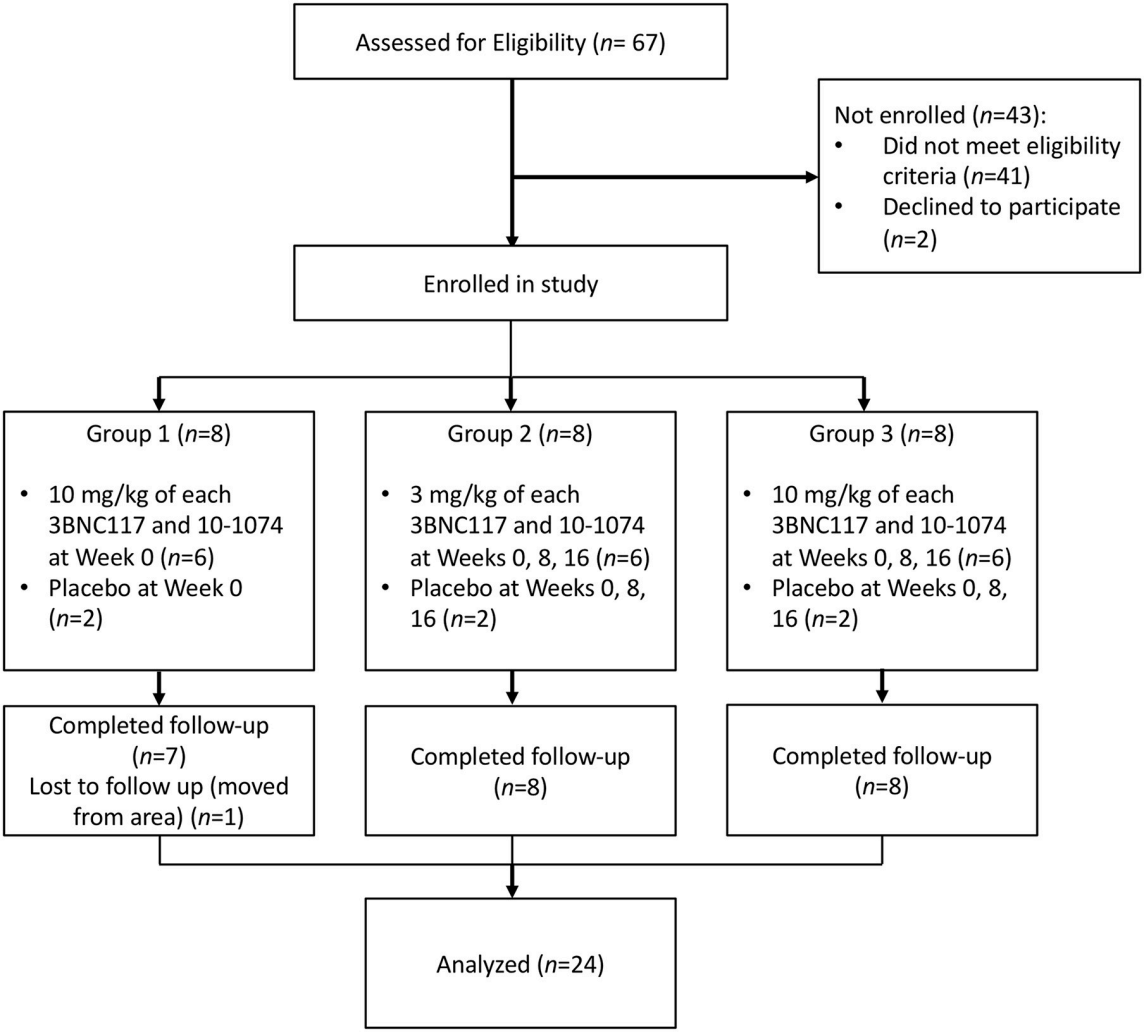
Participant flow diagram.

Between June 23, 2016 and February 27, 2017, 67 individuals were screened for the study. Of these, 24 were sequentially enrolled into the 3 groups. Of those not enrolled, 41 did not meet eligibility criteria and 2 declined to participate. In each group, six participants were randomized to receive 3BNC117 and 10–1074, and two were randomized to receive placebo (Fig 1). Twenty-three participants completed all study visits, with one participant in Group 1 being lost to follow up after the week 8 visit. One participant in Group 2 did not receive the 10–1074 infusion at week 8 or the week 16 3BNC117 and 10–1074 infusions. Available data from both of these participants were included in the analysis. Participant demographics are shown in Table 1 and S1 Table.

**Table 1:** Participant demographics.

	Group 1 3BNC117 + 10–1074 10 mg/kg x1 (n = 8)	Group 2 3BNC117 + 10–1074 3 mg/kg x 3 (n = 8)	Group 3 3BNC117 + 10–1074 10 mg/kg x 3 (n = 8)
Median age in years (range)	45 (34–63)	41.5 (24–54)	31.5 (21–53)
Men, n (%)	8 (100%)	6 (75%)	5 (63%)
Women, n (%)	0 (0%)	2 (25%)	3 (37%)
Race			
Black, n (%)	3 (37%)	6 (75%)	5 (63%)
White, n (%)	4 (50%)	0 (0%)	3 (37%)
Multiple/other, n (%)	1 (13%)	2 (25%)	0 (0%)
Hispanic, n (%)	2 (25%)	2 (25%)	2 (25%)
Median body mass index (range)	29.9 (20.6–31)	26.2 (21–44.5)	24.4 (19.5–38)

Adverse events are summarized in Table 2. Four reactogenicity adverse events occurred within 1 week following infusion. Three were graded as mild: headache in a participant who received the study products, headache in a participant who received placebo, and malaise/fatigue in a participant who received the study products. The fourth, graded as moderate, was fever in a participant who was randomized to receive the study products at 3 mg/kg. The participant was found to have a temperature of 39.5° C immediately following the completion of the second dose of 3BNC117 at week 8. This participant received both antibodies without incident at week 0. Immediately prior to the week 8 infusion, he had normal vital signs and was feeling well. The elevated temperature was confirmed on repeat measurements, during which time the participant had no complaints, including no sensation of feverishness or chills. Acetaminophen was administered with resolution of the fever over the next few hours. The decision was made to discontinue further infusions for this participant.

**Table 2:** Summary of adverse events.

	3BNC117 + 10–1074 10 mg/kg x 1 n = 6	3BNC117 + 10–1074 3 mg/kg x 3 n = 6	3BNC117 + 10–1074 10 mg/kg x 3 n = 6	All 3BNC117 + 10–1074 n = 18	Placebo n = 6
Participants with any AE	4 (66%)	3 (50%)	4 (66%)	11 (61%)	5 (83%)
Number of reactogenicity AEs	1	2	0	3	1
Number of non-reactogenicity AEs	5	3	4	12	8
Number of SAEs	0	0	2	2	0
AE listings					
**Severe**					
Urinary retention	0	0	1	1	0
Suicidal ideation	0	0	1	1	0
Anemia	0	0	1	1	2
Total				3	2
**Moderate**					
Elevated creatinine	0	1	0	1	0
Elevated total bilirubin	1	0	1	2	0
Anemia	0	0	0	0	1
Total				3	1
**Mild**					
Headache	1	1	0	2	2
Malaise/fatigue	1	0	0	1	0
Fever	0	1	0	1	0
Increased lacrimation	1	0	0	1	0
Upper respiratory tract infection	1	2	1	4	2
Epistaxis	1	0	0	1	0
Vertigo	0	0	1	1	0
Shoulder pain	0	0	0	0	1
Anemia	0	0	0	0	1
**Total**				11	6

There were 12 non-reactogenicity adverse events in participants who received the study products, and 8 in participants who received placebo. There were two SAEs reported, both of which occurred in the same participant and were considered unrelated to the infusions. The participant had a history of urinary retention and depression, and the SAEs consisted of hospitalizations for urinary retention and suicidal ideation. Three non-SAE (serious adverse events) adverse events were graded as severe and considered unrelated to the infusions: anemia in a participant who received the study products, and anemia in 2 participants who received placebo. There were four adverse events graded as moderate and 17 adverse events graded as mild. The most frequent mild or moderate adverse events were upper respiratory tract infection (6 events), headache (4 events), and increased total bilirubin (2 events).

**Fig 2:**
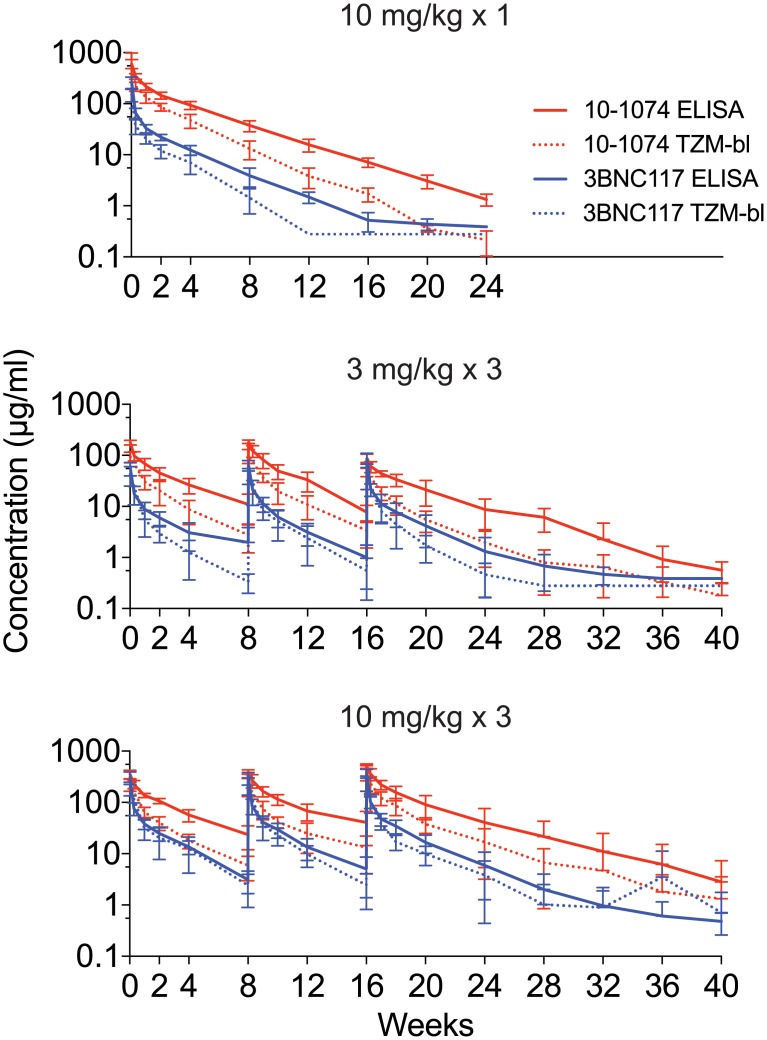
Serum concentrations of 3BNC117 and 10–1074 by group. Serum concentrations (μg/ml) of 3BNC117 (blue) and 10–1074 (red) in participants after a single infusion of 3BNC117 and 10–1074 (10 mg/kg of each antibody) (top panel), and three infusions of 3 mg/kg (middle panel) or 10 mg/kg of each antibody (bottom panel) given every eight weeks. bNAb concentrations were determined by TZM-bl assay (dotted lines) and ELISA (solid lines). Lines indicate arithmetic mean concentration and whiskers indicate standard deviation.

Concentrations of 3BNC117 and 10–1074 were measured by ELISA and TZM-bl neutralization assay, with general concordance between the two assays (correlation coefficients in Groups 1, 2 and 3 for 3BNC117 concentrations were 0.85, 0.88 and 0.91, respectively, and for 10–1074 concentrations they were 0.78, 0.65 and 0.64, respectively) (Fig 2, S2 Table). One participant in Group 3 demonstrated an increase in serum neutralizing activity against pseudovirus Q769.d22 in the TZM-bl assay at weeks 32–40, when 3BNC117 concentrations were undetectable by ELISA, for unclear reasons. The pharmacokinetic parameters of clearance (CL), peak concentration (Cmax), distribution half-life (t_1/2_), elimination half-life (t_1/2_), and area under the concentration curve (AUC) were determined for each antibody in each group utilizing the ELISA data (Table 3, S3 Table). Overall, the mean elimination half-life for 3BNC117 was found to be 16.4 ± 4.6 days, and the mean elimination half-life for 10–1074 was found to be 23.0 ± 5.4 days. There were no significant associations (p-values > 0.05) between elimination half-life for either antibody according to dose level administered, or following single or repeated doses (Table 4).

**Table 3:** Pharmacokinetic parameters of 3BNC117 and 10–1074 administered in combination.

	3BNC117			10–1074		
	10 mg/kg x 1	3 mg/kg x 3	10 mg/kg x 3	10 mg/kg x 1	3 mg/kg x 3	10 mg/kg x 3
Number of participants	6	6	6	6	6	6
Clearance (L/day)	0.75 (0.17)	0.76 (0.19)	0.61 (0.19)	0.11 (0.02)	0.12 (0.03)	0.14 (0.05)
Cmax (μg/ml)	270.59 (71.12)	88.38 (19.15)	385.92 (67.89)	608.75 (122.0)	189 (17.4)	512.62 (50.69)
AUC (μg × hr/ml)	1204.26 (252.04)	950.63 (440.35)	4121.43 (812.78)	8290.97 (1716.41)	5441.40 (2448.78)	18791.80 (5223.4)
Distribution t_1/2_ (days)	0.67 (0.28)	0.63 (0.11)	0.52 (0.14)	2.27 (1.38)	1.79 (0.84)	2.62 (1.33)
Elimination t_1/2_ (days)	18.39 (2.64)	14.20 (4.25)	16.64 (5.92)	23.20 (1.17)	20.71 (2.91)	24.99 (8.84)
Median Elimination t_1/2_ (days)	18.04	13.73	15.02	23.34	19.87	22.24

Note: The table shows mean, unless otherwise noted, and standard deviations (in parenthesis) for each of the PK parameters presented.

**Table 4:** Analysis of associations between selected group variables and antibody half-lives for 3BNC117 and 10–1074 when administered in combination.

bnAb	Term	Estimate	Std. Error	95% CI	p-value
3BNC117	(Intercept)	16.43	8.06	(-1.14, 34)	0.064
	Log_10_ dose	3.25	5.34	(-8.38, 14.87)	0.554
	Number of Doses	-3.30	3.20	(-10.28, 3.67)	0.323
	BMI	-0.01	0.22	(-0.48, 0.46)	0.967
	Gender	-1.97	3.68	(-9.99, 6.05)	0.603
	Median-adjusted age	-0.13	0.12	(-0.38, 0.12)	0.269
10–1074	(Intercept)	23.97	9.63	(2.98, 44.95)	0.028
	Log_10_ dose	7.93	6.37	(-5.95, 21.81)	0.237
	Number of Doses	0.83	3.82	(-7.5, 9.16)	0.832
	BMI	-0.28	0.26	(-0.85, 0.28)	0.299
	Gender	-3.50	4.40	(-13.08, 6.08)	0.441
	Median-adjusted age	-0.07	0.14	(-0.37, 0.23)	0.618

**Fig 3:**
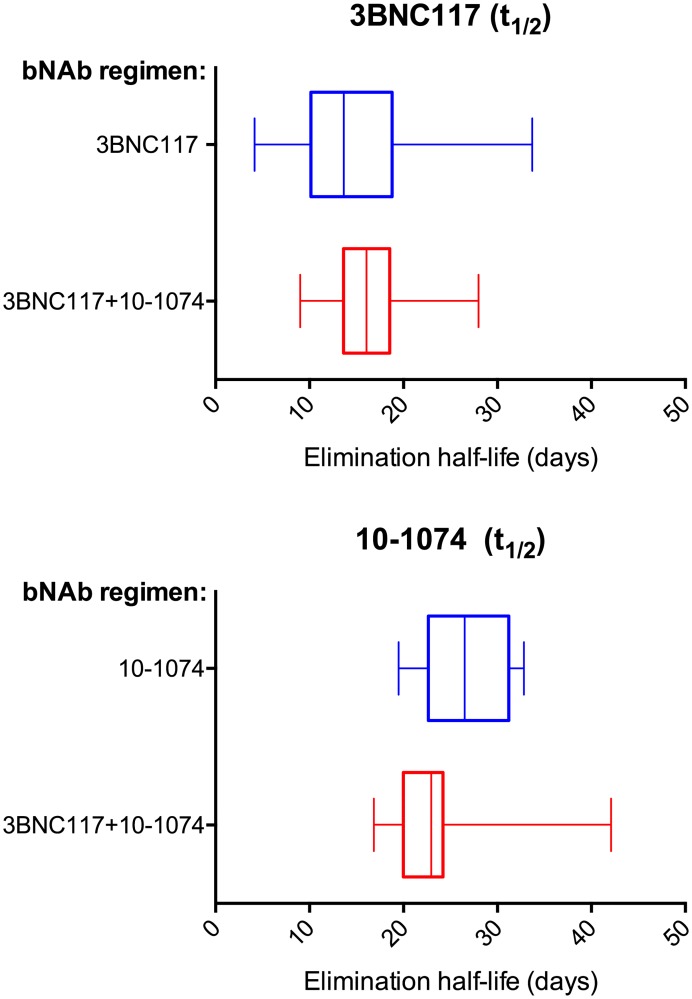
Elimination half-lives of 3BNC117 and 10–1074 when administered alone and in combination. (Top panel) shows 3BNC117 half-lives in HIV-uninfected participants after single antibody (blue, from ClinicalTrials.gov number NCT02018510 [16]), n = 22) or combination (red, current trial n = 18) administration. (Bottom panel) shows 10–1074 half-lives in HIV-uninfected participants after single antibody (green, from ClinicalTrials.gov number NCT02511990 [17], n = 14) or combination (red, current trial n = 18) administration. Boxes illustrate the interquartile range. Vertical lines inside the boxes represent the median half-lives and whiskers represent minimum and maximum values.

ELISA data from HIV-uninfected participants enrolled in two prior clinical trials evaluating 3BNC117 [16] (ClinicalTrials.gov number NCT02018510) or 10–1074 [17] (ClinicalTrials.gov number NCT02511990) were reanalyzed and compared with the ELISA data from the 18 participants who received the antibodies in combination in the current trial. In the 3BNC117 only trial, 22 HIV-uninfected participants received one or two intravenous infusions of the antibody at doses ranging from 1 to 30 mg/kg, and in the 10–1074 only trial 14 HIV-uninfected participants received a single intravenous infusion of the antibody at doses ranging from 3 to 30 mg/kg (Table 5). Single versus co-administration of the antibodies did not significantly influence elimination half-lives of 3BNC117 (mean half-life: single 19.18 ± 7.08 days vs. co-administration 16.4 ± 4.6 days, p 0.484) or 10–1074 (mean half-life: single 26.7 ± 4.5 days vs. co-administration 23.0 ± 5.4 days, p 0.398) (Fig 3, Table 6).

**Table 5:** Pharmacokinetic parameters of 3BNC117 and 10–1074 when administered as single antibodies.

bNAb	Dose, Route	N	Mean (SD) (days)	Median (min, max) (days)
3BNC117	1 mg/kg x 1, IV	3	17.64 (10.11)	14.63 (9.37, 28.9)
	3 mg/kg x 1, IV	3	24.53 (9.04)	24.26 (15.63, 33.7)
	10 mg/kg x 1, IV	3	17.62 (8.13)	18.79 (8.97, 25.11)
	30 mg/kg x 1, IV	5	17.5 (8.22)	15.6 (10.67, 31.73)
	10 mg/kg x 2 (weeks 0–12), IV	3	17.01 (2.33)	16.85 (14.77, 19.41)
	30 mg/kg x 2 (weeks 0–12)	5	22.13 (4.57)	19.9 (19.13, 29.97)
	***All groups***	***22***	***19*.*18 (7*.*08)***	***18*.*96 (8*.*97*, *33*.*7)***
10–1074	3 mg/kg x 1, IV	3	26.41 (3.07)	27.41 (22.97, 28.84)
	10 mg/kg x 1, IV	3	27.57 (4.59)	25.6 (24.3, 32.82)
	30 mg/kg x 1, IV	3	24.31 (6.49)	21.77 (19.48, 31.68)
	30 mg/kg x 1, IV	5	27.77 (4.72)	29.61 (20.83, 32.22)
	***All groups***	***14***	***26*.*69 (4*.*47)***	***26*.*5 (19*.*48*, *32*.*82)***

**Table 6:** Analysis of associations between selected group variables and antibody half-lives for 3BNC117 and 10–1074 when administered in combination and when administered as single antibodies.

Analyte	Term	Estimate	Std. Error	95% CI	p-value
3BNC117	(Intercept)	17.67	5.69	(6.09, 29.24)	0.004
	Log_10_ dose	1.84	2.43	(-3.1, 6.78)	0.454
	Number of Doses	-2.35	2.17	(-6.77, 2.07)	0.288
	BMI	-0.07	0.20	(-0.47, 0.34)	0.743
	Gender	5.07	2.66	(-0.34, 10.49)	0.065
	Median-adjusted age	-0.01	0.08	(-0.18, 0.16)	0.943
	Administration	1.48	2.09	(-2.77, 5.74)	0.484
10–1074	(Intercept)	33.54	5.85	(21.48, 45.59)	0.000
	Log_10_ dose	2.09	2.75	(-3.58, 7.76)	0.455
	Number of Doses	-2.02	2.68	(-7.54, 3.5)	0.459
	BMI	-0.40	0.18	(-0.77, -0.02)	0.040
	Gender	-0.75	2.44	(-5.79, 4.28)	0.760
	Median-adjusted age	-0.12	0.09	(-0.3, 0.06)	0.169
	Administration	2.15	2.51	(-3.01, 7.31)	0.398

**Fig 4:**
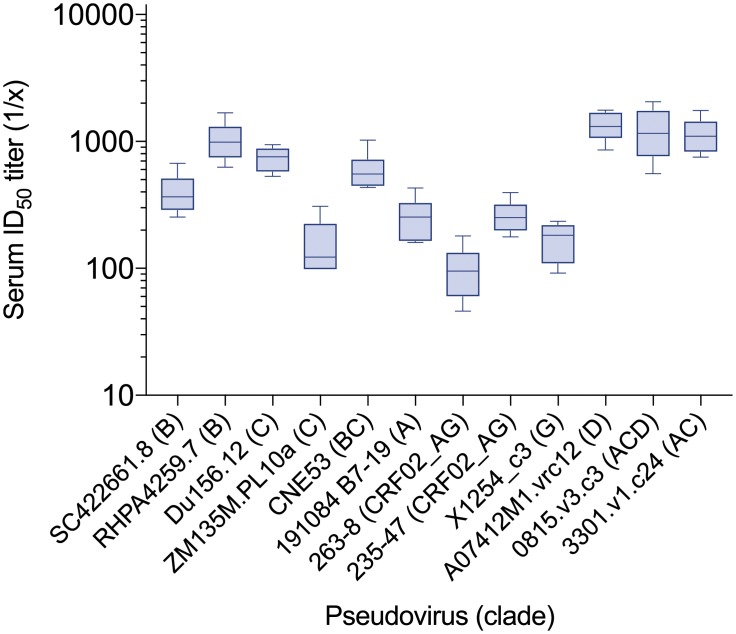
Serum neutralization of diverse pseudoviruses. Week 2 serum from participants who received the antibodies at 3 mg/kg was tested against 12 pseudoviruses from diverse clades previously found to be sensitive to 3BNC117 and 10–1074. X-axis shows calculated serum ID_50_ titers for each tested pseudovirus (y-axis). Boxes illustrate the interquartile range. Horizontal lines inside the boxes represent the median half-lives and whiskers represent minimum and maximum values.

The neutralizing activity of the antibodies in serum was evaluated against a larger panel of viruses in the 3 mg/kg dose group 2 weeks after the first infusion, when concentrations of both antibodies were still greater than 5 μg/ml, which is a concentration that provided approximately 90% coverage against HIV-1 pseudoviruses from multiple clades *in vitro* [12]. The average serum concentrations at this time point, measured by ELISA, were 6.0 μg/ml for 3BNC117 and 45.2 μg/ml for 10–1074. Serum was tested against a diverse panel of 12 tier 2 pseudoviruses known to be sensitive to 3BNC117 and 10–1074. All serum samples had detectable neutralizing activity against all 12 pseudoviruses, with an overall geometric mean ID_50_ titer of 432.9 (Fig 4, S4 Table).

**Fig 5:**
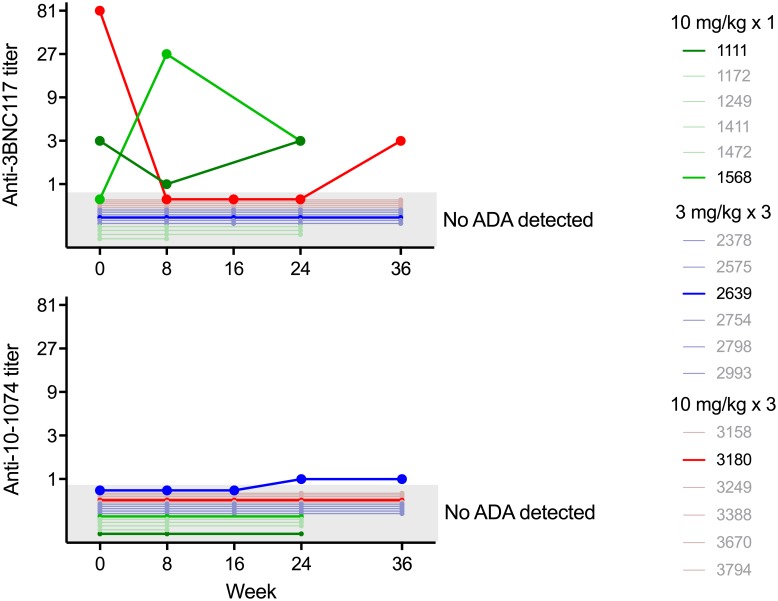
Anti-drug antibody responses. Specific anti-3BNC117 antibody titers (top panel) and anti-10-1074 antibody titers (bottom panel) at selected time points following the administration of 3BNC117 plus 10–1074 are displayed for individuals participants. Each line represents participants enrolled in each of the three study groups: green, group 1 (single dose of 3BNC117 and 10–1074 at 10 mg/kg, IV); blue, group 2 (repeated doses of 3BNC117 and 10–1074 at 3 mg/kg, IV); red, group 3 (repeated doses of 3BNC117 and 10–1074 at 10 mg/kg, IV). For each participant, the time points that were tested for the presence of anti-drug antibodies are shown. Samples for which a specific anti-drug antibody response was not detected are indicated in the shaded grey area.

Serum samples were tested for the presence of anti-drug antibodies. Samples from weeks 0, 8, and 24 were tested from participants who received a single dose of the study products, and samples from weeks 0, 8, 16, 24, and 36 were tested from participants who received 3 doses of the study products. Serum antibody concentrations at these time points were below 10 μg/ml for 3BNC117 and 70 μg/ml for 10–1074 for 75 of the 77 samples tested, thus ensuring that the test met the FDA-recommended level of sensitivity to detect anti-idiotypic antibodies for the vast majority of samples. Nine serum samples were found to be positive for anti-3BNC117 antibodies in the Tier 1 screening assay. Seven of the nine samples, deriving from three participants, were confirmed to be specific anti-3BNC117 antibody responses in Tier 2 testing (Fig 5, top panel, S5 Table). Two of these three participants (1111, 3180) had treatment-independent responses, with positivity already present at baseline and all post-infusion samples demonstrating a titer equal to or less than the baseline titer. The third participant (1568) had a treatment-induced response, with maximum anti-3BNC117 antibody titers of 1:27 at week 8, which decreased to 1:3 by week 24 (Fig 5, top panel). Two serum samples from a single participant were found to be positive for anti-10-1074 antibodies using the Tier 1 screening assay (2639), and confirmed to be specific in the Tier 2 assay. This participant demonstrated a treatment-induced response, with a maximum titer of 1:1 (Fig 5, bottom panel, S5 Table). No participant with ADA responses had adverse events considered related to the infusions, or changes in elimination kinetics. ADA-positive serum samples were negative for ADA in a functional ADA neutralization assay (Table 7), demonstrating that the concentrations of ADA were not sufficient to interfere with 3BNC117 or 10–1074 neutralization.

**Table 7:** Functional ADA assay results in participants with specific ADA responses to 3BNC117 or 10–1074.

Study ID	Time point	ID_50_ Serum Titer in TZM.bl cells (1/x)	
		Anti-3BNC117 ADA	Anti-10-1074 ADA
		Pseudovirus Q842.d12	Pseudovirus X2088_c9
1111	Week 0	<20	<20
	Week 8	<20	<20
	Week 24	<20	<20
1568	Week 0	<20	<20
	Week 8	<20	<20
	Week 24	<20	<20
2639	Week 0	27	<20
	Week 8	<20	<20
	Week 24	<20	<20
	Week 36	23	<20
3180	Week 0	<20	28
	Week 8	<20	<20
	Week 24	<20	<20
	Week 36	<20	<20

While there has been a decline in the overall global incidence of HIV infection, particular regions and populations remain highly affected by the epidemic. In KwaZulu-Natal, South Africa, the prevalence of HIV infection among pregnant women is approximately 40% [25]. In lieu of an HIV vaccine, pre-exposure prophylaxis holds significant promise as a means to reduce the incidence of HIV in these populations. Long-acting injectable PrEP represents a novel strategy that may overcome barriers associated with the uptake and efficacy of oral PrEP. The long-acting integrase strand transfer inhibitor cabotegravir, administered intramuscularly every 8 weeks, is currently being evaluated in phase 3 trials in men and transgender women who have sex with men, and in sub-Saharan African women (HPTN 083/084, ClinicalTrials.gov numbers NCT02720094 and NCT03164564). Additionally, the bNAb VRC01, administered intravenously every 8 weeks, is being evaluated in two phase 2b trials in men and transgender persons who have sex with men, and in sub-Saharan African women (AMP study, HVTN 703/HPTN 081, HVTN 704/HPTN 085, ClinicalTrials.gov numbers NCT02568215 and NCT02716675).

In the current study, we evaluated the safety and pharmacokinetics of the combination of 2 bNAbs, 3BNC117 and 10–1074, which target non-overlapping epitopes on the HIV envelope. The combination of two antibodies that target different epitopes on the HIV envelope has the potential to provide broader coverage of circulating HIV strains compared to a single antibody. Intravenous infusions of the combination of 3BNC117 and 10–1074 were generally well tolerated, their respective half-lives were not altered when administered in combination, and their neutralizing activity was maintained in serum. Minimal immunogenicity was elicited, even in the setting of multiple doses. Moreover, the anti-antibody responses that were elicited did not impact antibody half-life or result in adverse events.

The doses of each antibody evaluated in this study, 3 mg/kg and 10 mg/kg, are lower than the dose of 30 mg/kg administered in a recent trial that evaluated the combination of these 2 antibodies in HIV-infected individuals [18, 19]. These lower doses were selected because *in vitro* neutralization activity data and results from SHIV challenge experiments in non-human primates suggest that the antibody concentrations required for protection are lower than concentrations required to maintain or achieve viral suppression during HIV-1 infection [18, 21, 26]. 3BNC117 and 10–1074 are being developed for subcutaneous administration for the indication of prophylaxis, which will limit the maximum dose of each antibody but facilitate their use in sub-Saharan Africa. The elimination half-lives of these antibodies are expected to be similar regardless of the route of administration [27, 28]. Considering that the half-life of 10–1074 is longer than that of 3BNC117, the dose of 10–1074 will need to be titrated to achieve similar trough concentrations. Variants of both 3BNC117 and 10–1074 with 2 amino acid mutations designed to extend half-life are currently being evaluated in clinical trials (ClinicalTrials.gov numbers NCT03254277 and NCT03554408). This set of mutations, designated “LS”, enhance antibody binding to FcRn receptors, which increases antibody recycling and prolongs half-life [29]. The LS mutations have been shown to increase the half-life of the bNAb VRC01 by more than 4-fold in a phase 1 trial in HIV-uninfected individuals [27]. In a study in macaques, 3BNC117-LS and 10-1074-LS extended the protective efficacy of each antibody against repeated low-dose SHIV_AD8_ rectal challenges [21]. In that study, the median antibody serum concentrations at infection breakthrough were 0.13 μg/ml for 10-1074-LS and 0.20 μg/ml for 3BNC117-LS, and correlated with the measured IC_80_ against the challenge virus in TZM-bl neutralization assays. Results from this study support the clinical evaluation of the LS variants of 3BNC117 and 10–1074.

While bNAbs have been shown to prevent HIV or SHIV infection in animal models, the ability of bNAbs to prevent HIV infection in humans remains to be determined. Results from non-human primate challenge studies suggest protection is related to the potency and half-life of the antibody [20, 30]. The measured *in vitro* potency of anti-HIV-1 antibodies, including broadly neutralizing antibodies, has been shown to vary depending on the characteristics of the assay used [31–34]. The ongoing Antibody Mediated Prevention (AMP) study (www.ampstudy.org), which is evaluating the efficacy of VRC01, will provide important information regarding how the results of *in vitro* assays correlate with *in vivo* performance.

## Conclusions

In summary, the combination of 3BNC117 and 10–1074 was safe and generally well tolerated, demonstrated favorable pharmacokinetic parameters, and elicited minimal immunogenicity even over multiple administrations. These findings support the continued development of 3BNC117 and 10–1074 for the prevention of HIV-1 infection.

## Supporting information

S1 File CONSORT checklist. (DOC)

S2 File Original IRB approval document. (PDF)

S3 File Trial protocol. (PDF)

S1 Table Individual participant demographics. (PDF)

S2 Table 3BNC117 and 10–1074 Serum Levels. (PDF)

S3 Table Pharmacokinetic paramenters of 3BNC117 and 10–1074 administered in combination. (PDF)

S4 Table Serum neutralizing activity against a multi-clade pseudovirus panel. (PDF)

S5 Table Anti-drug antibody responses. (PDF)
