# Supplementary material for: Safety, pharmacokinetics, and immunogenicity of the combination of the broadly neutralizing anti-HIV-1 antibodies 3BNC117 and 10-1074 in healthy adults: A randomized, phase 1 study
Source: PLoS One. 2019 Aug 8;14(8):e0219142. doi: 10.1371/journal.pone.0219142 (PMC6687118; doi:10.1371/journal.pone.0219142)
Supplement: S3 File — (PDF) [file pone.0219142.s003.pdf]

A phase 1 study of the safety and pharmacokinetics of the combination of 3BNC117 and 10-1074 in HIV-uninfected adults

IND Sponsor:
The Rockefeller University
IND # 118,225

Principal Investigator:
Yehuda Cohen, MD

Version Date:
13 December 2017

Statement of Compliance

The clinical trial will be conducted in compliance with the protocol, with the International Conference on Harmonization Good Clinical Practice E6 (ICH-GCP), and with 45 CFR 46 and 21 CFR 50, 56 and 312. All protocol investigators have completed Protection of Human Subjects Training.

Signature Page 1

The signature below constitutes the approval of this protocol and the attachments, and provides the necessary assurances that this trial will be conducted according to all stipulations of the protocol, including all statements regarding confidentiality, and according to local legal and regulatory requirements and applicable U.S. federal regulations and ICH guidelines.

The Lead Principal Investigator (Protocol Chair) should sign Signature Page 1. A copy of this Signature Page 1 should be filed with the holder of the Regulatory documents and a copy should be maintained at the site.

Principal Investigator: ____
Print/Type

Signed: ____________________________ Date: ___________________
Name/Title

Table of Contents

1. Key Roles **10**
1.1 Study Site and associated Institutions **10**
1.2 Study Personnel **10**
1.1.2 Principal Investigator 10
1.1.3 Protocol Staff Personnel – Co-investigators 10
1.1.4 Consultants 11
2 Lay Summary **11**
3 Objectives and Rationale **11**
3.1 Overview **11**
3.1.1 Background 11
3.1.2 Clinical Safety of other Monoclonal Antibodies against Infectious Pathogens 12
3.2 Preclinical Studies with 3BNC117 and 10-1074 .... **14**
3.2.2 In Vitro Neutralizing Activity 14
3.2.3 In Vivo Activity in Humanized Mice and Non-human Primates 15
3.3 3BNC117 .... **17**
3.3.2 3BNC117 Drug Product 17
3.3.3 Preclinical Toxicity Studies with 3BNC117 17
3.3.4 Clinical Experience with 3BNC117 18
3.4 10-1074 .... **19**
3.4.2 10-1074 Drug Product 19
3.4.3 Preclinical Toxicity Studies with 10-1074 20
3.4.4 Clinical Experience with 10-1074 21
3.5 Objectives **21**
3.6 Outcomes **22**
Primary Outcomes: **22**
4 Study Design **22**
5 Study Population **23**
5.1 Inclusion Criteria **23**
5.2 Exclusion Criteria **23**
6 Methods and Procedures **24**
6.1 Screening Procedure and Study Visits **24**
6.1.1 Pre-Screening Questionnaire 24
6.1.2 Screening Visit 24
6.1.3 3BNC117/10-1074 Infusion Visit 25
6.1.4 Post-3BNC117/10-1074 Administration Visits 26
6.1.5 Final Visit/Early termination Visit 26
6.1.6 Unblinding 27
6.1.7 Discontinuation of 3BNC117/10-1074 infusion and/or participant withdrawal from study 27
6.2 Study Procedures **28**
6.2.1 Consent Procedure 28
6.2.2 Randomization 29

6.2.3 3BNC117 and 10-1074 Administration Procedure 29
6.2.4 Medical History and Physical Examination....30
6.2.5 Blood Collection, Storage and Shipment....30
6.2.6 HIV testing and Counseling 31
6.2.7 HIV Infection....31
6.2.8 Monitoring for cytokine release associated adverse events and treatment of cytokine release syndrome 31
6.2.9 Family Planning Counseling....32
6.2.10 Reimbursement....32
6.2.11 Safety Assessments....32

7 Investigational Products....35

7.1 Regimen....35

7.2 Study Product Formulation and Preparation 35

7.3 Dispensing and Handling of Investigational Product....35

7.4 Accountability and Disposal of Used and Unused Investigational Product 36

8 Data Analysis....36

8.1 Analysis of Safety, PK and Antiretroviral effects....36

8.2 Sample Size Considerations 37

8.3 Analyses prior to the end of the study....38

9 Data and Sample Storage 38

10 Recruitment and Advertising 40

11 Potential Benefits to Participants 40

12 Potential risks to the participant....40

12.1 Procedures to minimize risk 42

13 Data and Safety Monitoring Plan....43

13.1 Safety Monitoring Committee and Stopping Rules....43

13.2 Monitoring....45

13.3 Adverse Event Classification 45

13.4 Reporting Adverse Events....46

13.5 Reporting Unanticipated AEs....46

13.6 Clinical Laboratory Improvement Amendment/Clinical Laboratory Evaluation Program (CLIA/CLEP) 46

14 Clinical Trial Registration....46

15 Bibliography....47

List of Abbreviations

10-1074	Anti-HIV-1 bNAb targeting the V3 loop of gp120
3BNC117	Anti-HIV-1 bNAb targeting the CD4 binding site of gp120
Ab	Antibody
AE	Adverse Event/Adverse Experience
ART	Antiretroviral Therapy
ATI	Analytic Treatment Interruption
bNAbs	Broadly Neutralizing Antibodies
CD4	T-cell Surface Glycoprotein CD4
CFR	Code of Federal Regulations
cGMP	Current Good Manufacturing Practices
CONSORT	Consolidated Standards of Reporting Trials
CRF	Case Report Form
CRSO	Clinical Research Support Office
CTSA	Clinical and Translational Science Award
CCTS	Center for Clinical and Translational Science
DC	Dendritic Cell
DNA	Deoxyribonucleic Acid
DSMB	Data and Safety Monitoring Board
FDA	Food and Drug Administration
FWA	Federal-Wide Assurance
GCP	Good Clinical Practice
gp120	HIV-1 Envelope Glycoprotein 120
HIPAA	Health Insurance Portability and Accountability Act
HIV-1	Human immunodeficiency virus
hu-mice	Humanized Mice
ICF	Informed Consent Form
ICH	International Conference on Harmonization
I.M.	Intramuscularly
IND	Investigational New Drug
IRB	Institutional Review Board
I.V.	Intravenously
MTD	Maximum tolerated dose
N	Number (typically refers to participants)
NIAID	National Institute of Allergy and Infectious Diseases, NIH
NIH	National Institutes of Health
OHRP	Office for Human Research Protections
OHSR	Office for Human Subjects Research
PBMC	Peripheral Blood Mononuclear Cell
PI	Principal Investigator
RU	The Rockefeller University
RUH	The Rockefeller University Hospital
QA	Quality Assurance
QC	Quality Control
RNA	Ribonucleic Acid
SAE	Serious Adverse Event/Serious Adverse Experience
S.C.	Subcutaneously
SHIV	Chimeric Simian/Human Immunodeficiency Virus
SMC	Safety Monitoring Committee
SOP	Standard Operating Procedure
T cell	T lymphocyte

V3 loop Third Variable Loop of HIV-1 Envelope gp 120

Protocol Summary

<table>
<tr><td>Title</td><td>A phase 1 study of the safety and pharmacokinetics of the combination of the monoclonal antibodies 3BNC117 and 10-1074 in HIV-uninfected adults</td></tr>
<tr><td>Short Title</td><td>3BNC117 and 10-1074 in HIV uninfected adults</td></tr>
<tr><td>Protocol Number</td><td>YCO-899</td></tr>
<tr><td>Phase</td><td>Phase 1</td></tr>
<tr><td>Study Design</td><td>

This is a phase 1 clinical trial to evaluate the safety and pharmacokinetics of the highly neutralizing anti-HIV-1 monoclonal antibodies 3BNC117 and 10-1074, when given in combination, in HIV-uninfected individuals. This study is intended to support the development of the combination of 3BNC117 and 10-1074 mAbs for use as prophylaxis against HIV infection in healthy HIV-uninfected individuals at risk for HIV infection.

The study will be conducted under a placebo-controlled, double blind, randomized allocation of the study products. Study participants in each study group will be randomized to receive intravenous infusions of 3BNC117 and 10-1074 or placebo. 3BNC117 and 10-1074 will be administered at 2 dose levels (3 mg/kg and 10 mg/kg). These doses were selected based on the planned development of subcutaneous formulations for the prophylactic use of these products. The study will consist of 3 groups (**Figure 1**). Enrollment in Group 1 will begin first and participants will be enrolled at least one day apart. Eight participants will be enrolled in each group; 6 will be randomized receive the study drugs and 2 will be randomized to receive placebo. Participants in Group 1 will receive a single infusion of each antibody at 10mg/kg at Week 0. Enrollment in Group 2 will begin only after Day 28 safety data from all participants in Group 1, including ophthalmologic exams, are available. Enrollment in Group 3 will begin only after the Day 28 safety data from all participants in Group 2 is available. If $>$ 1 grade 3 or higher adverse events deemed probably or definitely related to the study drugs occurs in a single group, the next group will not be enrolled pending SMC review. Participants in Groups 2 and 3 will receive 3 infusions of each antibody at Weeks 0, 8, and 16, at a dose of either 3mg/kg or 10mg/kg. The antibodies will be administered sequentially via intravenous infusion. Each antibody will be administered over 60 minutes.

Figure 1. Study Design

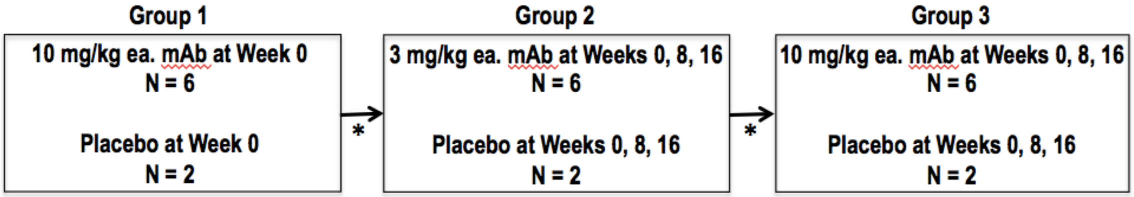

* Enrollment in subsequent group may begin following review of Day 28 safety data from all subjects in the previous group

Following product infusions, study participants will return for safety assessments at multiple time points. Blood samples will be collected for safety testing at weeks 1, 2, and 4 following each infusion, then at multiple time points until the end of study follow up.

Baseline pharmacokinetic assessments will be performed before the start of the first infusion. Peak PK sampling for 3BNC117 will occur following the completion of the 3BNC117 infusion prior to the start of the 10-1074 infusion. Peak PK sampling for 10-1074 will occur following the completion of the 10-1074 infusion. Additional PK assessments will occur at multiple time points during study follow up, as outlined in **Appendix A**. Study participants will be followed for a total of 24 weeks following the final antibody infusion.

</td></tr>
</table>

**Study Duration**	36 months
**Study Center(s)**	Single-center – The Rockefeller University
**Objectives**	The primary objective of the study is to evaluate the safety, tolerability, and pharmacokinetics of the combination of 3BNC117 and 10-1074 in HIV-uninfected, healthy participants.<br><br>The secondary objective of the study is to evaluate the frequency and magnitude of induced anti-3BNC117 and anti-10-1074 antibodies.<br><br>The exploratory objective of the study is to evaluate the serum neutralizing activity of the combination of 3BNC117 and 10-1074.
**Number of Participants**	24

**Inclusion and Exclusion Criteria**	**<u>Inclusion Criteria</u>**:<br>1. Males and females, age 18 to 65.<br>2. Amenable to HIV risk reduction counseling and agrees to maintain behavior consistent with low risk of HIV exposure.<br>3. If sexually active male or female, and participating in sexual activity that could lead to pregnancy, agrees to use two effective methods of contraception (i.e. condom with spermicide, diaphragm with spermicide, hormone-eluting IUD, hormone-based contraceptive with condom) for the duration of the study.<br><br>**<u>Exclusion Criteria</u>:**<br>1. Confirmed HIV-1 or HIV-2 infection.<br>2. History of immunodeficiency or autoimmune disease; use of systemic corticosteroids, immunosuppressive anti-cancer, or other medications considered significant by the trial physician within the last 6 months.<br>3. Any clinically significant acute or chronic medical condition (such as autoimmune diseases) that in the opinion of the investigator would preclude participation.<br>4. Within the 12 months prior to enrollment, the participant has a history of sexually transmitted infection.<br>5. Chronic Hepatitis B or Hepatitis C infection.<br>6. Laboratory abnormalities in the parameters listed:<br>a. Absolute neutrophil count ≤ 2,000;<br>b. Hemoglobin ≤ 12 gm/dL if female; ≤ 13.5 gm/dL if male;<br>c. Platelet count ≤ 125,000;<br>d. ALT ≥ 1.25 x ULN; AST ≥ 1.25 x ULN;<br>e. Alkaline phosphatase ≥ 1.5 x ULN<br>f. Total bilirubin > 1.0 x ULN;<br>g. Creatinine ≥ 1.1 x ULN;<br>7. Pregnancy or lactation.<br>8. Any vaccination within 14 days prior to infusion<br>9. Receipt of any experimental HIV vaccine in the past.<br>10. History of severe reaction to a vaccine or drug infusion or history of severe allergic reactions.<br>11. Participation in another clinical study of an investigational product currently or within past 12 weeks, or expected participation during this study.
**Study Products, Dose, Route, Regimen**	3BNC117 is a recombinant, fully human monoclonal antibody (mAb) of the IgG1κ isotype that specifically binds to the CD4 binding site (CD4bs) within HIV-1 envelope gp-120.<br><br>10-1074 is a recombinant, fully human mAb of the IgG1κ isotype that specifically binds to the base of the V3 loop within HIV envelope gp120.<br><br>Both are manufactured by Celldex Therapeutics.<br><br>In Group 1, one intravenous infusion of both 3BNC117 and 10-1074 at a dose of 10mg/kg will be administered on Week 0, via a peripheral vein. Each mAb will be administered over 60 minutes.<br><br>In Groups 2 and 3, three intravenous infusions of both 3BNC117 and 10-1074 at a dose of either 10mg/kg or 3mg/kg will be administered on Weeks 0, 8, and 16 via a peripheral vein. Each mAb will be administered over 60 minutes.

Statistical Methodology

The safety population will include all participants who receive at least one dose of study treatment. A baseline measurement and at least one laboratory, vital sign, or other safety-related measurement obtained after at least one dose of study treatment will be required for inclusion in the analysis of a specific safety parameter.

With 6 placebo participants, the 95% upper binomial confidence limit for the proportion with adverse events is 46%. For the proposed 18 participants when combining all three study treatment groups, the upper limit for the proportion with adverse events is 19%. The table below shows the upper 95% confidence limits for the binomial proportion when no event has been observed for sample sizes ranging from 6 to 20.

Sample Size	Proportion	Sample Size	Proportion	Sample Size	Proportion
6	0.46	11	0.28	16	0.21
7	0.41	12	0.26	17	0.20
8	0.37	13	0.25	18	0.19
9	0.34	14	0.23	19	0.18
10	0.31	15	0.22	20	0.17

1.1.1.1.1 The Fisher's exact test will be used to compare the proportion of adverse events in placebo and treatment groups.

Pharmacokinetic parameters will be calculated using standard non-compartmental analysis methods. Descriptive results will be presented for the pharmacokinetic parameters by dose group.

Continuous data will be summarized by descriptive statistics, including sample size, mean, standard deviation, median and range. Categorical data will be summarized by the number and percentage of participants with an outcome.

1. Key Roles

1.1 Study Site and associated Institutions

The Rockefeller University Hospital, New York NY
1230 York Ave.
New York, NY 10065
Contact Person: Yehuda Cohen, MD
Phone Number: (212) 327-7393
Fax Number: (212) 327-7234
E-mail: ycohen@mail.rockefeller.edu

Clinical Laboratories:
- Memorial Sloan Kettering Cancer Center
1275 York Avenue
NY, NY 10065

- LabCorp
330 W 58th St
New York, NY, 10019

Funder: Bill and Melinda Gates Foundation

Data Management:
Emmes
401 N. Washington St. Suite 700
Rockville, MD 20850

Independent Safety Monitoring:
International AIDS Vaccine Initiative (IAVI)
125 Broad Street, 9th Floor
New York, NY 10004

1.2 Study Personnel

1.1.2 Principal Investigator

Yehuda Cohen, MD

1.1.3 Protocol Staff Personnel – Co-investigators

Marina Caskey, MD
Michel Nussenzweig, MD, PhD
Julio Lorenzi, PhD
Arlene Hurley, ANP
Irina Shimeliovich, MD PhD
Maggi Pack, PhD
Allison Butler, FNP

Katrina Millard, ANP

1.1.4 Consultants

Michael Seaman, PhD
Beth Israel Deaconess Medical Center
330 Brookline Ave
E/CLS-1001
Boston, MA 02215

Szilard Kiss, MD
Weill Cornell Ophthalmology
1305 York Ave
New York, NY 10021

Ype de Jong, MD PhD
Weill Cornell Medical Center
1305 York Ave
New York, NY 10021

2 Lay Summary

Over the past few years, a significant number of very broad and potent neutralizing antibodies against HIV have been isolated. These antibodies have the potential to protect people against infection with HIV. They have been shown in studies to protect humanized mice and macaque monkeys from infection, and the presence of anti-HIV antibodies was the only positive correlate of protection in an HIV vaccine efficacy trial (RV144 trial). 3BNC117 and 10-1074 are highly neutralizing anti-HIV antibodies that were isolated in the laboratory of Dr. Nussenzweig at Rockefeller University. Both 3BNC117 and 10-1074 have been shown to be safe and well tolerated in humans when given alone. The combination of these antibodies increases their breadth of coverage and could provide protection against a greater number of HIV strains. The aim of this protocol is to evaluate the safety, tolerability, and pharmacokinetics of the combination of 3BNC117 and 10-1074 in HIV-uninfected individuals.

3 Objectives and Rationale

3.1 Overview

3.1.1 Background

It is estimated that over 36 million people were living with HIV in 2014 (UNAIDS). Despite intense research for over 30 years, an effective vaccine against HIV-1 remains elusive, and there are no vaccine candidates approaching licensure. Antibodies are key components of all effective vaccine responses and are believed to be essential for protection

against HIV-1 infection (Plotkin 2010).

Two to three years after infection, a fraction of HIV-infected individuals (10 – 30%) mount a serologic response that can neutralize a broad spectrum of HIV-1 isolates (Simek, Rida et al. 2009). Recent evidence supports the idea that broadly neutralizing antibodies (bNAbs) might play an important role in both HIV prevention and therapy. Moreover, understanding how highly potent neutralizing antibodies are elicited during chronic infection and determining their *in vivo* activity are important for guiding future vaccine design, as vaccines that induce such antibodies are likely to interfere with transmission and confer protection (Klein, Mouquet et al. 2013, West, Scharf et al. 2014).

Although broadly neutralizing antibodies that arise during HIV infection fail to resolve established infection, the selection of resistant strains indicates that bNAbs exert selective pressure on the virus. Even with a prior generation of less broad and potent neutralizing antibodies, several groups had shown that macaque simian-human immunodeficiency virus (SHIV) infection could be prevented by passive transfer of such antibodies (Mascola, Lewis et al. 1999, Shibata, Igarashi et al. 1999). High antibody concentrations were required to prevent intravenous infection, but the antibodies used in these early studies were far less potent than currently available antibodies. Subsequently it was demonstrated that lower doses of neutralizing antibodies could achieve protection from multiple low dose mucosal challenges (Hessell, Poignard et al. 2009). Protection experiments with the more potent, recently discovered, antibodies prevented infection when antibodies were administered at concentrations as low as 0.2 - 5mg/kg (Moldt, Rakasz et al. 2012, Shingai, Nishimura et al. 2013, Shingai, Donau et al. 2014). In addition, the presence of anti-HIV-1 IgG antibodies was the only significant, positive correlate of protection in the RV144 vaccine efficacy clinical trial (Haynes, Gilbert et al. 2012).

Current HIV prevention strategies include education, use of condoms, voluntary male circumcision, pre-exposure prophylaxis (PrEP) and the treatment of HIV-infected partners with antiretroviral therapy (ART). These and other available strategies have limitations in efficacy, access, cost and compliance. The use of antiretroviral agents by HIV-uninfected persons before potential sexual exposure to HIV-infected partners, known as pre-exposure prophylaxis, is a new approach to HIV prevention. Phase III studies have shown that the combination of tenofovir and emtricitabine (TDF-FTC) can prevent HIV acquisition in selected high-risk populations (Grant, Lama et al. 2010, Baeten, Donnell et al. 2012, Thigpen, Kebaabetswe et al. 2012). However, an important challenge to the success of such strategy is that its efficacy is highly dependent on adherence to a daily oral drug regimen (Cohen and Baden 2012, Marrazzo, Ramjee et al. 2015) Therefore, it is clear that alternative regimens that are safe and have effective antiretroviral activity, but that allow for less frequent dosing are needed, especially when targeting populations known to have poor adherence rates. Broadly neutralizing antibodies are promising alternatives as they are likely to be safe and well tolerated, penetrate mucosal tissues (sites of viral entry) and to persist at neutralizing levels for extended periods of time.

3.1.2 Clinical Safety of other Monoclonal Antibodies against Infectious Pathogens

There are over 30 FDA-approved mAbs for treatment or prevention of cancer, autoimmune diseases, infectious diseases, and other conditions. While each mAb product has unique safety issues related to its mechanism of action, the major safety concern related to mAbs in general is infusion/hypersensitivity reactions, which are more common for mAbs that contain murine elements. 3BNC117 and 10-1074 are fully human recombinant forms of naturally existing human mAbs.

Passive administration of antibodies is successfully used to prevent or treat viral diseases and several monoclonal antibodies are being developed for use in either prevention or treatment of infectious diseases. For example, CMV immunoglobulin is used for the prevention of transplant-associated infection, while human rabies immunoglobulin is used in conjunction with a vaccine after suspected or proven exposure to rabies. Palivizumab, a humanized monoclonal antibody (IgG) directed against the fusion protein of respiratory syncytial virus (RSV), is the first monoclonal antibody approved for clinical use against an infectious pathogen and it is indicated for the prevention of serious lower respiratory tract disease caused by RSV in children at high risk of RSV disease. Pavilizumab is generally safe and well tolerated. Rare cases of severe hypersensitivity reactions (<1 per 100,000 recipients) have been described after an initial dose, as well as after re-exposure. Several other monoclonal antibodies are being developed for use in either prevention or treatment of other viral illnesses.

Passive administration of anti-HIV-1 antibodies has also been evaluated in humans. HIV Immune Globulin (HIVIG) was evaluated in clinical studies in the 1990s before the advent of highly effective ART. HIVIG was also evaluated in HIV-infected pregnant females and their newborns in a phase III trial to assess whether HIVIG plus single dose nevirapine given to mothers and infants would provide additional benefit over single dose nevirapine alone for prevention of peripartum HIV transmission. While there was no demonstrable difference in treatment efficacy between nevirapine with and without HIVIG, the study showed that that there were no significant differences in mortality or serious AEs between the two arms of the trial (Onyango-Makumbi, Omer et al. 2011).

Several monoclonal antibodies that target HIV-1 have been evaluated in clinical studies, either alone or in combination. For example, 2F5 and 4E10 are IgG1 (kappa) monoclonal antibodies that target the membrane-proximal ectodomain of gp41, while 2G12 binds to a carbohydrate moiety on the silent face of gp120. These neutralizing antibodies were evaluated in combination in HIV-infected individuals (Armbruster, Stiegler et al. 2004). The antibodies were administered intravenously at 0.5 to 1 g doses; 4 to 8 weekly infusions were given. The antibodies were safe and well tolerated and no clinical or laboratory abnormalities were observed throughout the studies. A low-level antibody response against 2G12 was found in two participants.

Two other studies included HIV-infected participants on combination ART and plasma viral levels < 50 copies/ml (Trkola, Kuster et al. 2005), n = 14; (Mehandru, Vcelar et al. 2007), n =10. The antibodies were administered intravenously at doses ranging from 1 to 2 g for each antibody; 13-16 weekly antibody infusions were given. ART was interrupted following 1 or 4 antibody infusions. Antibody infusions were well tolerated in most

participants; mild and transient side effects were reported only occasionally. No serious adverse events were recorded. In both studies, the use of mAbs was safe and generally delayed, but did not prevent viral rebound. The emergence of resistance to 2G12, however, demonstrated that the antibody exerted selective pressure on the circulating viral strains. It is important to note that the antibodies used in these studies have far lower potency and breadth than the more recently isolated neutralizing antibodies, such as 3BNC117 and 10-1074.

VRC01, another broadly neutralizing antibody targeting HIV-1 envelope CD4 binding site has been administered to both HIV-uninfected and HIV-infected participants at doses ranging from 1 to 40 mg/kg, without significant AEs (Ledgerwood, Coates et al. 2015, Lynch, Boritz et al. 2015). In HIV-infected individuals, its half-life was 12 days when administered intravenously and 11 days when administered subcutaneously. In HIV-uninfected individuals, the half-life was 15 days. Eight viremic individuals received a single infusion of VRC01 at 40 mg/kg. After infusion with VRC01, plasma viremia declined by 1.1 to 1.8 $\log_{10}$ in six of the eight participants, and in all participants viremia returned to baseline levels within 56 days after infusion.

3.2 Preclinical Studies with 3BNC117 and 10-1074

3.2.2 In Vitro Neutralizing Activity

3BNC117 is a broadly neutralizing and highly potent anti-HIV-1 antibody. 3BNC117 was initially cloned from a single B cell isolated from an individual infected with clade B HIV-1. 3BNC117 targets the CD4 binding site (CD4bs) within HIV-1 envelope gp-120 (Scheid, Mouquet et al. 2011). 10-1074 was cloned from an African donor (Patient 10 (Simek, Rida et al. 2009), infected with clade A HIV-1. 10-1074 was identified by a similar method used to isolate and clone 3BNC117, and targets the base of the V3 loop within HIV-1 envelope gp120 (Mouquet, Klein et al. 2011; Mouquet, Warncke et al. 2012). Both antibodies were chosen for clinical development for their neutralizing breadth and potency, and their antiretroviral activity when tested in humanized mice and non-human primates models (Klein, Halper-Stromberg et al. 2012; Horwitz, Halper-Stromberg et al. 2013; Shingai, Nishimura et al. 2013; Shingai et al 2014).

In vitro neutralizing activities of 3BNC117 and 10-1074 have been tested by TZM.bl assays against large viral panels. 3BNC117 showed neutralizing activity against 82.5% of 399 tested viral strains from different clades, with an average IC_{50} of 0.15 μg/ml among sensitive strains (defined as IC_{50} < 10 μg/ml). 10-1074 neutralized 61.7% of 295 viral strains tested, with an average IC_{50} of 0.06 μg/ml among sensitive strains.

In vitro neutralization data demonstrate that the combination of 3BNC117 and 10-1074 provides broader coverage of viral strains from diverse clades (**Figure 2**). The 3BNC117 and 10-1074 combination has neutralizing activity against 96% of 125 viruses from multiple clades (Kong at al 2015), with average IC_{50} of 0.04 μg/ml and IC_{80} 0.15 μg/ml. The 3BNC117 and 10-1074 combination neutralizing titers against a panel of 200 clade C viruses were predicted using the Bliss-Hill model (Wagh *et al.*, 2016). Against this panel,

the combination of mAbs covers 87% of viral strains with IC_{50} of 0.05 μg/ml and IC_{80} of 0.16 μg/ml.

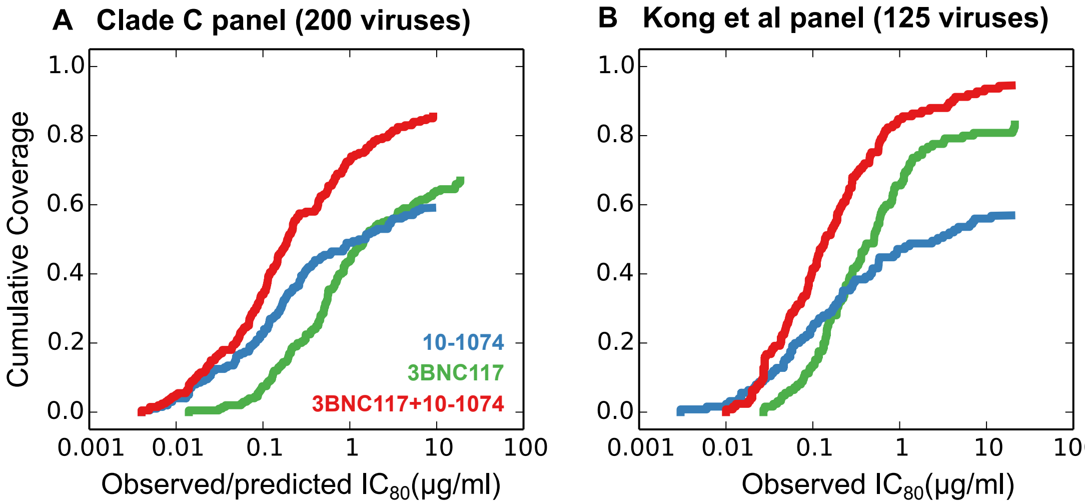

Figure 2. (A) Potency versus breadth curves are shown for 3BNC117, 10-1074 and their combination (3BNC117 + 10-1074) for a clade C panel (n=200) using IC_{80} titers. The single bNAb IC_{80} titers shown are observed, while the combination IC_{80} titers were predicted using the Bliss-Hill model (Wagh *et al.*, 2016). **(B)** Same as (**A**) except all IC_{80} titers are shown for the Kong et al panel of 125 global viruses and all IC_{80} titers shown are experimental.

3.2.3 In Vivo Activity in Humanized Mice and Non-human Primates

3BNC117 and 10-1074 were evaluated in humanized mice and non-human primate models during protection and treatment experiments.

A single infusion of 3BNC117 or 10-1074, at 5 or 10 mg/kg, protected rhesus macaques from high-dose intrarectal challenge with two tier 2 SHIVs, (Shingai, Nishimura et al. 2013, Shingai, Donau et al. 2014). 3BNC117 and 10-1074 were also evaluated in multiple low-dose weekly intrarectal challenge experiments with SHIV-AD8. Without pre-treatment, an average of 3.7 challenges were required for HIV-1 acquisition. Following a single 20 mg/kg dose of 3BNC117, infection occurred after an average of 13.5 challenges, and the time to acquisition was 7 to 20 weeks. After 10-1074, infection occurred after 12.6 challenges, and the time to acquisition was 6 to 23 weeks, as opposed to 2 to 6 weeks without treatment (Martin et al, unpublished data). These data suggest that 3BNC117 and/or 10-1074 infusions, administered every 8 to 12 weeks, are likely to protect against SHIV or HIV-1 infection.

Passive administration of 3BNC117 or 10-1074 resulted in transient decline of approximately 1 log_{10} in plasma HIV-1 RNA levels in humanized mice infected with HIV-1_{YU2}. In humanized mice, viremia rebounded after 1-2 weeks of 3BNC117 or 10-1074 monotherapy and was associated with mutations in residues that map to the mAb target site, demonstrating selective pressure on circulating viruses (Klein, Halper-Stromberg et al. 2012). In contrast to monotherapy, administration of 3BNC117, 10-1074 and PG9

controlled established infection in humanized mice for prolonged periods of time. Combination antibody therapy resembled ART, in that escape required the improbable appearance of multiple simultaneous mutations (Klein, Halper-Stromberg et al. 2012, Horwitz, Halper-Stromberg et al. 2013).

While 3BNC117 or 10-1074 monotherapy did not control HIV-1 infection in untreated humanized mice, either antibody alone controlled infection when plasma HIV-1 RNA levels were initially suppressed by ART. While humanized mice that received ART normally rebounded immediately after the drugs were terminated, continuing 3BNC117 or 10-1074 was sufficient to maintain control after ART interruption in 50-86% of the hu-mice, for as long as antibody concentrations remained therapeutic (Horwitz, Halper-Stromberg et al. 2013). Viruses that escaped 3BNC117 carried resistance mutations in the CD4 binding site at positions $YU2^{(279\text{-}281)}$ or $YU2^{(458/459)}$, and viruses that escaped 10-1074 carried mutations in the V3 loop at positions $YU2^{(325,\ 332,\ 334)}$. In contrast, viruses that emerged after immunotherapy was terminated did not contain antibody resistance mutations and remained sensitive to neutralization by the antibodies.

In non-human primates, chronically infected with SHIV-AD8, a single dose of 3BNC117 and 10-1074 (10 mg/kg of each mAb) rapidly suppressed viremia by an average of 2 $\log_{10}$ copies/ml (**Figure 3**). Viremia suppression was maintained until a threshold plasma mAb concentration of approximately 5 µg/ml was reached. Resistance to 3BCN117 did not emerge, but 10-1074 escape variants emerged after 1 animal received a second antibody dose (Shingai M et al. 2013).

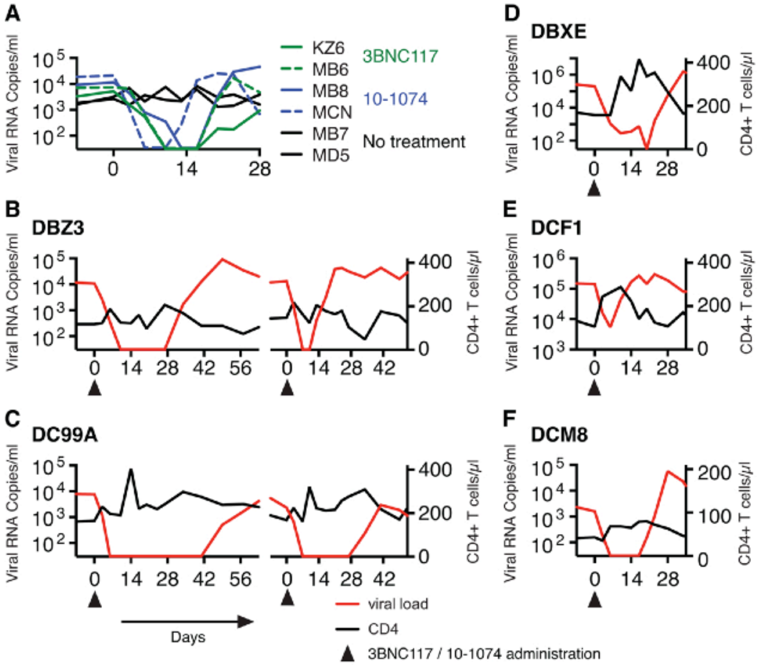

Figure 3. (**A**) Plasma viral loads in post-acute set-point SHIV infected rhesus macaques with/without single mAb treatment. (**B-F**) Plasma viral loads (red line) and total CD4+ T cell numbers (black line) in chronically SHIV infected rhesus macaques [DBZ3 (**B**), DC99A (**C**), DBXE (**D**), DCF1 (**E**) and DCM8 (**F**)] following combination 3BNC117 and10-1074 treatment.

3.3 3BNC117

3.3.2 3BNC117 Drug Product

3BNC117 was manufactured for clinical use under cGMP by Celldex Therapeutics. The manufacture of the recombinant human monoclonal 3BNC117 was carried out by *in vitro* serum-free CHO cell culture. 3BNC117 was manufactured as a sterile solution intended for parenteral use, in compliance with Good Manufacturing Practices (GMP). No animal-derived raw materials were used during the cell culture, purification, and formulation of the drug substance. The drug substance was manufactured in a dedicated suite utilizing single-use equipment (e.g., WAVE bioreactor) to minimize potential for product cross contamination. A low pH step and a nanofiltration step were used for virus inactivation and reduction. Viral clearance studies used the model viruses PPV and A-MuLV. Testing for adventitious agents was performed in accordance to FDA Points to Consider in the Manufacture and Testing of Monoclonal Antibody Products for Human Use (1997).

An ongoing drug product stability-testing program monitors the quality of 3BNC117 over the duration of the clinical dosing period. Stability is evaluated in real time at the recommended storage conditions of 5 ± 3°C as well as at accelerated temperature conditions of 25 ± 2°C / 60 ± 5% RH.

3.3.3 Preclinical Toxicity Studies with 3BNC117

[redacted]

[redacted]

[redacted]

3.3.4 Clinical Experience with 3BNC117

3BNC117 was evaluated in a phase 1 study in both HIV-uninfected and HIV-infected participants (protocol MCA-835) and is currently being tested in two ongoing exploratory phase 2a studies in HIV-infected participants (protocols MCA-867 and MCA-866).

In protocol MCA-835, study participants were administered one or two intravenous infusions of 3BNC117 at increasing dose levels (1 mg/kg, 3 mg/kg, 10 mg/kg or 30 mg/kg), and were followed for 24 weeks after last infusion. In total, 55 participants (22 HIV-uninfected, 17 viremic HIV-infected and 16 ART-treated HIV-infected individuals) were enrolled in the study. Five HIV-uninfected individuals received two infusions of 3BNC117 at 30 mg/kg, 12 weeks apart. Twenty-two participants (3 HIV-uninfected and 19 HIV-infected) were administered one dose of 30 mg/kg.

Overall, 3BNC117 was generally safe and well-tolerated, mild transient myalgia, fatigue and headache were the most commonly reported adverse events (AEs). Some participants reported ophthalmic complaints, but a causal relationship with 3BNC117 was not established. No SAEs or grade 3/4 AEs deemed related to 3BNC117 occurred. A safety data summary is included in the Investigator's Brochure (IB).

Preliminary PK data show that 3BNC117's half-life is 17.6 days in HIV-uninfected and 9.6 days in viremic HIV-infected individuals. 3BNC117 decay rates following first and second infusions appear to be similar.

When administered at 30 mg/kg, 3BNC117 induced rapid decreases in plasma HIV-1 RNA levels that varied between individuals from 0.8 to 2.5 $\log_{10}$ copies/ml. The median time to reach the nadir in viremia was 7 days, and the mean drop in VL was 1.48 $\log_{10}$ copies/ml at nadir. Emergence of resistant viral strains was variable, with some individuals remaining sensitive to 3BNC117 for a period of 28 days after infusion (Caskey, Klein et al. 2015).

In protocol MCA-867, HIV-infected individuals on suppressive ART are administered two 30 mg/kg intravenous infusions of 3BNC117 at weeks 0 and 3 (group A), or four infusions at weeks 0, 2, 4 and 6 (group B). ART is discontinued 2 days after the first 3BNC117 infusion. Participants are followed weekly and ART is resumed if viral rebound occurs or CD4+ T cell counts decline to < 350 cells/mm^3. As of 12 January 2016, 11 individuals have been enrolled. 3BNC117 infusions have been well tolerated, with few mild and moderate AEs reported to date.

Among the first 7 enrolled participants who received two 3BNC117 infusions (group A), viral rebound occurred 4 to 9 weeks after ART was discontinued, with an average time to rebound of 6.28 weeks. 3BNC117 levels at the time of viral rebound ranged from 18.3 – 138.3 µg/ml, with an average of 76.3 µg/ml (**Figure 4**). Three individuals have enrolled in group B, and 2 of them have received 3 3BNC117 infusions to date. One participant enrolled in group B experienced viral rebound at week 3 of discontinuing ART, prior to receiving the 3rd dose of 3BNC117.

<u>In protocol MCA-866</u>, HIV-infected participants on ART will be administered four 3BNC117 infusions, at weeks 0, 12, 24 and 27. ART will be discontinued at week 24, and participants will be followed weekly to monitor plasma HIV-1 RNA levels. ART will be reinitiated according to the same criteria as in protocol MCA-867 (see above). To date, 3 participants have enrolled and received the first 3BNC117 infusion. They remain on ART with continued suppression of viremia.

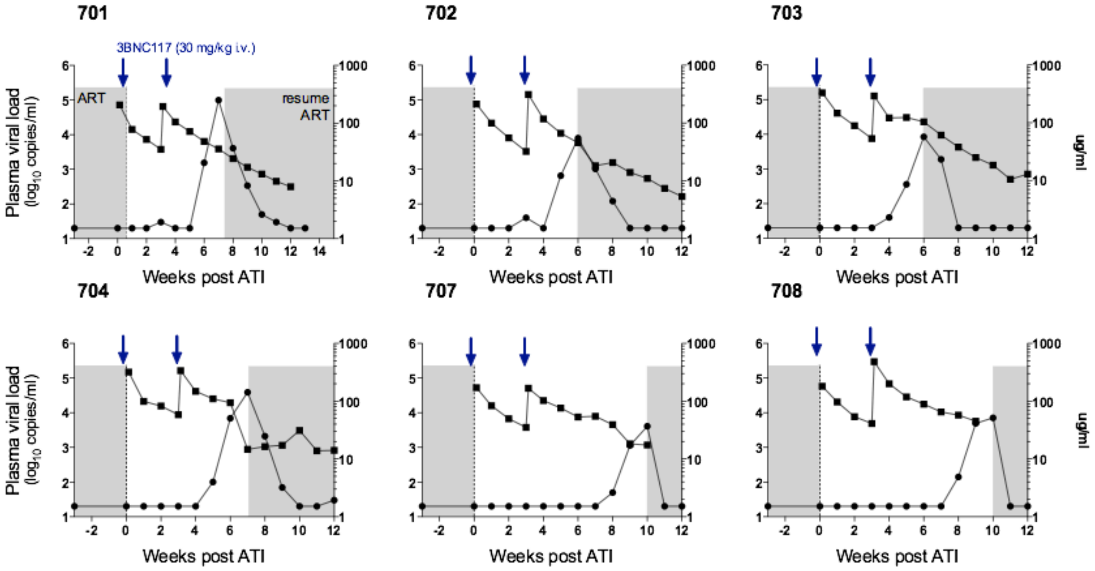

Figure 4. Plasma HIV-1 RNA Levels and 3BNC117 serum levels in participants Enrolled in protocol MCA-867, group A. *Participants MCA867-705 and -706 viral loads were not fully suppressed at day 0 and are not displayed in this graph.

3.4 10-1074

3.4.2 10-1074 Drug Product

10-1074 was manufactured by Celldex therapeutics as a sterile solution intended for parenteral use, in compliance with Good Manufacturing Practices (GMP). The manufacture of the recombinant human monoclonal 10-1074 was carried out by *in vitro* serum-free CHO cell culture, in a similar manner to the GMP manufacture of 3BNC117.

A low pH step and a nanofiltration step were used for virus inactivation and reduction. Viral clearance studies used the model viruses PPV and A-MuLV. Testing for adventitious agents was performed in accordance to FDA Points to Consider in the Manufacture and Testing of Monoclonal Antibody Products for Human Use (1997). The clinical trial product was formulated as a single-use sterile solution intended for parenteral use.

Similarly to 3BNC117, a drug product stability-testing program is established to monitor the quality of 10-1074 over the duration of the clinical dosing period. Stability is evaluated in real time at the recommended storage conditions of 5 ± 3°C as well as at accelerated temperature conditions of 25 ± 2°C / 60 ± 5% RH.

3.4.3 Preclinical Toxicity Studies with 10-1074

[redacted]

[redacted]

- [redacted]
- [redacted]
- [redacted]
- [redacted]
- [redacted]

[redacted]

[redacted]

[redacted]

[redacted]

[redacted]

[redacted]

3.4.4 Clinical Experience with 10-1074

10-1074 is being evaluated in a phase 1 study in both HIV-uninfected and HIV-infected participants (protocol MCA-885). Study participants are administered one intravenous infusion of 10-1074 at increasing dose levels (3 mg/kg, 10 mg/kg or 30 mg/kg), and are followed for 24 weeks after infusion.

As of 12 January 2016, 23 participants (12 HIV-uninfected, 8 viremic HIV-infected and 3 ART-treated HIV-infected individuals) have enrolled in the study. Eleven participants (6 HIV-uninfected and 5 HIV-infected) were administered one dose of 30 mg/kg. 10-1074 has been generally safe and well tolerated at all doses tested. Most common reported adverse event reported to data was fatigue.

Preliminary pharmacokinetics data show that 10-1074's half-life in HIV-uninfected individuals is approximately 15.5 days. Similarly to 3BNC117, 10-1074 has been generally safe and well-tolerated. When administered at 10 mg/kg, 10-1074 reduced plasma viremia by an average of 1.38 $\log_{10}$ copies/ml (n = 3, range 1 – 1.56 $\log_{10}$ copies/ml). At 30 mg/kg, 10-1074 led to similar decline in plasma HIV-1 RNA in the 4 out of 5 participants enrolled to date (n = 4, average 1.37 $\log_{10}$, range 1 – 1.7 $\log_{10}$ copies/ml). One participant dosed at 30 mg/kg experienced a 0.4 $\log_{10}$ decline in viremia. The baseline sensitivity of this participant's plasma viruses to 10-1074 is not yet known. The study remains open to enrollment, and safety, PK and antiretroviral activity data are being collected.

3.5 Objectives

Primary Objectives:
- To evaluate the safety and tolerability of a single intravenous infusion of the combination of 3BNC117 and 10-1074 at 10mg/kg in HIV-uninfected participants.
- To evaluate the safety and tolerability of three intravenous infusions of the combination of 3BNC117 and 10-1074, administered at 3 mg/kg and 10 mg/kg to HIV-uninfected participants.
- To determine the pharmacokinetic profile of intravenous administration of the combination of 3BNC117 and 10-1074 in HIV-uninfected participants.

Secondary Objective:
- To assess the frequency and magnitude of induced anti-3BNC117 and anti-10-1074 antibodies.

Exploratory Objective:
- To evaluate the serum neutralizing activity of the combination of 3BNC117 and 10-1074.

3.6 Outcomes

Primary Outcomes:

- The rate of signs, symptoms and laboratory abnormalities, in addition to local and systemic reactogenicity adverse events 1 week after the combination of 3BNC117 and 10-1074 infusion in all study groups.
- The pharmacokinetic profile of 3BNC117 and 10-1074: elimination half-life ($t_{1/2}$), clearance (CL/F), volume of distribution (Vz/F), AUC and decay curve in all study groups.

Secondary Outcomes:

- Frequency and levels of induced anti-3BNC117 and anti-10-1074 antibodies at 8 weeks after each infusion in all study groups.
- The rate of signs, symptoms and laboratory abnormalities that occur during study follow up after 3BNC117 and 10-1074 infusions in all study groups.

Exploratory Outcome:

- Neutralization activity of serum from study participants against a panel of viruses as measured by the TZM.bl neutralization assay.

4 Study Design

This is a phase 1 clinical trial to evaluate the safety and pharmacokinetics of the highly neutralizing anti-HIV-1 monoclonal antibodies 3BNC117 and 10-1074, when given in combination, in HIV-uninfected individuals. This study is intended to support the development of the combination of 3BNC117 and 10-1074 mAbs for use as prophylaxis against HIV infection in healthy HIV-uninfected individuals at risk for HIV infection.

The study will be conducted under a placebo-controlled, double blind, randomized allocation of the study products. Study participants in each study group will be randomized to receive intravenous infusions of 3BNC117 and 10-1074 or placebo. 3BNC117 and 10-1074 will be administered at 2 dose levels (3 mg/kg and 10 mg/kg). These doses were selected based on the planned development of subcutaneous formulations for the prophylactic use of these products. The study will consist of 3 groups (**Figure 1**). Enrollment in Group 1 will begin first and participants will be enrolled at least one day apart. Eight participants will be enrolled in each group; 6 will be randomized receive the study drugs and 2 will be randomized to receive placebo. Participants in Group 1 will receive a single infusion of each antibody at 10mg/kg at Week 0. Enrollment in Group 2 will begin only after Day 28 safety data from all participants in Group 1, including ophthalmologic exams, are available. Enrollment in Group 3 will begin only after the Day 28 safety data from all participants in Group 2 is available. If > 1 grade 3 or higher adverse events deemed probably or definitely related to the study drugs occurs in a single group, the next group will not be enrolled pending SMC review. Participants in Groups 2 and 3 will receive 3 infusions of each antibody at

Weeks 0, 8, and 16, at a dose of either 3mg/kg or 10mg/kg. The antibodies will be administered sequentially via intravenous infusion. Each antibody will be administered over 60 minutes.

Figure 1. Study Design

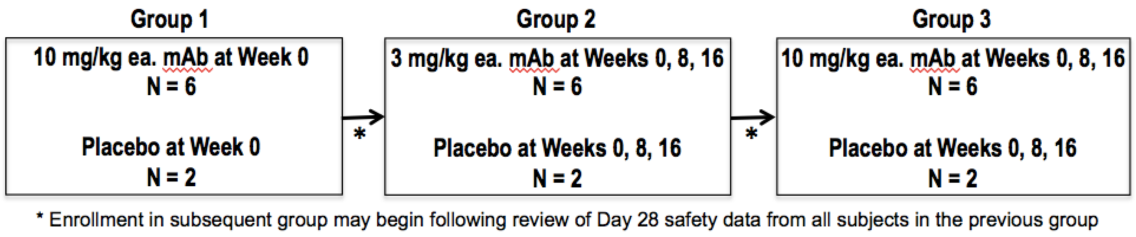

Following product infusions, study participants will return for safety assessments at multiple time points. Blood samples will be collected for safety testing at weeks 1, 2, and 4 following each infusion, then at multiple time points until the end of study follow up.

Baseline pharmacokinetic assessments will be performed before the start of the first infusion. Peak PK sampling for 3BNC117 will occur following the completion of the 3BNC117 infusion prior to the start of the 10-1074 infusion. Peak PK sampling for 10-1074 will occur following the completion of the 10-1074 infusion. Additional PK assessments will occur at multiple time points during study follow up, as outlined in **Appendix A**. Study participants will be followed for a total of 24 weeks following the final antibody infusion.

5 Study Population

5.1 Inclusion Criteria

1. Males and females, age 18 to 65.
2. Amenable to HIV risk reduction counseling and agrees to maintain behavior consistent with low risk of HIV exposure.
3. If sexually active male or female, and participating in sexual activity that could lead to pregnancy, agrees to use two effective methods of contraception (i.e. condom with spermicide, diaphragm with spermicide, hormone-eluting IUD, hormone-based contraceptive with condom) for the duration of the study.

5.2 Exclusion Criteria

1. Confirmed HIV-1 or HIV-2 infection.
2. History of immunodeficiency or autoimmune disease; use of systemic corticosteroids, immunosuppressive anti-cancer, or other medications considered significant by the trial physician within the last 6 months.
3. Any clinically significant acute or chronic medical condition (such as autoimmune diseases) that in the opinion of the investigator would preclude participation.

4. Within the 12 months prior to enrollment, the participant has a history of sexually transmitted infection.
5. Chronic Hepatitis B or Hepatitis C infection.
6. Laboratory abnormalities in the parameters listed:
    a. Absolute neutrophil count ≤ 2,000;
    b. Hemoglobin ≤ 12 gm/dL if female; ≤ 13.5 gm/dL if male;
    c. Platelet count ≤ 125,000;
    d. ALT ≥ 1.25 x ULN; AST ≥ 1.25 x ULN;
    e. Alkaline phosphatase ≥ 1.5 x ULN
    f. Total bilirubin > 1.0 x ULN;
    g. Creatinine ≥ 1.1 x ULN;
7. Pregnancy or lactation.
8. Any vaccination within 14 days prior to infusion
9. Receipt of any experimental HIV vaccine in the past.
10. History of severe reaction to a vaccine or drug infusion or history of severe allergic reactions.
11. Participation in another clinical study of an investigational product currently or within past 12 weeks, or expected participation during this study.

6 Methods and Procedures

6.1 Screening Procedure and Study Visits

The Time of Events Schedule (for Groups 1, 2, and 3) summarizes the frequency and timing of various study assessments. See **Appendix A**.

6.1.1 Pre-Screening Questionnaire

Potential participants will first undergo pre-screening by telephone to assess medical history, preliminary HIV risk assessment, and qualification for the study. Potential participants will have the opportunity to discuss the study and ask questions of the study recruiter at this time. Those who are eligible and interested in participation will attend a screening visit at the Rockefeller University Hospital (RUH) Outpatient Clinic.

6.1.2 Screening Visit

<u>Initial Screening Visit:</u>
Study personnel will answer any questions about the study. Written informed consent will be obtained prior to conducting any study procedures. To insure informed consent, the principal investigator or designee will discuss the following processes individually with each potential participant:

1. HIV-test counseling;
2. Risk-reduction counseling including safe-sex and pregnancy avoidance counseling;
3. That sexually active males and females, participating in sexual activity that could lead to pregnancy, should use two reliable forms of contraception for the duration of the trial.

If the potential participant consents to participate, site personnel will:

- Perform detailed HIV risk assessment;
- Perform complete medical history (including review of concomitant medication);
- Perform a general physical examination including height, weight, vital signs (pulse, respiratory rate, blood pressure and temperature), examination of skin, respiratory, cardiovascular and abdominal systems;
- A point of care hemoglobin test may be performed to expedite screening;
- Collect blood and urine specimens for all tests as indicated in the Time of Events Schedule (**Appendix A**);
- Perform a pregnancy test for all female participants.
- Participants that meet eligibility criteria will have an ophthalmologic assessment (including slit lamp examination) at no cost to the participant

If the initial screening visit occurs more than 49 days prior to date of the first mAb infusion, then laboratory tests (blood and urine specimens) for the screening visit must be repeated. The most recent set of results will be used.

6.1.3 3BNC117/10-1074 Infusion Visit

Prior to 3BNC117/10-1074 infusions, site personnel will:

- Review the informed consent form administered at screening visit with the participant;
- Answer any questions about the study;
- Review interim medical history (including concomitant medications);
- Review safety laboratory data;
- Perform a physical examination including weight, vital signs (pulse, respiratory rate, blood pressure and temperature) and any further examination indicated by history or observation;
- Collect blood and urine specimens for all tests as indicated in the Time of Events Schedule (**Appendix A**);
- Perform pregnancy counseling;
- Perform a pregnancy test for all female participants and obtain results prior to drug infusion;
- Perform baseline assessment and record any systemic symptoms;
- 3BNC117 and 10-1074 will be prepared for administration according to the RUH Pharmacy Standard Operating Procedures;
- 3BNC117 will be administered via a peripheral vein over 60 minutes. The IV line will be flushed with normal saline after infusion is completed;
- 10-1074 mAb will be administered after 3BNC117 infusion is completed and the line is flushed with normal saline. It will be infused via a peripheral vein over 60 minutes. The IV line will be flushed with normal saline after infusion is completed;
- Study participants in Group 1 will be observed for 4 hours after the 10-1074 infusion is completed. Presence or absence of adverse events will be recorded at 1 and 4 hours post infusion; study participants in Groups 2 and 3 will be observed for

1 hour. Presence or absence of adverse events will be recorded at 1 hour post infusion.

- Vital signs (pulse, respiratory rate, blood pressure and temperature) will be monitored at end of 3BNC117 infusion, at the end of 10-1074 infusion, 1hr (+/- 5 minutes) and 4 hrs (+/-10 min; group 1 only) post 10-1074 infusion.
- If participants develop acute infusion reaction during 3BNC117 or 10-1074 administration, the infusion will be discontinued and will not be reinitiated. If the acute infusion reaction occurs during 3BNC117 infusion, 10-1074 will not be infused. Rescue medications, including acetaminophen, diphenhydramine and glucocorticoids will be available in the RUH inpatient unit for use if clinically indicated.

6.1.4 Post-3BNC117/10-1074 Administration Visits

Participants will be followed for 24 weeks after the last 3BNC117 and 10-1074 infusions.

At these follow up visits the following will be conducted:

- Review of interim medical history and use of concomitant medications;
- If symptoms are present, perform a symptom-directed physical examination;
- Local and systemic reactogenicity adverse events, as well as other adverse events, will be assessed;
- Pregnancy and safe sex counseling;
- Vital Signs;
- Collect blood and urine specimens for all tests as indicated in the Time of Events Schedule (**Appendix A**);
- In case of adverse event(s), the participant will be assessed and followed up by the clinical team. Supplemental visit(s) for further investigation can be planned at the discretion of the principal investigator or designee. Supplemental visit(s) may be recommended if clinically indicated or to clarify observations.
- Any participant that develops signs or symptoms of ocular disease will be promptly referred to an ophthalmologist for diagnosis and management
- Participants in Group 1 will have scheduled ophthalmologic exams at Day 28; participants in Groups 2 and 3 will have a scheduled ophthalmologic exam at Week 20 (28 days following the third dose).

Specific procedures to be performed at each follow up visit for Groups 1, 2, and 3 are illustrated in the Time of Events Schedules (**Appendix A**).

Any abnormalities (adverse events) attributed to study drug, including laboratory abnormalities, should be subsequently followed until the event or its sequelae resolve or stabilize.

6.1.5 Final Visit/Early termination Visit

Assessments will be undertaken according to the Time of Events Schedule (**Appendix A**).

6.1.6 Unblinding

After all participants have completed the final study visit, participants will be informed as to whether they received the study product or placebo. This will be performed by phone.

6.1.7 Discontinuation of 3BNC117/10-1074 infusion and/or participant withdrawal from study

6.1.7.1 Discontinuation of 3BNC117/10-1074 infusions

Antibody infusion will be discontinued for any of the following reasons:

1. Any immediate hypersensitivity reaction (such as urticarial rash; bronchospasm; laryngeal edema; anaphylaxis; syncope).
2. Life threatening medical event during 3BNC117 or 10-1074 infusion.

6.1.7.2 Discontinuation from subsequent 3BNC117/10-1074 infusions

Participants will be discontinued from second or third 3BNC117/10-1074 infusions for any of the following reasons:

1. A disease or condition or an adverse event that may develop, regardless of relationship to 3BNC117 or 10-1074, if the principal investigator or designee is of the opinion that other 3BNC117 or 10-1074 infusions will jeopardize the safety of the participant.
2. Any immediate hypersensitivity reaction (such as urticarial rash; bronchospasm; laryngeal edema; anaphylaxis; syncope).
3. Any grade 2 ophthalmologic adverse event deemed related to the study products, or any grade 3 or 4 ophthalmologic adverse event regardless of causality assessment.
4. Any ALT or AST increase equal to or greater than 3x the upper limit of normal with concurrent increase in total bilirubin equal to or greater than 2x the upper limit of normal. Additionally, such an occurrence will also result in a consultation with a hepatologist to assess for other evidence of drug toxicity.
5. Any participant with a graded elevation in total bilirubin will undergo a diagnostic workup at no cost to the participant that includes right upper quadrant ultrasound, testing for gamma glutamyl transferase (GGT), HAV, HBV, HCV, EBV serologies, CMV serologies, VZV serologies, HSV serologies, and any other relevant laboratory tests as determined by the investigators. Further administration of the investigational products will be discontinued for any participant with a grade 3 or above elevation in total bilirubin for whom this evaluation fails to determine the etiology.
6. Life threatening medical event following 3BNC117 and/or 10-1074 unless not related to the investigational product.
7. Intercurrent use of immunosuppressive medication considered significant by the trial physician (e.g., systemic corticosteroids).
8. Pregnancy.
9. Participant’s request to discontinue further 3BNC117 or 10-1074 infusions.

10. A participant in Groups 2 or 3 who misses the second dose of mAbs or placebo will not receive the third dose.
11. Any participant who misses an infusion will not attend the Day +2 visit following the missed dose.

6.1.7.3 Withdrawal from the study (Early Termination)

Participants may be withdrawn from the study permanently for the following reasons:

1. Participants may withdraw from the study at any time if they wish to do so, for any reason.
2. Following an adverse event at the discretion of the investigator (or designee).
3. Intercurrent HIV infection.
4. Participant judged by the investigator to be at significant risk of failing to comply with the protocol in a manner that might lead to harm to self or seriously interfere with the validity of the study results.
5. At the discretion of the FDA or investigator.

6.1.7.4 Follow up after withdrawal from study (Early Termination)

Any adverse event resulting in withdrawal of a participant will be followed up until resolution or until the adverse event is judged by the principal investigator or designee to have stabilized where possible.

At the time of withdrawal, provided the participant is willing, all the requested termination visit procedures will be performed according to the Time of Events Schedule (**Appendix A**).

The date and reason for withdrawal from the study (early termination) should be collected and reported to the SMC, the Clinical Research Support Office (CRSO) at the Rockefeller University Hospital and the RUH-IRB. Participants who are withdrawn from the study (early termination) will not be replaced, but, wherever possible, will be followed until the time of their final planned visit.

A pregnant participant will not receive 3BNC117 or 10-1074 infusion. If pregnancy occurs after 3BNC117 or 10-1074 infusion, no further infusions will be administered, and the participant will continue to be followed until the end of the study and until delivery, if delivery occurs after the study has ended. Approximately 2-4 weeks after delivery, a pediatrician of the participant's choosing will assess the infant's health status. Rockefeller University will subsidize the cost of this visit. The health status of the infant will be reported to the RUH-IRB, CRSO and the SMC.

6.2 Study Procedures

6.2.1 Consent Procedure

Prior to the initiation of any study related procedures, the potential participants will be given a copy of the most recent IRB stamped and approved informed consent to read. Additionally, the PI or study staff member who has been designated to consent will discuss the specifics of the study including but not limited to the purpose of the research, procedures, time commitment, required tasks, test article, alternative treatments, benefits, risks, confidentiality etc. in a comprehensible (non-scientific) manner, using language readily understandable by the participant. Participants will be told that participation is voluntary and that, if they do not consent, they will not be penalized. The person consenting will assure the voluntariness of the participant.

A private, confidential setting will be provided for the potential participant to read and discuss the informed consent free from coercion, undue influence or constraints of time. All participants will be given a chance to ask questions and express concerns. They will be given the option to take the consent home and discuss it with family, friends, and /or health care providers. After a participant and the person conducting the consenting process sign and date the consent, the participant will be given a copy of the signed informed consent form.

An enrollment note will be written in the source document as to who obtained consent, how, when, were questions asked and answered, and that a copy of the informed consent was given to the participant.

The "Teach Back" method will be used in the clinical research setting to ask research participants to repeat or “teach back” the information, concepts and directions that the staff member has attempted to convey to the participant. This method is used to assess comprehension and retention of protocol requirements, adverse event information, risks and benefits, and the participant's rights described in the Informed Consent process.

6.2.2 Randomization

This study is randomized and double-blinded. Randomized treatment assignments will be generated by the Rockefeller University Hospital pharmacy. The 8 participants in each group will be randomized in a ratio of 6 study drug recipients to 2 placebo recipients. Randomization will be generated using SAS 9.4. Blinding will not apply to the assignment of dose levels (3 or 10 mg/kg). The study preparation will be provided to the study nurses for injection under a coded, masked identification. The placebo and mAb preparations will be indistinguishable in the preparation provided for participants. The nurses, study staff, investigators, and participants will be blinded as to the identity of the study preparation. Participants will be unblinded at the end of the study. To accomplish this, study nurses will be provided with the randomization code after the study has been completed, and they will notify each participant. In the event of a medical emergency wherein knowledge of the treatment assignment will influence the participant’s care, the Principal Investigator may unblind the treatment assignment.

6.2.3 3BNC117 and 10-1074 Administration Procedure

3BNC117 is provided in single-use vials containing 5 ml of the product at a concentration of 20 mg/ml. The volume of 3BNC117 to be administered will be calculated by the site research pharmacist, according to study assignment. The appropriate volume of 3BNC117 will be diluted in sterile normal saline to a total volume of 100 ml, and will be administered as an intravenous infusion over 60 minutes.

10-1074 will be provided in single-use vials containing 5 ml of the product at a concentration of 20 mg/ml. The volume of 10-1074 to be administered will be calculated by the RUH research pharmacist, according to study assignment. The appropriate volume of 10-1074 will be diluted in sterile normal saline to a total volume of 100 ml and will be administered as a slow intravenous infusion over 60 minutes.

- Sterile saline, NaCl 0.9% (placebo) will be dispensed in a volume of 100 ml, and will be administered as a slow intravenous infusion over 60 minutes. A second bag of sterile saline will be administered after the infusion of the first saline bag is completed and the line is flushed.

Both antibodies will be administered intravenously, via a peripheral vein in one of the upper extremities. The administration site should be free of potentially complicating dermatologic conditions. At the end of infusion, the IV line will be flushed with 20 ml of normal saline to ensure all the medication has been delivered.

6.2.4 Medical History and Physical Examination

At the time of screening, participant's past medical history will be collected and will include details of any previous reaction to vaccination, and contraceptive practices. Interim medical histories will be collected at time-points according to the Time of Events Schedule (**Appendix A**).

A general physical examination will be conducted including weight, height, vital signs, and examination of skin, respiratory, cardiovascular, central nervous and abdominal systems. At the time of the first 3BNC117 and 10-1074 infusions and at selected time-points thereafter, general and/or directed physical examinations will be performed according to the Time of Events Schedule (**Appendix A**). A directed physical examination will include vital signs, examination of infusion site, and any further examination indicated by history or observation.

6.2.5 Blood Collection, Storage and Shipment

Venous blood will be collected at every study visit according to the Time of Events Schedule (**Appendix A**). At no time will the total volume of blood collected exceed 550 ml over an 8-week period. All specimens will be handled according to SOPs that were developed in the Processing Lab within the Laboratory of Molecular Immunology.

Frozen PBMCs, plasma and serum will be processed and stored in the Laboratory of Molecular Immunology.

6.2.6 HIV testing and Counseling

Study personnel will assess participants for past and current risk of HIV infection and counsel them prior to collecting blood for an HIV test. Study personnel will perform post HIV-test counseling as indicated in the Time of Events Schedule (**Appendix A**). The counseling process will include information on HIV, safe sex practices and risk reduction. The objective of counseling is to ensure that participants have sufficient knowledge about HIV infection to understand the purpose of the test, the implications of a positive or negative result and the standard of care available locally for HIV infection. Additionally, risk reduction counseling, including safe-sex counseling, will be provided during the study to reinforce low-risk behavior.

6.2.7 HIV Infection

Participants who are found to be HIV-infected at screening and participants who acquire HIV infection during the trial will be withdrawn from the study and managed in the following way:

a) The participant will be counseled by the study investigators. The counseling process will assist the participant with issues such as: psychological and social implications of HIV infection; whom to inform and what to say; implications for sexual partners and avoidance of transmission to others in the future.

b) The participant will then be referred to a patient support center or institution of his/her choice for a full discussion of the clinical aspects of HIV infection. Referral will be made to a designated physician or center for discussion of options of treatment of HIV infection.

6.2.8 Monitoring for cytokine release associated adverse events and treatment of cytokine release syndrome

Based on previous clinical experience, it is unlikely that administration of 3BNC117 or 10-1074 will lead to cytokine release syndrome. However, a potential side effect of a monoclonal antibody can be the stimulation of a massive release of cellular cytokines, which can have profound effects on blood pressure, vascular integrity, and myocardial, lung, liver, and kidney functions. If cytokine release syndrome occurs, the participant may need to be treated with intravenous fluids, vasopressors, and high-dose corticosteroids and may require ventilatory support.

Following the completion of the second infusion, participants enrolled in group 1 will be observed for 4 hours and participants enrolled in groups 2 and 3 will be observed for 1 hour. Access to a twenty-four hour on-call physician is available. The RUH outpatient and inpatient units are equipped with crash carts for immediate medical care, should the need

arise. In case of an emergency, after stabilization of the participant, he/she will be transferred to the neighboring tertiary care center, New York Presbyterian Hospital (Cornell) for specialized medical care.

6.2.9 Family Planning Counseling

During screening and subsequent study visits, study personnel will counsel participants about the importance of prevention of pregnancies and the use of condoms, as well as other effective family planning methods. Condoms will be provided.

Study participants participating in sexual activity that could lead to pregnancy will be advised to use two effective methods of contraception (i.e. condom with spermicide, diaphragm with spermicide, hormone-eluting IUD, hormone-based contraceptive with condom) for the duration of the study.

6.2.10 Reimbursement

Participants will be compensated $25 for the initial screening visit.

They will be compensated:
- $200 for each infusion visit
- $80 for each post infusion follow up visit
- $100 for the final study visit
- $50 for each eye exam

At the Rockefeller site, payment will be made to participants who fill out a form from The Rockefeller University Finance Office and are eligible for and want to receive payment.

<u>In Group 1</u>, participants who complete all study visits will receive a total of $1065 throughout the study.

<u>In Groups 2 and 3</u>, participants who complete all study visits will receive a total of $2,105 throughout the study.

If a member of the study team asks a participant to return for an unscheduled visit, the participant will be compensated $25 each time.

Compensation is provided to help cover their travel expenses, as well as child care and time lost from gainful employment. Participants will be compensated only for the visits they complete.

6.2.11 Safety Assessments

6.2.11.1 Solicited Adverse Events

Solicited adverse events in this study include presence of feverishness, chills, headache, nausea, vomiting, malaise, myalgia and arthralgia occurring in the first 7 days following 3BNC117 and 10-1074 infusions, as well as infusion site reaction or effects caused by extravasation.

Solicited adverse events will be collected prospectively by structured interviews on infusion and post-infusion follow up visits; recorded and graded according to pre-established criteria. The Toxicity Grading Scale for Healthy Adult and Adolescent Volunteers Enrolled in Preventive Vaccine Clinical Trials (2007) will be used to grade adverse events (see **Appendix B)**. In addition, the Common Terminology Criteria for Adverse Events (CTCAE) v4.03 grading scale will be used for reporting and grading adverse events related to infusion reactions and cytokine release syndromes in all groups.

Vital signs (pulse, respiratory rate, blood pressure and temperature) will be measured prior to each antibody administration, at the end, 1 hour and 4 hours (group 1 only) after the second infusion is completed, graded according to **Appendix B** and recorded. All medications required for treatment of adverse events will be recorded.

6.2.11.2 Unsolicited Adverse Events

During all follow up visits, the occurrence of unsolicited adverse events will be assessed following an open question to participants, with the dates of commencement and resolution and any medication required. Adverse events will be followed to resolution or stabilization. They will be graded as indicated in **Appendix B**.

6.2.11.3 Routine Laboratory Parameters

Laboratory parameters will routinely include hematology (WBC and differential, hemoglobin/hematocrit, platelets), clinical chemistry (Creatinine, BUN, Total and Direct bilirubin, AST and ALT, and alkaline phosphatase), and urinalysis (protein, WBCs, RBCs and casts). Female participants will have serum beta-HCG measured at screening and on the day of each 3BNC117 and 10-1074 infusions, and urine beta-HCG checked during follow up visits. The laboratory samples for these tests will be collected at the time points indicated in the Time of Events Schedule (**Appendix A**).

In the event of an abnormal laboratory value, participants may be asked to have additional sample(s) collected at the discretion of the principal investigator or designee.

Participants will be screened for syphilis (RPR) and viral hepatitis (HBsAg and HCV viral load) at the Screening Visit. Anti-nuclear antibody will also be checked at screening.

6.2.11.4 Assays

1. Measurement of 3BNC117 and 10-1074 serum levels by sandwich ELISA will be performed at Celldex Therapeutics.

2. Anti-drug (10-1074) antibody responses in serum. Assays will be performed at Celldex Therapeutics Inc.

3. TZM-bl neutralization assay will be performed in the laboratory of Dr. Michael Seaman of the Beth Israel Deaconess Hospital in Boston (a Core Laboratory sponsored by the Collaboration for AIDS Vaccine Discovery, CAVD). *In vitro* neutralization assays with serum from study participants from all study groups, before and after administration of 3BNC117 and 10-1074 will be performed to measure 3BNC117 and 10-1074 *in vivo* neutralizing activity against a panel of pseudoviruses representative of multiple viral clades.

Optimal sample collection, processing, cryopreservation, archiving and storage will be maintained. Additional studies will be performed as warranted at the discretion of the investigators.

6.2.11.5 Pharmacokinetic evaluations

3BNC117 and 10-1074 serum levels will be measured by validated sandwich ELISA methods performed at Celldex Therapeutics. Serum samples will be collected before and at the end of each 3BNC117 and of each 10-1074 infusion, at 2 days and 1, 2, 3 and 4 weeks after respective infusions, and at later time points as outlined in **Appendix A**.

Briefly, serum samples, positive and negative controls, and multiple dilutions of a 3BNC117 or 10-1074 reference standard will be incubated in plates coated with a murine anti-idiotype antibody to 3BNC117 or a murine anti-idiotypic antibody to 10-1074 for the respective assays. Immobilized 3BNC117 will be detected with a murine anti-human IgG kappa chain mAb-HRP conjugate, and immobilized 10-1074 will be detected with an affinity-purified $F(ab')_2$ fragment goat polyclonal anti-human IgG Fc gamma chain specific-HRP conjugate. Colorimetric detection will be afforded with HRP substrate, tetra-methylbenzidine.

The concentration of 3BNC117 or 10-1074 in the samples will be interpolated from a standard curve of 3BNC117 or 10-1074, using a logistic curve-fitting algorithm. The reference standard and positive controls will be created from the drug product lot of 3BNC117 or 10-1074.

Pharmacokinetic parameters, including AUC, Cmax, $T_{1/2}$, Tmax and others will be estimated by performing a non-compartmental analysis (NCA) using WinNonlin 6.3. ANOVA model will be used to compare cohort differences for AUC and Cmax. The dose proportionality for the 2 parameters (AUC and Cmax) will be examined by regression analysis. Exposure parameters will be compared between single-and multiple-dose regimens to evaluate the extent of drug accumulation and the impact on clearance. Pharmacokinetic parameters will be examined to correlate exposure with safety and pharmacodynamic parameters (decline in plasma HIV-1 RNA), and variance, based on

population intrinsic factors such as weight and gender, will be explored. In addition, we will compare pharmacokinetic parameters when 3BNC117 and 10-1074 are given in combination to historical monotherapy data with either antibody.

7 Investigational Products

Investigational Drug Name:	3BNC117
Manufacturer name of drug:	Celldex Therapeutics, Inc.
IND Number:	118,225
IND Sponsor:	Sarah Schlesinger, MD

Investigational Drug Name:	10-1074
Manufacturer name of drug:	Celldex Therapeutics, Inc.
IND Number:	123,713
IND Sponsor:	Sarah Schlesinger, MD

7.1 Regimen

3BNC117 and 10-1074 will be administered intravenously at 3 or 10 mg/kg.

- Group 1: one infusion of 3BNC117 and 10-1074 at day 0, each dosed at 10 mg/kg.
- Group 2: three infusions of 3BNC117 and 10-1074, each dosed at 3 mg/kg, at day 0, weeks 8 and 16.
- Group 3: three infusions of 3BNC117 and 10-1074, each dosed at 10 mg/kg, at day 0, weeks 8 and 16.

7.2 Study Product Formulation and Preparation

3BNC117 is provided by Celldex Therapeutics in single-use vials containing 5 ml of 3BNC117 at a 20 mg/ml concentration. 3BNC117 will be diluted in normal saline (NaCl 0.9%) to a volume of 100 ml.

10-1074 is provided by Celldex Therapeutics in single-use vials containing 5 ml of 10-1074 at a 20 mg/ml concentration. 10-1074 will be diluted in normal saline (NaCl 0.9%) to a volume of 100 ml.

Sodium chloride 0.9% provided from a commercial source will be used as the placebo at a volume of 100ml.

7.3 Dispensing and Handling of Investigational Product

3BNC117 and 10-1074 will be shipped from Celldex Therapeutics and will be stored in the RUH Pharmacy at 2 - 8°C. Both products will be dispensed by the RUH pharmacist. Trial personnel will ensure that the study ID number on the infusion bag matches the study ID assigned to the participant prior to administration.

The appropriate dose will be calculated by the RU pharmacist according to study allocation and participant's weight. Both products will be dispensed in a piggy-back, diluted in normal saline (NaCl 0.9%), ready for administration by the study investigators.

7.4 Accountability and Disposal of Used and Unused Investigational Product

The date, allocation number and location of storage of the vials will be recorded in a log. During the trial, the product accountability form, and the dispensing log will be monitored. At the end of the trial, unused vials will be returned to Celldex Therapeutics or destroyed.

8 Data Analysis

8.1 Analysis of Safety, PK and Antiretroviral effects

Primary Outcomes

- Safety: The safety population will include all participants who receive placebo or 3BNC117 and 10-1074 infusions. A baseline measurement and at least one laboratory, vital sign, or other safety-related measurement obtained after placebo or 3BNC117 and 10-1074 infusions may be required for inclusion in the analysis of a specific safety parameter.

The number and percentage of participants experiencing one or more AEs will be summarized by study group, relationship to study drug, and severity. AEs will be summarized by the number and percentage of participants who experienced the event, according to system organ class (SOC) and preferred term. AEs will also be summarized by severity grade and by relationship to study drug according to the Toxicity Grading Scale for Healthy Adult and Adolescent Volunteers Enrolled in Preventive Vaccine Clinical Trials. The CTCAE v4.03 grading scale will be used for reporting and grading adverse events related to infusion reactions and cytokine release syndromes in all groups (**Appendix B**). Changes in hematology, chemistry, and other laboratory values will be summarized descriptively. Changes will be calculated relative to the values collected at baseline.

- Pharmacokinetic parameters will be calculated using standard non-compartmental analysis methods. Descriptive results will be presented for the pharmacokinetic parameters by the 3 study groups. Pharmacokinetic parameters, including AUC, Cmax, $t_{1/2}$, Tmax and others will be summarized using descriptive statistics. ANOVA model will be used to compare group differences for AUC and Cmax. Regression analyses will be performed with AUC and Cmax as dependent variables and dose group as an independent variable. Population intrinsic factors, such as weight and gender, will be included in the regression models to assess whether these factors modify the relationship between dose and pharmacokinetic parameters.

Secondary outcomes

- Anti-10-1074 antibodies: The frequency of induced anti-3BNC117 and anti-10-1074 antibodies at 8 weeks following each infusion will be reported for all 3 groups and the proportion will be compared using Fisher's Exact test. The levels of the anti-antibodies will be compared using ANOVA or Kruskal-Wallis test.

Exploratory measurements

- In vitro neutralization assays will be performed with serum from study participants to evaluate antiretroviral activity against Tier 1 and Tier 2 HIV strains. Results will be descriptive.

The analysis of study data will be primarily descriptive, with emphasis on tabular and graphical displays. This study is exploratory, and any statistical inferences will be hypothesis generating and not confirmatory.

8.2 Sample Size Considerations

Safety:
The safety population will include all participants who receive at least one dose of study treatment. A baseline measurement and at least one laboratory, vital sign, or other safety-related measurement obtained after at least one dose of study treatment will be required for inclusion in the analysis of a specific safety parameter.

With 6 placebo participants, the 95% upper binomial confidence limit for the proportion with adverse events is 46%. For the proposed 18 participants when combining all three study treatment groups, the upper limit for the proportion with adverse events is 19%. The table below shows the upper 95% confidence limits for the binomial proportion when no event has been observed for sample sizes ranging from 6 to 20.

Table 1. Safety assessments: Upper 95% confidence limits for the binomial proportion

Sample Size	Proportion	Sample Size	Proportion	Sample Size	Proportion
6	0.46	11	0.28	16	0.21
7	0.41	12	0.26	17	0.20
8	0.37	13	0.25	18	0.19
9	0.34	14	0.23	19	0.18
10	0.31	15	0.22	20	0.17

The Fisher's exact test will be used to compare the proportion of adverse events in the placebo and treatment groups.

Pharmacokinetics:
The 95% margin of error for estimating the mean elimination half-life of 3BNC117 plus 10-1074 is summarized below for Group 2 (n=6) and Group 3 (n=6) based on standard deviations from 4.5 days to 8.0 days using the pharmacokinetics data of 3BNC117 administered alone at 3 mg/kg and 10 mg/kg in healthy participants.

Table 2. Pharmacokinetics: 95% margin or error for estimating mean elimination half-life of the study products

Number of participants	Standard deviation (days)	95% margin of error (+/- days)
6	4.5	3.6
	5.0	4.0
	5.5	4.4
	6.0	4.8
	6.5	5.2
	7.0	5.6
	7.5	6.0
	8.0	6.4
12 (Groups 2 and 3)	4.5	2.5
	5.0	2.8
	5.5	3.1
	6.0	3.4
	6.5	3.7
	7.0	4.0
	7.5	4.2
	8.0	4.5

8.3 Analyses prior to the end of the study

Blinded analyses of pharmacokinetics and serum neutralization may be performed after all subjects in a particular group complete the final study visit. An unblinded analysis may be performed by an unblinded statistician. These analyses will allow a more rapid assessment to facilitate program development. Blinding of study staff will be maintained.

9 Data and Sample Storage

The Principal Investigator will oversee how the data are collected, entered, and protected. All study data will be collected by the clinical study staff using designated source documents and entered onto the appropriate electronic case report forms (eCRFs). Data collection forms (DCFs) will be provided by Emmes for use as source documents as appropriate. All study data must be verifiable to the source documentation. All source documents will be kept in a locked facility at the clinical site and remain separate from participant identification information (name, address, etc.) to ensure confidentiality. All medical records (when not being reviewed by the research team) will be kept under lock and key in the Medical Record Department of the hospital with access limited to the

appropriate RUH personnel, members of the RUH-IRB and the FDA. Source documentation will be available for review to ensure that the collected data are consistent with the eCRFs.

All eCRFs and laboratory reports will be reviewed by the clinical team, who will ensure that they are accurate and complete.

All research samples will have a unique identifier. The PI will be responsible for ensuring project compliance, data analysis and entry, regulatory monitoring, and coordination of the activities of the entire study team. Standard GCP practices will be followed to ensure accurate, reliable and consistent data collection.

Source documents include, but are not limited to:

- Signed Informed Consent Documents
- Dates of visits including dates of infusion
- Documentation of any existing conditions or past conditions relevant to eligibility
- Reported laboratory results
- All adverse events
- Concomitant medications
- Reactogenicity adverse events

The PI and sponsor (Rockefeller University) are the owners of the data. The PI will take primary responsibility for the data. Data will first be collected on paper charts and then transferred into a secure online database managed by the Emmes Corporation. Access to the Emmes website is granted only after an authorized individual (e.g., PI, Study Coordinator or supervising monitor at Rockefeller University) grants permission and a staff member completes a roster form stating his or her study role/responsibilities and contact information. Access to the data system requires a different username and password from the Emmes website, and access can be tailored based on the user's role/ responsibilities in the study. The only protected health information entered into the Emmes database is participants' DOB. This is done in order to confirm that a participant meets age-eligibility criteria. DOB is then converted to age and does not appear on any data summaries sent from Emmes. All Emmes websites use SSL for secure transport. The clinical study team will have access to the Emmes database, however, only a single study team member will input data into the database. Changes to the database are tracked by user and time of entry. Password-protected data summaries that contain no protected health information are released to the PI from Emmes through ELF and will be secured on the PI's encrypted password protected computer. Study team members may also store these data summaries on their own encrypted password-protected computers.

De-identified blood samples will be sent to a collaborator at Beth Israel Deaconess and the resulting data will be returned by email to the PI. The Exchange email system will be used by our collaborator to report the result of the assay. These reports contain no protected health information.

10 Recruitment and Advertising

Both men and women ages 18 through 65 will be recruited for the study from the community at large and will be referred by physicians in the community. We will make every effort to recruit minorities and women.

We project screening 72 participants in order to achieve at least 24 evaluable participants. In case of drop-outs an over-enrollment of 10% (2 participants) will be allowed.

Advertisements – The RUH Clinical Research Support Office (CRSO) will utilize the Volunteer Repository. Advertisements will also be placed: online (e.g. Craiglist, Centerwatch, etc), in newspapers (Metro, AMNY) and on campus.

The Research Match tool will also be utilized.

Centralized Call Management – The RUH CRSO will conduct telephone screenings of selected Volunteer Repository members, and of volunteers who call 1800-RUCARES, to facilitate screening efficiently. Based on IRB approved eligibility criteria, potentially eligible candidates pre-screened by CRSO staff will be referred to the study coordinator/investigator for further evaluation. The research team and CRSO will work together on a protocol-specific pre-screening script to optimize the process.

11 Potential Benefits to Participants

There are no direct benefits to participants that enroll in this study.

12 Potential risks to the participant

This study entails moderate risk to participants since 3BNC117 and 10-1074 are investigational products with limited safety data in humans. This is the first time that both antibodies will be administered in combination and the first time that 3 doses of 10-1074 will be administered. Potential study participants will be informed about the possible risks associated with 3BNC117 and 10-1074 infusions and that there may be unknown risks.

<u>Potential risks associated with the combination of 3BNC117 and 10-1074</u>:

While each mAb product has unique safety issues related to its mechanism of action, the major safety concern related to mAbs in general is an infusion/hypersensitivity reaction. These types of reactions are more common for mAbs that contain murine elements compared to human mAbs, such as 3BCN117 and 10-1074. Passive administration of anti-HIV-1 antibodies has been evaluated in humans in the past. As observed with other monoclonal antibodies, anti-HIV-1 antibodies, including 3BNC117 and 10-1074 have been found to be generally safe and well tolerated and most adverse events observed were transient myalgia, fatigue and headache.

3BNC117 and 10-1074 have not been administered together in the past. However earlier generation anti-HIV antibodies (2F5, 4E10, 2G12) were administered intravenously in combination at doses ranging from 1 to 2 g per infusion of each antibody (or 12.5 to 25 mg.kg, assuming 80 kg body weight); 13-16 weekly antibody infusions were given (Trkola, Kuster et al. 2005) (Mehandru, Vcelar et al. 2007). In these two studies, antibody infusions were well tolerated and reported side effects were mild and transient. Most commonly reported adverse events were fatigue, body aches, arthralgia, and low-back ache. No serious adverse events (SAEs) were recorded. In addition, in a GLP-study, rats were administered 4 weekly doses of 3BNC17 and 10-1074 at 60 mg/kg. Based on the results of this study, and absence of apparent direct toxicity of the test item in the presence of apparent immunogenicity, the No-Observed-Adverse-Effect-Level (NOAEL) of the combination intravenous dose of 10-1074 and 3BNC117 in rats was considered to be 60 mg/kg/injection, each administered once weekly, for up to 25 days. Although unlikely, it is possible that the combination of 3BNC117 and 10-1074 will lead to a higher frequency of adverse events and/or to adverse events of higher severity than either antibody administered alone

- Immunologic symptoms such as listed below are possible with administration of a monoclonal antibody and will be considered adverse events of interest. Potential allergic-type reactions during and immediately following the administration of the antibodies will be carefully monitored.
  - Constitutional symptoms, such as fever, rigors/chills;
  - Infusion site reaction/extravasation changes, pruritus, urticaria;
  - Serum sickness like syndromes as evidenced by fever, rash, arthralgia, arthritis, nephritis;
  - Deposition of immune complexes in the kidneys leading to renal insufficiency;
  - Adult Respiratory Distress Syndrome, bronchospasm/wheezing, anaphylaxis;
  - Cytokine release syndrome/ acute infusion reaction.

- In the cross-reactivity study in human tissues, 3BNC117 stained rare cells in the conjunctival recesses. When rats were administered 3BNC117, ocular toxicity was not observed. Sixty-eight participants have received 3BNC117 to date, and 12 participants reported mild ophthalmic complaints (such as pruritus, conjunctival erythema, increased lacrimation) during study follow up. In all instances symptoms resolved without specific treatment and ophthalmologic evaluations 5 months after 3BNC117 administration did not show changes from baseline.

- In the cross-reactivity study in human tissues, 10-1074 stained the cytoplasm of nerve cells in the optic nerve of the eye. Twenty-two participants have received 10-1074 to date. One participant reported transient and mild dryness in the eyes.

- Participants might have false positive HIV serology results after 3BNC117 and 10-1074 administration. This will likely be temporary, while antibody levels are detectable in blood.

- The adverse effects of 3BNC117 and 10-1074 to a fetus or unborn child are unknown.

Other potential risks

- Blood drawing and phlebotomy can be associated with pain, bruising, anemia or infection at the site of venipuncture. Rarely, fainting may follow phlebotomy.

- Participants may engage in increased risk taking after receiving anti-HIV mAb infusion. One to one counseling will be routinely performed.

12.1 Procedures to minimize risk

- As outlined above, this study will be an exploratory phase 1 trial of the combination of 3BNC117 and 10-1074. Potential study participants will be informed about possible risks of the monoclonal antibody and that there may be unknown risks.

- Medical records and routine laboratory data will be handled with HIPAA compliance and protected by the rules and regulations of the RUH, a JCAHO approved institution.

- Following enrollment in Group 1, enrollment in subsequent group will only begin following a review of Day 28 safety data from all participants in the prior group.

- To minimize risks associated with phlebotomy, blood drawing will be performed by experienced phlebotomists.

- To minimize risks associated with blood drawing, participants will be closely monitored for signs and symptoms of anemia.

- Females of childbearing potential and who participate in sexual activity that might lead to pregnancy will be advised to use two reliable forms of contraception (i.e. condom with spermicide, diaphragm with spermicide, hormone-eluting IUD, hormone-based contraceptive with condom) for the duration of the study. In addition, a pregnancy test will be performed at screening, on the day of each drug infusion, and other times throughout the course of the study. Males who are not anatomically sterile and who participate in sexual activity that might lead to pregnancy will be advised to use two reliable forms of contraception from 10 days prior to the 3BNC117/10-1074 infusion until the end of the study to avoid pregnancy in a spouse or partner. Condoms will be provided.

- Participants will have regularly scheduled visits to the outpatient clinic and routine safety laboratories will be checked according to the Time of Events Schedule (**Appendix A**).

- Adverse events will be monitored and graded using the Toxicity Grading Scale for Healthy Adult and Adolescent Volunteers Enrolled in Preventive Vaccine Clinical Trials (HIV-uninfected). The CTCAE v4.03 grading scale will be used for reporting

and grading adverse events related to infusion reactions and cytokine release syndromes in all groups (**Appendix B**).

- Adverse events will be managed by the clinical trial team who will assess and treat the event as appropriate, including referral to an independent physician and/or department.

- Safety monitoring will be conducted by the study investigators, by an external Study Monitoring Committee (SMC), and by the International AIDS Vaccine Initiative (IAVI). The RUH-IRB will conduct the initial review of the proposed study and will follow progress through annual reports and by immediate notification of serious adverse events. Any serious and unanticipated adverse events will be immediately reviewed by the study investigators. Investigators will notify the RUH-IRB within 2 working days from the investigators being made aware of the event. The RU sponsor will notify the FDA, per 21 CFR 312. The SMC will be available to the investigators for consultation and review of severe adverse events if needed.

13 Data and Safety Monitoring Plan

This is a phase 1 study, which exposes study participants to "moderate risk". A Study Monitoring Committee (SMC) will be established to monitor the study.

13.1 Safety Monitoring Committee and Stopping Rules

The charter of the Safety Monitoring Committee (SMC) is to provide an ongoing assessment of participant safety during the conduct of the study. The SMC consists of three independent individuals who have no relationship to the Principal Investigator and Co-Investigators involved in the trial. No member of the SMC will have any direct responsibility for the clinical care of study participants. No representative of Celldex Therapeutics, the Rockefeller University, or their designees may be a member of the SMC. However, the SMC may invite the principal investigator (PI) or designee and a Celldex Therapeutics, and/or Rockefeller University representative to an open session of a SMC meeting to provide information on study conduct, present data, or to respond to the members' questions. Dr. Pat Fast, from the International AIDS Vaccine Initiative (IAVI) can be invited to participate in SMC meetings and to comment on study occurrences, as the medical monitor for the study.

The names, university affiliation and title, area of expertise, and contact information of each of the SMC members are provided below:

Raphael Dolin, MD
Maxwell Finland Professor of Medicine
Beth Israel Deaconess Medical Center
Harvard Medical School
330 Brookline Ave
Boston MA, 02215
Email: rdolin@bidmc.harvard.edu

Areas of expertise: HIV vaccine clinical trials

Magdalena Sobieszczyk, MD, MPH
Associate Professor of Medicine
Columbia University College of Physicians and Surgeons
622 W 168th St
New York, NY 10032
Email: mes52@cumc.columbia.edu
Areas of expertise: HIV vaccine clinical trials

Michael Keefer, MD
Professor of Medicine
University of Rochester Medical Center
601 Elmwood Ave, Box 689
Rochester, NY 14642
Email: Michael_keefer@urmc.rochester.edu
Areas of expertise: HIV vaccine clinical trials

At least two members of the SMC must be in attendance (phone, video, or in-person meetings) to constitute a quorum for an SMC meeting. SMC members may also review and comment by email, if scheduling cannot be worked out in a timely manner. One member of the SMC will be appointed as chair of the committee. The SMC chair (or his/her alternate) will be responsible for summarizing and communicating in writing SMC acknowledgments and recommendations to the PI within 5 business days following each SMC meeting and/or review.

The SMC will be asked to review study data in the following situations:

1. If > 1 grade 3 or higher adverse events deemed probably or definitely related to the study drugs occur in a single group, no additional administration of the investigational product will take place pending a Safety Monitoring Committee (SMC) review. The SMC will provide a recommendation regarding subsequent enrollment in the study.

2. If there is one SAE judged as possibly, probably or definitely related to the administration of 3BNC117 and/or 10-1074 by the principal investigator or designee, no additional administration of the investigational product will take place pending a review by at least two members of the SMC. Following this review, the SMC will make a recommendation to the principal investigator regarding the continuation of investigational product administration.

SAEs, which are deemed possibly, probably or definitely related to 3BNC117 and/or 10-1074 by the principal investigator or designee, and unanticipated adverse events will be reported to the SMC within 2 working days of the site becoming aware of the event.

3. If, at any time, a fatal, life-threatening or permanently disabling SAE with a suspected causal relationship to 3BNC117 and/or 10-1074 occurs, no further administration of the

investigational products will occur until a consensus plan forward has been approved by investigators, SMC, the IRB, and the FDA.

4. The SMC will be informed within 2 working days of any participant who discontinues study treatments as a result of a grade 2 ophthalmologic adverse event deemed related to the study products or a grade 3 or 4 ophthalmologic adverse event deemed unrelated to the study drugs.

5. The SMC will be informed within 2 working days of any participant who discontinues receipt of the study products as a result of liver test abnormalities as described in Section 6.1.7.2.

6. In addition, the SMC will be asked to review on an interim basis:
- Severe solicited and unsolicited adverse events judged by the principal investigator or designee to be possibly, probably or definitely related to 3BNC117 and/or 10-1074.
- Severe laboratory adverse events confirmed on retest and judged by the principal investigator or designee to be possibly, probably or definitely related to 3BNC117 and/or 10-1074.

The occurrence of such adverse events will not result in a study pause, unless it is judged by the principal investigator or designee that the risk/benefit ratio of the study has changed such that risk of currently enrolled or future participants has increased; or unless recommended by the IRB, SMC, or FDA.

All updated versions of the protocol will be provided to the SMC. Review of unblinded trial data by the SMC will take place at least annually. Prior to data review, the study team will provide the SMC with updated records of all adverse events (AEs) of a grade 2 or higher.

The SMC will acknowledge receipt of annual reports and will indicate if there are concerns with the continuation of the study.

13.2 Monitoring

Safety monitoring will be conducted by the study investigators, by the external Study Monitoring Committee (SMC) and by the International AIDS Vaccine Initiative (IAVI). IAVI. The RUH-IRB will conduct the initial review of the proposed study and will follow progress through annual reports and by immediate notification of serious adverse events. External monitoring will occur at least quarterly, and will be conducted by IAVI.

13.3 Adverse Event Classification

Adverse events will be graded using the Toxicity Grading Scale for Healthy Adult and Adolescent Volunteers Enrolled in Preventive Vaccine Clinical Trials (HIV-uninfected).

The CTCAE v4.03 grading scale will be used for reporting and grading adverse events related to infusion reactions and cytokine release syndromes in all groups (**Appendix B**).

13.4 Reporting Adverse Events

All adverse events will be reported to the IRB and the SMC at least annually. Serious Adverse Events, (SAEs) will be reported to the IRB and the SMC, within two working days of identification of the SAE. SAEs will be reported directly to the FDA, per 21 CFR 312.

13.5 Reporting Unanticipated AEs

Unanticipated Adverse Events (UAEs) will be reported to the IRB and SMC. UAEs that are related and greater than moderate severity must be reported to the IRB and SMC, within two working days of identification of the UAE. UAEs will be reported to the FDA, per 21 CRF 312.

13.6 Clinical Laboratory Improvement Amendment/Clinical Laboratory Evaluation Program (CLIA/CLEP)

This study includes tests that are not CLIA/CLEP certified. The results of such tests will not be used in clinical decision-making or shared with participants or their health care providers.

14 Clinical Trial Registration

The proposed study involves testing of FDA regulated drugs or biologics and will be registered at www.ClinicalTrials.gov.

15 Bibliography

Armbruster, C., G. M. Stiegler, B. A. Vcelar, W. Jager, U. Koller, R. Jilch, C. G. Ammann, M. Pruenster, H. Stoiber and H. W. Katinger (2004). "Passive immunization with the anti-HIV-1 human monoclonal antibody (hMAb) 4E10 and the hMAb combination 4E10/2F5/2G12." J Antimicrob Chemother **54**(5): 915-920.

Baeten, J. M., D. Donnell, P. Ndase, N. R. Mugo, J. D. Campbell, J. Wangisi, J. W. Tappero, E. A. Bukusi, C. R. Cohen, E. Katabira, A. Ronald, E. Tumwesigye, E. Were, K. H. Fife, J. Kiarie, C. Farquhar, G. John-Stewart, A. Kakia, J. Odoyo, A. Mucunguzi, E. Nakku-Joloba, R. Twesigye, K. Ngure, C. Apaka, H. Tamooh, F. Gabona, A. Mujugira, D. Panteleeff, K. K. Thomas, L. Kidoguchi, M. Krows, J. Revall, S. Morrison, H. Haugen, M. Emmanuel-Ogier, L. Ondrejcek, R. W. Coombs, L. Frenkel, C. Hendrix, N. N. Bumpus, D. Bangsberg, J. E. Haberer, W. S. Stevens, J. R. Lingappa and C. Celum (2012). "Antiretroviral prophylaxis for HIV prevention in heterosexual men and women." N. Engl. J. Med. **367**(5): 399-410.

Caskey, M., F. Klein, J. C. C. Lorenzi, M. S. Seaman, A. P. West Jr., N. Buckley, G. Kremer, L. Nogueira, M. Braunschweig, J. F. Scheid, J. A. Horwitz, I. Shimeliovich, S. Ben-Avraham, M. Witmer-Pack, M. Platten, C. Lehmann, L. A. Burke, T. Hawthorne, R. J. Gorelick, B. D. Walker, T. Keler, R. M. Gulick, G. Fatkenheuer, S. J. Schlesinger and M. C. Nussenzweig (2015). "Viraemia suppressed in HIV-1-infected humans by broadly neutralizing anitbody 3BNC117." Nature **522**:487-489.

Cohen, M. S. and L. R. Baden (2012). "Preexposure prophylaxis for HIV--where do we go from here?" N. Engl. J. Med. **367**(5): 459-461.

Grant, R. M., J. R. Lama, P. L. Anderson, V. McMahan, A. Y. Liu, L. Vargas, P. Goicochea, M. Casapia, J. V. Guanira-Carranza, M. E. Ramirez-Cardich, O. Montoya-Herrera, T. Fernandez, V. G. Veloso, S. P. Buchbinder, S. Chariyalertsak, M. Schechter, L. G. Bekker, K. H. Mayer, E. G. Kallas, K. R. Amico, K. Mulligan, L. R. Bushman, R. J. Hance, C. Ganoza, P. Defechereux, B. Postle, F. Wang, J. J. McConnell, J. H. Zheng, J. Lee, J. F. Rooney, H. S. Jaffe, A. I. Martinez, D. N. Burns and D. V. Glidden (2010). "Preexposure chemoprophylaxis for HIV prevention in men who have sex with men." N. Engl. J. Med. **363**(27): 2587-2599.

Haynes, B. F., P. B. Gilbert, M. J. McElrath, S. Zolla-Pazner, G. D. Tomaras, S. M. Alam, D. T. Evans, D. C. Montefiori, C. Karnasuta, R. Sutthent, H. X. Liao, A. L. DeVico, G. K. Lewis, C. Williams, A. Pinter, Y. Fong, H. Janes, A. DeCamp, Y. Huang, M. Rao, E. Billings, N. Karasavvas, M. L. Robb, V. Ngauy, M. S. de Souza, R. Paris, G. Ferrari, R. T. Bailer, K. A. Soderberg, C. Andrews, P. W. Berman, N. Frahm, S. C. De Rosa, M. D. Alpert, N. L. Yates, X. Shen, R. A. Koup, P. Pitisuttithum, J. Kaewkungwal, S. Nitayaphan, S. Rerks-Ngarm, N. L. Michael and J. H. Kim (2012). "Immune-correlates analysis of an HIV-1 vaccine efficacy trial." N. Engl. J. Med. **366**(14): 1275-1286.

Hessell, A. J., P. Poignard, M. Hunter, L. Hangartner, D. M. Tehrani, W. K. Bleeker, P. W. Parren, P. A. Marx and D. R. Burton (2009). "Effective, low-titer antibody protection against low-dose repeated mucosal SHIV challenge in macaques." Nat. Med. **15**(8): 951-954.

Horwitz, J. A., A. Halper-Stromberg, H. Mouquet, A. D. Gitlin, A. Tretiakova, T. R. Eisenreich, M. Malbec, S. Gravemann, E. Billerbeck, M. Dorner, H. Buning, O.

Schwartz, E. Knops, R. Kaiser, M. S. Seaman, J. M. Wilson, C. M. Rice, A. Ploss, P. J. Bjorkman, F. Klein and M. C. Nussenzweig (2013). "HIV-1 suppression and durable control by combining single broadly neutralizing antibodies and antiretroviral drugs in humanized mice." Proc. Natl. Acad. Sci. USA **110**(41): 16538-16543.

Klein, F., A. Halper-Stromberg, J. A. Horwitz, H. Gruell, J. F. Scheid, S. Bournazos, H. Mouquet, L. A. Spatz, R. Diskin, A. Abadir, T. Zang, M. Dorner, E. Billerbeck, R. N. Labitt, C. Gaebler, P. M. Marcovecchio, R. B. Incesu, T. R. Eisenreich, P. D. Bieniasz, M. S. Seaman, P. J. Bjorkman, J. V. Ravetch, A. Ploss and M. C. Nussenzweig (2012). "HIV therapy by a combination of broadly neutralizing antibodies in humanized mice." Nature **492**(7427): 118-122.

Klein, F., H. Mouquet, P. Dosenovic, J. F. Scheid, L. Scharf and M. C. Nussenzweig (2013). "Antibodies in HIV-1 vaccine development and therapy." Science **341**(6151): 1199-1204.

Kong R, Louder MK, et al. (2015). “Improving neutralization potency and breadth by combining broadly reactive HIV-1 antibodies targeting major neutralization epitopes”. J Virol. **89**(5):2659-71.

Ledgerwood, J. E., E. E. Coates, G. Yamshchikov, J. G. Saunders, L. Holman, M. E. Enama, A. DeZure, R. M. Lynch, I. Gordon, S. Plummer, C. S. Hendel, A. Pegu, M. Conan-Cibotti, S. Sitar, R. T. Bailer, S. Narpala, A. McDermott, M. Louder, S. O'Dell, S. Mohan, J. P. Pandey, R. M. Schwartz, Z. Hu, R. A. Koup, E. Capparelli, J. R. Mascola, B. S. Graham and V. R. C. S. Team (2015). "Safety, pharmacokinetics and neutralization of the broadly neutralizing HIV-1 human monoclonal antibody VRC01 in healthy adults." Clin Exp Immunol **182**(3): 289-301.

Lynch, R. M., E. Boritz, E. E. Coates, A. DeZure, P. Madden, P. Costner, M. E. Enama, S. Plummer, L. Holman, C. S. Hendel, I. Gordon, J. Casazza, M. Conan-Cibotti, S. A. Migueles, R. Tressler, R. T. Bailer, A. McDermott, S. Narpala, S. O'Dell, G. Wolf, J. D. Lifson, B. A. Freemire, R. J. Gorelick, J. P. Pandey, S. Mohan, N. Chomont, R. Fromentin, T. W. Chun, A. S. Fauci, R. M. Schwartz, R. A. Koup, D. C. Douek, Z. Hu, E. Capparelli, B. S. Graham, J. R. Mascola, J. E. Ledgerwood and V. R. C. S. Team (2015). "Virologic effects of broadly neutralizing antibody VRC01 administration during chronic HIV-1 infection." Sci Transl Med **7**(319): 319ra206.

Marrazzo, J. M., G. Ramjee, B. A. Richardson, K. Gomez, N. Mgodi, G. Nair, T. Palanee, C. Nakabiito, A. van der Straten, L. Noguchi, C. W. Hendrix, J. Y. Dai, S. Ganesh, B. Mkhize, M. Taljaard, U. M. Parikh, J. Piper, B. Masse, C. Grossman, J. Rooney, J. L. Schwartz, H. Watts, M. A. Marzinke, S. L. Hillier, I. M. McGowan and Z. M. Chirenje (2015). "Tenofovir-based preexposure prophylaxis for HIV infection among African women." The New England journal of medicine **372**(6): 509-518.

Mascola, J. R., M. G. Lewis, G. Stiegler, D. Harris, T. C. VanCott, D. Hayes, M. K. Louder, C. R. Brown, C. V. Sapan, S. S. Frankel, Y. Lu, M. L. Robb, H. Katinger and D. L. Birx (1999). "Protection of macaques against pathogenic simian/human immunodeficiency virus 89.6PD by passive transfer of neutralizing antibodies." J. Virol. **73**(5): 4009-4018.

Mehandru, S., B. Vcelar, T. Wrin, G. Stiegler, B. Joos, H. Mohri, D. Boden, J. Galovich, K. Tenner-Racz, P. Racz, M. Carrington, C. Petropoulos, H. Katinger and M. Markowitz (2007). "Adjunctive passive immunotherapy in human immunodeficiency

virus type 1-infected individuals treated with antiviral therapy during acute and early infection." J Virol **81**(20): 11016-11031.

Moldt, B., E. G. Rakasz, N. Schultz, P. Y. Chan-Hui, K. Swiderek, K. L. Weisgrau, S. M. Piaskowski, Z. Bergman, D. I. Watkins, P. Poignard and D. R. Burton (2012). "Highly potent HIV-specific antibody neutralization in vitro translates into effective protection against mucosal SHIV challenge in vivo." Proc. Natl. Acad. Sci. USA **109**(46): 18921-18925.

Onyango-Makumbi, C., S. B. Omer, M. Mubiru, L. H. Moulton, C. Nakabiito, P. Musoke, F. Mmiro, S. Zwerski, H. Wigzell, L. Falksveden, B. Wahren, G. Antelman, M. G. Fowler, L. Guay and J. B. Jackson (2011). "Safety and efficacy of HIV hyperimmune globulin for prevention of mother-to-child HIV transmission in HIV-1-infected pregnant women and their infants in Kampala, Uganda (HIVIGLOB/NVP STUDY)." J. Acquir. Immune Defic. Syndr. **58**(4): 399-407.

Plotkin, S. A. (2010). "Correlates of protection induced by vaccination." Clinical and Vaccine Immunology : CVI **17**(7): 1055-1065.

Scheid, J. F., H. Mouquet, B. Ueberheide, R. Diskin, F. Klein, T. Y. Oliveira, J. Pietzsch, D. Fenyo, A. Abadir, K. Velinzon, A. Hurley, S. Myung, F. Boulad, P. Poignard, D. R. Burton, F. Pereyra, D. D. Ho, B. D. Walker, M. S. Seaman, P. J. Bjorkman, B. T. Chait and M. C. Nussenzweig (2011). "Sequence and structural convergence of broad and potent HIV antibodies that mimic CD4 binding." Science **333**(6049): 1633-1637.

Shibata, R., T. Igarashi, N. Haigwood, A. Buckler-White, R. Ogert, W. Ross, R. Willey, M. W. Cho and M. A. Martin (1999). "Neutralizing antibody directed against the HIV-1 envelope glycoprotein can completely block HIV-1/SIV chimeric virus infections of macaque monkeys." Nat. Med. **5**: 204-210.

Shingai, M., O. K. Donau, R. J. Phishka, A. Buckler-White, J. R. Mascola, G. J. Nabel and E. al. (2014). "Passive transfer of modest titers of potent and broadly neutralizing anti-HIV monoclonal anitbodies block SHIV infection in macaques." Journal of Experimental Medicine **211**(10).:2061-2074.

Shingai, M., Y. Nishimura, F. Klein, H. Mouquet, O. K. Donau, R. Plishka, A. Buckler-White, M. Seaman, M. Piatak, J. D. Lifson, D. Dimitrov, M. C. Nussenzweig and M. A. Martin (2013). "Antibody-mediated immunotherapy of macaques chronically infected with SHIV suppresses viraemia." Nature.

Simek, M. D., W. Rida, F. H. Priddy, P. Pung, E. Carrow, D. S. Laufer, J. K. Lehrman, M. Boaz, T. Tarragona-Fiol, G. Miiro, J. Birungi, A. Pozniak, D. A. McPhee, O. Manigart, E. Karita, A. Inwoley, W. Jaoko, J. Dehovitz, L. G. Bekker, P. Pitisuttithum, R. Paris, L. M. Walker, P. Poignard, T. Wrin, P. E. Fast, D. R. Burton and W. C. Koff (2009). "Human immunodeficiency virus type 1 elite neutralizers: individuals with broad and potent neutralizing activity identified by using a high-throughput neutralization assay together with an analytical selection algorithm." J. Virol. **83**(14): 7337-7348.

Thigpen, M. C., P. M. Kebaabetswe, L. A. Paxton, D. K. Smith, C. E. Rose, T. M. Segolodi, F. L. Henderson, S. R. Pathak, F. A. Soud, K. L. Chillag, R. Mutanhaurwa, L. I. Chirwa, M. Kasonde, D. Abebe, E. Buliva, R. J. Gvetadze, S. Johnson, T. Sukalac, V. T. Thomas, C. Hart, J. A. Johnson, C. K. Malotte, C. W. Hendrix and J. T. Brooks (2012). "Antiretroviral preexposure prophylaxis for heterosexual HIV transmission in Botswana." N. Engl. J. Med. **367**(5): 423-434.

Trkola, A., H. Kuster, P. Rusert, B. Joos, M. Fischer, C. Leemann, A. Manrique, M. Huber, M. Rehr, A. Oxenius, R. Weber, G. Stiegler, B. Vcelar, H. Katinger, L. Aceto and H. F. Gunthard (2005). "Delay of HIV-1 rebound after cessation of antiretroviral therapy through passive transfer of human neutralizing antibodies." Nat Med **11**(6): 615-622.

UNAIDS "AIDS by the NUmbers 2015." Accessed at http://www.unaids.org/en/resources/documents/2015/AIDS_by_the_numbers_2015.

Wagh K, Bhattacharya T, et al. (2016) “Optimal Combinations of Broadly Neutralizing Antibodies for Prevention and Treatment of HIV-1 Clade C Infection”. PLoS Pathog. **12**(3):e1005520.

West, A. P., Jr., L. Scharf, J. F. Scheid, F. Klein, P. J. Bjorkman and M. C. Nussenzweig (2014). "Structural insights on the role of antibodies in HIV-1 vaccine and therapy." Cell **156**(4): 633-648.
