# Supplementary material for: Safety, pharmacokinetics, and immunogenicity of the combination of the broadly neutralizing anti-HIV-1 antibodies 3BNC117 and 10-1074 in healthy adults: A randomized, phase 1 study
Source: PLoS One. 2019 Aug 8;14(8):e0219142. doi: 10.1371/journal.pone.0219142 (PMC6687118; doi:10.1371/journal.pone.0219142)
Supplement: S1 Table — (PDF) [file pone.0219142.s004.pdf]

Supplementary Table 1. Individual participant demographics

Group	Study Assignment	Participant ID	Age	Gender	Race	Ethnicity	BMI
**Group 1**	3BNC117+10-1074	1111	63	M	White	Non-hispanic	28
	3BNC117+10-1074	1172	37	M	White	Hispanic	27
	3BNC117+10-1074	1249	41	M	White	Non-hispanic	21
	Placebo	1347	34	M	White	Non-hispanic	30
	3BNC117+10-1074	1411	48	M	Black	Non-hispanic	31
	3BNC117+10-1074	1472	52	M	White	Hispanic	36
	3BNC117+10-1074	1568	54	M	Black	Non-hispanic	32
	Placebo	1628	42	M	Black	Non-hispanic	30
**Group 2**	3BNC117+10-1074	2378	54	M	Black	Non-hispanic	32
	3BNC117+10-1074	2575	41	M	Black	Non-hispanic	22
	Placebo	2589	43	F	Multiple	Non-hispanic	44
	3BNC117+10-1074	2639	50	M	Black	Non-hispanic	23
	3BNC117+10-1074	2754	34	F	Multiple	Hispanic	21
	3BNC117+10-1074	2798	42	M	Black	Non-hispanic	35
	Placebo	2873	40	M	Black	Non-hispanic	29
	3BNC117+10-1074	2993	24	M	Black	Hispanic	23
**Group 3**	3BNC117+10-1074	3158	29	M	Black	Non-hispanic	21
	3BNC117+10-1074	3180	33	M	White	Non-hispanic	23
	3BNC117+10-1074	3249	21	M	Black	Non-hispanic	38
	3BNC117+10-1074	3388	30	M	Black	Non-hispanic	25
	Placebo	3650	29	F	Black/His	HIspanic	23
	3BNC117+10-1074	3670	45	F	Black	Non-hispanic	27
	3BNC117+10-1074	3794	53	M	White	Hispanic	27
	Placebo	3892	53	F	White	Non-hispanic	19
