# Supplementary material for: Safety, pharmacokinetics, and immunogenicity of the combination of the broadly neutralizing anti-HIV-1 antibodies 3BNC117 and 10-1074 in healthy adults: A randomized, phase 1 study
Source: PLoS One. 2019 Aug 8;14(8):e0219142. doi: 10.1371/journal.pone.0219142 (PMC6687118; doi:10.1371/journal.pone.0219142)
Supplement: S2 Table — (PDF) [file pone.0219142.s005.pdf]

Supplementary Table 2. 3BNC117 and 10-1074 Serum Levels

Group 1
Single infusion, day 0
3BNC117 + 10-1074 (10 mg/kg each i.v.)

**Participant ID**	**3BNC117**		**10-1074**	
**1111**	***ELISA***	***TZM-bl***	***ELISA***	***TZM-bl***
		Q769.d22		*X2088_c9*
Study visit	*μg/ml*		*μg/ml*	
Day 0 end 3BNC117	189.18	166.79	-	-
Day 0 end 10-1074	-	-	501.96	409.53
Day 2	37.62	19.42	303.81	142.99
Week 1	22.27	18.06	176.31	124.04
Week 2	17.04	9.46	129.23	72.28
Week 4	8.47	2.52	69.12	33.38
Week 8	1.87	0.56	26.32	7.29
Week 12	0.86	0.28	11.72	2.04
Week 16	0.39	0.28	5.57	1.04
Week 20	0.39	0.28	2.19	0.29
Week 24	0.39	0.28	1.13	0.16

**Participant ID**	**3BNC117**		**10-1074**	
**1172**	***ELISA***	***TZM-bl***	***ELISA***	***TZM-bl***
		Q769.d22		*X2088_c9*
Study visit	*μg/ml*		*μg/ml*	
Day 0 end 3BNC117	219.22	87.91	-	-
Day 0 end 10-1074	-	-	458.06	316.52
Day 2	73.01	34.91	292.15	218.73
Week 1	38.41	21.29	203.77	106.66
Week 2	24.53	17.09	138.13	86.14
Week 4	14.39	7.97	106.76	53.17
Week 8	5.84	2.42	-	-
Week 12	-	-	-	-
Week 16	-	-	-	-
Week 20	-	-	-	-
Week 24	-	-	-	-

**Participant ID**	**3BNC117**		**10-1074**	
**1249**	***ELISA***	***TZM-bl***	***ELISA***	***TZM-bl***
		Q769.d22		*X2088_c9*
Study visit	*μg/ml*		*μg/ml*	
Day 0 end 3BNC117	238.17	113.63	-	-
Day 0 end 10-1074	-	-	540.91	391.19
Day 2	74.42	47.36	343.35	267.22
Week 1	32.09	15.02	209.95	131.05
Week 2	21.40	6.67	116.58	66.32
Week 4	9.65	5.83	77.19	29.67
Week 8	2.76	1.18	31.45	10.24
Week 12	1.80	0.28	13.98	3.10
Week 16	0.35	0.28	6.69	1.78
Week 20	0.39	0.28	3.52	0.43
Week 24	0.39	0.28	0.91	0.41

**Participant ID**	**3BNC117**		**10-1074**	
**1411**	***ELISA***	***TZM-bl***	***ELISA***	***TZM-bl***
		Q769.d22		*X2088_c9*
Study visit	*μg/ml*		*μg/ml*	
Day 0 end 3BNC117	246.54	160.63	-	-
Day 0 end 10-1074	-	-	700.43	1,353.77
Day 2	59.70	36.71	341.62	196.15
Week 1	31.15	25.16	205.29	133.55
Week 2	22.56	12.49	177.53	90.69
Week 4	14.91	5.74	103.57	44.71
Week 8	5.03	2.24	42.27	15.48
Week 12	1.58	0.28	19.07	5.89
Week 16	0.62	0.28	8.04	2.42
Week 20	0.64	0.28	2.63	0.33
Week 24	0.39	0.28	1.60	0.15

**Participant ID**	**3BNC117**		**10-1074**	
**1472**	***ELISA***	***TZM-bl***	***ELISA***	***TZM-bl***
		Q769.d22		*X2088_c9*
Study visit	*μg/ml*		*μg/ml*	
Day 0 end 3BNC117	325.89	172.33	-	-
Day 0 end 10-1074	-	-	727.43	392.85
Day 2	77.50	51.32	364.58	250.69
Week 1	38.70	25.62	266.09	200.09
Week 2	21.35	13.28	156.74	103.46
Week 4	13.75	9.85	96.38	68.00
Week 8	5.03	1.18	31.24	13.53
Week 12	1.65	0.28	12.21	5.28
Week 16	0.85	0.28	6.23	1.92
Week 20	0.39	0.28	2.57	0.38
Week 24	0.39	0.28	1.26	0.16

**Participant ID**	**3BNC117**		**10-1074**	
**1568**	***ELISA***	***TZM-bl***	***ELISA***	***TZM-bl***
		Q769.d22		*X2088_c9*
Study visit	*μg/ml*		*μg/ml*	
Day 0 end 3BNC117	727.43	196.74	-	-
Day 0 end 10-1074	-	-	727.43	671.98
Day 2	364.58	29.90	364.58	241.17
Week 1	266.09	18.40	266.09	116.30
Week 2	156.74	13.21	156.74	98.23
Week 4	96.38	9.96	96.38	54.02
Week 8	31.24	1.02	31.24	10.76
Week 12	12.21	0.28	12.21	2.88
Week 16	6.23	0.28	6.23	1.43
Week 20	2.57	0.28	2.57	0.37
Week 24	1.26	0.28	1.26	0.19

Supplementary Table 2, *continued*

Group 2
Repeated infusions, day 0, wk 8, wk 16
3BNC117 + 10-1074 (3 mg/kg each i.v.)

Participant ID	3BNC117		10-1074	
**2378**	*ELISA*	*TZM-bl*	*ELISA*	*TZM-bl*
		Q769.d22		*X2088_c9*
Study visit	*μg/ml*		*μg/ml*	
Day 0 end 3BNC117	69.05	56.784	-	-
Day 0 end 10-1074	-	-	207.76	73.984
Day 2	15.243		102.331	
Week 1	8.532	6.72	69.114	37.232
Week 2	4.449	2.072	33.541	29.456
Week 4	1.383	0.28	14.704	13.644
Week 8	4.348	0.28	4.348	4.364
Wk 8 end 3BNC117	54.121	33.012	-	-
Wk 8 end 10-1074	-	-	164.207	74.008
Day 58	12.133	-	87.398	-
Week 9	-	-		44.4
Week 10	2.47	1.708	23.626	26.196
Week 12	1.355	0.812	14.993	16.364
Week 16	0	0.28	4.776	5.304
Wk 16 end	93.095	28.041	-	-
Wk 16 end 10-1074	-	-	79.639	52.785
Day 114	15.273	-	46.729	-
Week 17	9.86	8.32	40.634	24.363
Week 18	4.107	2.652	23.108	17.199
Week 20	2.392	1.092	12.023	9.144
Week 24	0.78	0.28	3.911	3.966
Week 28	0.39	0.28	2.153	1.782
Week 32	0.39	0.28	0.463	1.41
Week 36	0.39	0.28	0.412	0.768
Week 40	0.39	0.28	0.412	0.372

Participant ID	3BNC117		10-1074	
**2575**	*ELISA*	*TZM-bl*	*ELISA*	*TZM-bl*
		Q769.d22		*X2088_c9*
Study visit	*μg/ml*		*μg/ml*	
Day 0 end 3BNC117	54.527	23.254	-	-
Day 0 end 10-1074	-	-	158.656	72.204
Day 2	16.237	-	97.136	-
Week 1	6.197	3.43	50.825	16.264
Week 2	4.484	2.366	36.586	9.74
Week 4	2.293	0.84	23.548	5.66
Week 8	0.46	0.28	7.314	1.872
Wk 8 end 3BNC117	86.973	-	-	-
Wk 8 end 10-1074	-	-	-	-
Day 58	15.499	-	-	-
Week 9	8.8	4.284	-	-
Week 10	5.645	3.066	-	-
Week 12	2.458	1.526	-	-
Week 16	0.78	-	-	-
Wk 16 end	-	-	-	-
Wk 16 end 10-1074	-	-	-	-
Day 114	-	-	-	-
Week 17	0.78		-	-
Week 18	-	-	-	-
Week 20	-	-	-	-
Week 24	-	-	-	-
Week 28	-	-	-	-
Week 32	-	-	-	-
Week 36	-	-	-	-
Week 40	-	-	-	-

Participant ID	3BNC117		10-1074	
**2639**	*ELISA*	*TZM-bl*	*ELISA*	*TZM-bl*
		Q769.d22		*X2088_c9*
Study visit	*μg/ml*		*μg/ml*	
Day 0 end 3BNC117	57.544	50.652	-	-
Day 0 end 10-1074	-	-	166.485	91.876
Day 2	30.472		116.492	
Week 1	8.192	4.158	57.367	23.864
Week 2	5.559	3.108	45.851	32.624
Week 4	2.481	1.54	25.797	6.524
Week 8	0.866	0.28	8.413	1.632
Wk 8 end 3BNC117	55.629	41.692	-	-
Wk 8 end 10-1074	-	-	197.532	141.224
Day 58	24.729	-	132.721	-
Week 9	10.117	11.018	60.265	35.608
Week 10	5.543	9.772	48.164	19.536
Week 12	2.666	2.842	32.226	11.448
Week 16	0.78	0.602	6.379	3.024
Wk 16 end	67.82	67.808	-	-
Wk 16 end 10-1074	-	-	101.291	57.762
Day 114	25.888	-	57.173	-
Week 17	11.822	6.292	43.333	18.444
Week 18	6.633	3.224	34.272	11.19
Week 20	2.804	1.313	16.339	4.359
Week 24	0.39	0.28	6.35	1.173
Week 28	0.39	0.28	6.183	0.474
Week 32	0.39	0.28	1.106	0.414
Week 36	0.39	0.28	0.459	0.08
Week 40	0.39	0.28	0.412	0.08

Participant ID	3BNC117		10-1074	
**2754**	*ELISA*	*TZM-bl*	*ELISA*	*TZM-bl*
		Q769.d22		*X2088_c9*
Study visit	*μg/ml*		*μg/ml*	
Day 0 end 3BNC117	26.054	-	-	-
Day 0 end 10-1074	-	-	-	-
Day 2	9.745	-	59.948	-
Week 1	7.26	2.604	57.671	29.404
Week 2	5.3	2.086	37.233	16.592
Week 4	2.399	0.742	22.344	7.82
Week 8	0.638	0.28	7.92	2.664
Wk 8 end 3BNC117	60.437	24.178	-	-
Wk 8 end 10-1074	-	-	191.314	67.088
Day 58	16.097	-	112.068	-
Week 9	-	-	-	-
Week 10	7.028	3.22	51.28	17
Week 12	2.73	1.008	25.583	7.06
Week 16	0.807	0.28	6.301	2.64
Wk 16 end	88.986	29.289	-	-
Wk 16 end 10-1074	-	-	78.882	43.791
Day 114	16.065	-	50.179	-
Week 17	10.942	5.629	38.482	11.82
Week 18	6.029	3.159	27.559	8.604
Week 20	2.689	1.131	15.985	3.669
Week 24	0.797	0.28	6.44	1.323
Week 28	0.39	0.28	4.295	0.492
Week 32	0.39	0.28	1.129	0.414
Week 36	0.39	0.28	0.443	0.15
Week 40	0.39	0.28	0.412	0.08

Participant ID	3BNC117		10-1074	
**2798**	*ELISA*	*TZM-bl*	*ELISA*	*TZM-bl*
		Q769.d22		*X2088_c9*
Study visit	*μg/ml*		*μg/ml*	
Day 0 end 3BNC117	26.054	58.324	-	-
Day 0 end 10-1074	-	-	179.81	73.984
Day 2	9.745	-	99.334	-
Week 1	7.26	10.654	97.905	37.232
Week 2	5.3	4.676	64.525	29.456
Week 4	2.399	2.842	40.316	13.644
Week 8	0.638	0.616	20.88	4.364
Wk 8 end 3BNC117	60.437	61.614	-	-
Wk 8 end 10-1074	-	-	198.381	74.008
Day 58	16.097	-	172.891	-
Week 9	-	8.834	111.547	44.4
Week 10	7.028	7.224	65.034	26.196
Week 12	2.73	5.432	50.394	16.364
Week 16	0.807	1.246	11.882	5.304
Wk 16 end	88.986	48.035	-	-
Wk 16 end 10-1074	-	-	111.432	52.785
Day 114	16.065	-	83.732	-
Week 17	10.942	13.598	52.99	24.363
Week 18	6.029	9.789	46.989	17.199
Week 20	2.689	3.367	40.08	9.144
Week 24	0.797	0.975	17.389	3.966
Week 28	0.39	0.28	9.438	1.782
Week 32	0.39	0.28	6.539	1.41
Week 36	0.39	0.28	2.148	0.768
Week 40	0.39	0.28	1.0024	0.372

Participant ID	3BNC117		10-1074	
**2993**	*ELISA*	*TZM-bl*	*ELISA*	*TZM-bl*
		Q769.d22		*X2088_c9*
Study visit	*μg/ml*		*μg/ml*	
Day 0 end 3BNC117	57.181	23.8	-	-
Day 0 end 10-1074	-	-	180.368	112.58
Day 2	15.136	-	113.705	-
Week 1	7.728	5.11	79.981	41.824
Week 2	7.073	3.5	53.586	25.996
Week 4	3.455	1.302	31.631	14.52
Week 8	1.1	0.28	17.158	5.008
Wk 8 end 3BNC117	50.066	45.136	-	-
Wk 8 end 10-1074	-	-	175.777	112.404
Day 58	17.952	-	113.607	-
Week 9	9.628	8.624	73.658	30.188
Week 10	6.86	6.006	58.757	30.28
Week 12	4.095	2.842	42.488	15.34
Week 16	1.354	0.364	9.11	5.076
Wk 16 end	71.719	24.947	-	-
Wk 16 end 10-1074	-	-	73.262	35.649
Day 114	22.57	-	65.519	-
Week 17	13.3	5.941	43.977	23.319
Week 18	7.801	3.562	35.774	14.115
Week 20	4.394	1.794	21.894	7.098
Week 24	1.329	0.507	9.594	2.628
Week 28	0.78	0.28	8.425	0.957
Week 32	0.39	0.28	2.024	0.798
Week 36	0.39	0.28	1.115	0.576
Week 40	0.39	0.28	0.592	0.288

Supplementary Table 2, *continued*

Group 3
Repeated infusions, day 0, wk 8, wk 16
3BNC117 + 10-1074 (10 mg/kg each i.v.)

Participant ID	3BNC117		10-1074	
**3158**	***ELISA***	***TZM-bl***	***ELISA***	***TZM-bl***
		Q769.d22		*X2088_c9*
Study visit	*µg/ml*		*µg/ml*	
Day 0 end 3BNC117	185.026	154.31	-	-
Day 0 end 10-1074	-	-	256.918	169.596
Day 2	59.329	-	195.758	-
Week 1	29.11	43.784	133.258	53.976
Week 2	19.718	11.453	106.577	45.081
Week 4	15.274	6.708	78.502	22.308
Week 8	5.681	5.343	44.421	11.259
Wk 8 end 3BNC117	302.088	215.995	361.451	160.893
Wk 8 end 10-1074	-	-	-	-
Day 58	80.091	-	275.849	-
Week 9	48.081	25.168	173.126	95.922
Week 10	39.559	17.277	137.289	48.378
Week 12	22.474	16.107	98.646	29.037
Week 16	11.619	5.491	88.843	29.01
Wk 16 end	292.955	276.76	-	
Wk 16 end 10-1074	-	-	479.776	410.718
Day 114	120.21	140.063	406.174	323.952
Week 17	48.465	46.903	237.556	132.024
Week 18	48.786	21.896	246.121	148.038
Week 20	23.553	17.153	167.118	56.148
Week 24	14.833	10.115	97.401	43.788
Week 28	5.999	4.029	58.195	18.144
Week 32	3.458	1.666	39.007	17.556
Week 36	1.726	0.935	24.497	5.898
Week 40	0.924	0.28	11.987	5.892

Participant ID	3BNC117		10-1074	
**3180**	***ELISA***	***TZM-bl***	***ELISA***	***TZM-bl***
		Q769.d22		*X2088_c9*
Study visit	*µg/ml*		*µg/ml*	
Day 0 end 3BNC117	242.64	139.542	314.627	
Day 0 end 10-1074	-	-	214.046	214.596
Day 2	78.432		120.506	
Week 1	32.093	15.08	105.301	54.78
Week 2	21.228	13.442	47.071	32.058
Week 4	13.95	5.135	22.986	17.646
Week 8	3.119	1.196	380.24	4.98
Wk 8 end 3BNC117	299.722	125.268		187.437
Wk 8 end 10-1074	-	-	184.48	
Day 58	50.837		132.33	
Week 9	34.288	25.467	91.184	47.19
Week 10	24.752	14.378	52.887	30.501
Week 12	11.512	5.07	30.223	14.208
Week 16	3.755	2.635	-	11.46
Wk 16 end	338.408	286.229	-	-
Wk 16 end 10-1074	-	-	463.529	587.826
Day 114	82.078	111.129	278.012	129.126
Week 17	42.288	51.289	185.744	93.744
Week 18	35.221	17.901	148.814	69.12
Week 20	14.268	11.237	70.805	37.992
Week 24	6.128	5.355	37.833	16.098
Week 28	1.571	0.459	11.032	5.148
Week 32	0.78	0.28	9.073	3.492
Week 36	0.39	0.28	4.121	1.302
Week 40	0.39	0.28	1.129	0.468

Participant ID	3BNC117		10-1074	
**3249**	***ELISA***	***TZM-bl***	***ELISA***	***TZM-bl***
		Q769.d22		*X2088_c9*
Study visit	*µg/ml*		*µg/ml*	
Day 0 end 3BNC117	391.973	162.825	-	-
Day 0 end 10-1074	-	-	453.853	197.532
Day 2	60.526		206.985	
Week 1	46.699	28.639	149.859	76.776
Week 2	34.694	35.035	114.346	36.93
Week 4	13.992	27.469	61.973	19.527
Week 8	3.118	1.729	24.315	7.008
Wk 8 end 3BNC117	344.269	196.807	-	-
Wk 8 end 10-1074	-	-	426.266	213.231
Day 58	105.215	-	307.57	-
Week 9	44.122	35.347	175.889	71.55
Week 10	27.792	27.664	142.292	51.729
Week 12	15.741	7.371	75.927	34.692
Week 16	5.936	1.462	38.799	8.154
Wk 16 end	403.889	145.129	-	-
Wk 16 end 10-1074	-	-	525.111	321.954
Day 114	128.323	85.646	384.415	180.984
Week 17	55.904	26.996	285.786	105.726
Week 18	38.878	21.828	175.835	79.974
Week 20	19.456	7.446	99.712	42.996
Week 24	5.789	2.176	44.82	12.816
Week 28	1.718	0.28	10.102	5.442
Week 32	0.39	0.28	5.504	2.544
Week 36	0.39	0.28	2.304	1.278
Week 40	0.39	0.28	0.992	0.51

Participant ID	3BNC117		10-1074	
**3388**	***ELISA***	***TZM-bl***	***ELISA***	***TZM-bl***
		Q769.d22		*X2088_c9*
Study visit	*µg/ml*		*µg/ml*	
Day 0 end 3BNC117	368.793	205.153	-	-
Day 0 end 10-1074	-	-	360.652	225.462
Day 2	65.737		204.503	
Week 1	33.813	20.501	125.622	48.132
Week 2	18.685	10.673	93.092	24.219
Week 4	6.041	15.665	34.292	7.233
Week 8	1.152	2.678	9.189	2.43
Wk 8 end 3BNC117	406.332	132.639	-	-
Wk 8 end 10-1074	-	-	443.444	174.027
Day 58	57.391	-	176.304	-
Week 9	36.914	63.414	133.324	69.09
Week 10	20.891	33.423	74.967	27.936
Week 12	4.443	12.987	27.481	7.191
Week 16	0.78	1.139	9.289	2.184
Wk 16 end	337.658	194.922	-	-
Wk 16 end 10-1074	-	-	468.816	233.574
Day 114	67.187	65.178	301.199	248.844
Week 17	24.336	35.054	156.673	191.772
Week 18	18.353	12.988	126.272	82.032
Week 20	4.947	9.86	49.391	17.52
Week 24	0.959	1.479	12.231	3.6
Week 28	0.39	0.28	4.506	0.972
Week 32	0.39	2.584	1.027	0.246
Week 36	0.39	19.142	0.428	0.08
Week 40	0.39	2.856	0.412	0.08

Participant ID	3BNC117		10-1074	
**3670**	***ELISA***	***TZM-bl***	***ELISA***	***TZM-bl***
		Q769.d22		*X2088_c9*
Study visit	*µg/ml*		*µg/ml*	
Day 0 end 3BNC117	372.061	189.332	-	-
Day 0 end 10-1074	-	-	395.755	335.268
Day 2	115.153	-	309.098	-
Week 1	50.399	32.578	145.597	80.988
Week 2	33.684	38.727	127.102	45.255
Week 4	18.256	14.274	64.245	20.064
Week 8	2.907	1.417	19.53	4.626
Wk 8 end 3BNC117	329.227	207.935	-	-
Wk 8 end 10-1074	-	-	418.328	369.12
Day 58	123.771	-	384.567	-
Week 9	51.778	52.156	229.553	122.709
Week 10	43.075	31.512	133.08	51.351
Week 12	15.796	11.505	78.17	46.89
Week 16	4.35	2.516	42.556	15.84
Wk 16 end	498.354	370.872	-	-
Wk 16 end 10-1074	-	-	590.106	493.92
Day 114	127.125	149.039	513.513	330.294
Week 17	53.175	43.01	238.74	123.462
Week 18	37.175	20.281	119.979	88.752
Week 20	18.694	8.619	48.959	38.556
Week 24	3.986	2.618		13.368
Week 28	1.228	0.68	24.118	5.628
Week 32	0.39	0.28	6.144	2.424
Week 36	0.39	0.28	3.018	1.392
Week 40	0.39	0.28	1.203	0.57

Participant ID	3BNC117		10-1074	
**3794**	***ELISA***	***TZM-bl***	***ELISA***	***TZM-bl***
		Q769.d22		*X2088_c9*
3,794.00	*µg/ml*		*µg/ml*	
Day 0 end 3BNC117	288.678	295.568		
Day 0 end 10-1074			356.54	199.518
Day 2	79.604		226.166	
Week 1	37.847	51.038	150.303	78.411
Week 2	20.891	13.871	97.706	59.202
Week 4	14.594	6.643	56.395	22.251
Week 8	2.76	2.158	20.788	5.493
Wk 8 end 3BNC117	366.437	185.796	-	-
Wk 8 end 10-1074	-	-	345.16	156.771
Day 58	99.522	-	286.451	-
Week 9	32.573	16.133	148.493	46.386
Week 10	21.151	14.365	111.632	38.925
Week 12	10.97	5.902	75.959	17.304
Week 16	3.71	1.394	34.417	12.252
Wk 16 end	351.904	205.649	-	-
Wk 16 end 10-1074	-	-	548.374	315.678
Day 114	88.814	53.465	278.431	182.286
Week 17	60.867	29.631	226.242	94.806
Week 18	29.458	8.058	124.682	55.218
Week 20	18.712	5.423	109.701	32.484
Week 24	4.177	1.309	10.683	12.288
Week 28	1.214	0.425		4.95
Week 32	0.39	0.28	5.537	2.046
Week 36	0.39	0.28	2.881	0.9
Week 40	0.39	0.28	1.29	0.432
