# Supplementary material for: Safety, pharmacokinetics, and immunogenicity of the combination of the broadly neutralizing anti-HIV-1 antibodies 3BNC117 and 10-1074 in healthy adults: A randomized, phase 1 study
Source: PLoS One. 2019 Aug 8;14(8):e0219142. doi: 10.1371/journal.pone.0219142 (PMC6687118; doi:10.1371/journal.pone.0219142)
Supplement: S3 Table — (PDF) [file pone.0219142.s006.pdf]

Supplementary Table 3. Pharmacokinetic paramenters of 3BNC117 and 10-1074 administered in combination

Study Group	3BNC117						10-1074				
	Participant ID	Clearance (L/day)	Cmax (µg/ml)	AUC (µg x hr/ml)	Distribution $t_{1/2}$ (days)	Elimination $t_{1/2}$ (days)	Clearance (L/day)	Cmax (µg/ml)	AUC (µg x hr/ml)	Distribution $t_{1/2}$ (days)	Elimination $t_{1/2}$ (days)
Group 1	1111	1.06	189.18	808.42	0.86	15.75	0.14	501.96	6139.79	2.49	23.65
	1172	0.62	219.22	1293.31	0.39	18.55	0.10	458.06	8072.65	0.52	24.28
	1249	0.64	238.17	1029.00	0.40	17.53	0.10	540.91	6659.94	2.63	24.17
	1411	0.85	263.45	1249.12	0.67	23.29	0.10	700.43	10696.27	0.84	21.08
	1472	0.69	350.54	1533.99	1.11	18.55	0.11	723.71	9499.06	2.89	22.98
	1568	0.66	362.96	1311.72	0.61	16.65	0.10	727.43	8678.10	4.22	23.03
Group 2	2378	1.08	93.10	778.57	0.74	9.03	0.18	207.76	4613.12	3.18	16.86
	2575	0.75	86.97	503.79	0.49	11.10	0.10	158.66	1811.04	1.55	20.10
	2639	0.82	67.82	835.24	0.65	13.90	0.13	197.53	5299.45	0.89	19.64
	2754	0.58	88.99	793.93	0.51	13.57	0.09	191.31	4872.15	1.06	19.54
	2798	0.55	121.67	1786.92	0.69	21.18	0.11	198.38	9123.88	2.22	25.14
	2993	0.76	71.72	1005.35	0.74	16.41	0.11	180.37	6928.75	1.84	22.95
Group 3	3158	0.44	302.09	5028.39	0.43	27.99	0.08	479.78	26875.78	1.34	42.10
	3180	0.52	338.41	3661.68	0.54	17.12	0.12	463.53	15470.15	3.32	25.51
	3249	0.73	403.89	4556.03	0.40	15.54	0.17	525.11	19294.28	0.90	20.91
	3388	0.93	406.33	2755.95	0.65	11.05	0.21	468.82	11945.47	4.28	16.96
	3670	0.46	498.35	4585.45	0.38	13.63	0.10	590.11	21997.13	3.54	21.44
	3794	0.59	366.44	4141.07	0.71	14.50	0.14	548.37	17167.98	2.31	23.05
