# Supplementary material for: Safety, pharmacokinetics, and immunogenicity of the combination of the broadly neutralizing anti-HIV-1 antibodies 3BNC117 and 10-1074 in healthy adults: A randomized, phase 1 study
Source: PLoS One. 2019 Aug 8;14(8):e0219142. doi: 10.1371/journal.pone.0219142 (PMC6687118; doi:10.1371/journal.pone.0219142)
Supplement: S4 Table — (PDF) [file pone.0219142.s007.pdf]

Supplementary Table 4. Serum neutralizing activity against a multi-clade pseudovirus panel in group 2 participants

Pseudovirus (clade)	Participant ID					
	**2993**	**2639**	**2378**	**2575**	**2798**	**2754**
	ID50 titer	*ID50 titer*	*ID50 titer*	*ID50 titer*	*ID50 titer*	*ID50 titer*
**SC422661.8 (B)**	298	426	308	254	675	459
**RHPA4259.7 (B)**	784	995	982	627	1684	1186
**Du156.12 (C)**	759	758	533	591	866	945
**ZM135M.PL10a (C)**	133	112	98	98	308	198
**CNE53 (BC)**	609	621	436	449	1023	501
**191084 B7-19 (A)**	248	295	166	160	430	259
**263-8 (CRF02_AG)**	95	118	46	65	180	95
**235-47 (CRF02_AG)**	295	293	177	209	396	206
**X1254_c3 (G)**	215	214	92	115	235	150
**A07412M1.vrc12 (D)**	1666	1436	857	1131	1764	1192
**0815.v3.c3 (ACD)**	833	1310	1005	558	2055	1658
**3301.v1.c24 (AC)**	1264	1753	753	939	1335	852
