# Supplementary material for: Safety, pharmacokinetics, and immunogenicity of the combination of the broadly neutralizing anti-HIV-1 antibodies 3BNC117 and 10-1074 in healthy adults: A randomized, phase 1 study
Source: PLoS One. 2019 Aug 8;14(8):e0219142. doi: 10.1371/journal.pone.0219142 (PMC6687118; doi:10.1371/journal.pone.0219142)
Supplement: S5 Table — (PDF) [file pone.0219142.s008.pdf]

Supplementary Table 5. Anti-drug antibody responses

	Participant ID	Time point	3BNC117			10-1074		
			Immunogenicity Assay Results	Specificty Assay Results (Specific/Non-specific)	Titer	Immunogenicity Assay Results	Specificty Assay Results (Specific/Non-specific)	Titer
Group 1	1172	Day 0	Negative	-	-	Negative	-	-
		Week 8	Negative	-	-	Negative	-	-
	1249	Day 0	Negative	-	-	Negative	-	-
		Week 8	Negative	-	-	Negative	-	-
		Week 24	Negative	-	-	Negative	-	-
	1568	Day 0	Negative	-	-	Negative	-	-
		Week 8	Positive	Specific	27.00	Negative	-	-
		Week 24	Positive	Specific	3.00	Negative	-	-
	1472	Day 0	Negative	-	-	Negative	-	-
		Week 8	Negative	-	-	Negative	-	-
		Week 24	Negative	-	-	Negative	-	-
	1411	Day 0	Positive	Non-specific	-	Negative	-	-
		Week 8	Negative	-	-	Negative	-	-
		Week 24	Negative	-	-	Negative	-	-
	1111	Day 0	Positive	Specific	3.00	Negative	-	-
		Week 8	Positive	Specific	1.00	Negative	-	-
		Week 24	Positive	Specific	3.00	Negative	-	-
Group 2	2754	Day 0	Negative	-	-	Negative	-	-
		Week 8	Negative	-	-	Negative	-	-
		Week 16	Negative	-	-	Negative	-	-
		Week 24	Negative	-	-	Negative	-	-
		Week 36	Negative	-	-	Negative	-	-
	2798	Day 0	Negative	-	-	Negative	-	-
		Week 8	Negative	-	-	Negative	-	-
		Week 16	Negative	-	-	Negative	-	-
		Week 24	Negative	-	-	Negative	-	-
		Week 36	Negative	-	-	Negative	-	-
	2575	Day 0	Negative	-	-	Negative	-	-
		Week 8	Negative	-	-	Negative	-	-
		Week 16	Negative	-	-	Negative	-	-
		Week 24	Negative	-	-	Negative	-	-
		Week 36	Negative	-	-	Negative	-	-
	2378	Day 0	Negative	-	-	Negative	-	-
		Week 8	Negative	-	-	Negative	-	-
		Week 16	Negative	-	-	Negative	-	-
		Week 24	Negative	-	-	Negative	-	-
		Week 36	Negative	-	-	Negative	-	-
	2639	Day 0	Negative	-	-	Negative	-	-
		Week 8	Negative	-	-	Negative	-	-
		Week 16	Negative	-	-	Negative	-	-
		Week 24	Negative	-	-	Positive	Specific	1.00
		Week 36	Negative	-	-	Positive	Specific	1.00
	2993	Day 0	Negative	-	-	Negative	-	-
		Week 8	Negative	-	-	Negative	-	-
		Week 16	Negative	-	-	Negative	-	-
		Week 24	Negative	-	-	Negative	-	-
		Week 36	Negative	-	-	Negative	-	-
Group 3	3180	Day 0	Positive	Specific	81.00	Negative	-	-
		Week 8	Negative	-	-	Negative	-	-
		Week 16	Negative	-	-	Negative	-	-
		Week 24	Negative	-	-	Negative	-	-
		Week 36	Positive	Specific	3.00	Negative	-	-
	3249	Day 0	Positive	Non-specific	-	Negative	-	-
		Week 8	Negative	-	-	Negative	-	-
		Week 16	Negative	-	-	Negative	-	-
		Week 24	Negative	-	-	Negative	-	-
		Week 36	Negative	-	-	Negative	-	-
	3794	Day 0	Negative	-	-	Negative	-	-
		Week 8	Negative	-	-	Negative	-	-
		Week 16	Negative	-	-	Negative	-	-
		Week 24	Negative	-	-	Negative	-	-
		Week 36	Negative	-	-	Negative	-	-
	3388	Day 0	Negative	-	-	Negative	-	-
		Week 8	Negative	-	-	Negative	-	-
		Week 16	Negative	-	-	Negative	-	-
		Week 24	Negative	-	-	Negative	-	-
		Week 36	Negative	-	-	Negative	-	-
	3158	Day 0	Negative	-	-	Negative	-	-
		Week 8	Negative	-	-	Negative	-	-
		Week 16	Negative	-	-	Negative	-	-
		Week 24	Negative	-	-	Negative	-	-
		Week 36	Negative	-	-	Negative	-	-
	3670	Day 0	Negative	-	-	Negative	-	-
		Week 8	Negative	-	-	Negative	-	-
		Week 16	Negative	-	-	Negative	-	-
		Week 24	Negative	-	-	Negative	-	-
		Week 36	Negative	-	-	Negative	-	-
