# Supplementary material for: Safety, pharmacokinetics, and immunogenicity of the combination of the broadly neutralizing anti-HIV-1 antibodies 3BNC117 and 10-1074 in healthy adults: A randomized, phase 1 study
Source: PLoS One. 2019 Aug 8;14(8):e0219142. doi: 10.1371/journal.pone.0219142 (PMC6687118; doi:10.1371/journal.pone.0219142)
Supplement: S2 File — (PDF) [file pone.0219142.s002.pdf]

Institutional Review Board
Emil C. Gotschlich, MD, Chair
Dale Miller, CIP
IRB Specialist (212) 327- 8411
Vanessa Smith, MPS, CIP
IRB Specialist (212) 327-8410
Hospital Bldg., Room 201 Box 331

The Rockefeller University — SCIENCE FOR THE BENEFIT OF HUMANITY

February 22, 2016

Yehuda Cohen, MD
Laboratory of Molecular Immunology
The Rockefeller University

RE: YCO-0899 A phase 1 open label study of the safety and pharmacokinetics of the combination of 3BNC117 and 10-1074 in HIV-uninfected adults.

Dear Dr. Cohen,

Thank you for submitting the modifications to documents for the above protocol (iRIS submission # 332441), as requested in our letter of **February 5, 2016**. On behalf of the board I have reviewed and approved the modifications.

This study was reviewed and granted stipulated Initial approval at the convened meeting of the Institutional Review Board (IRB) on February 4, 2016. The final approval period is **February 22, 2016 through February 3, 2017.** The following documents were reviewed:

Type	Document Name	Version	Date Submitted into Workflow
**Submission Response by Board:**			
Institutional Review Board	Review Response Submission Form	Version 1.0	02/20/2016 07:31 PM EST
**Submission Form:**			
Submission Form	Initial Review Submission Form	Version 1.1	02/20/2016 07:31 PM EST
Submission Form	Initial Review Submission Form	Version 1.0	01/26/2016 09:12 AM EST
**Submission Attachments:**			
Application	Study Application	Version 1.1	02/20/2016 07:31 PM EST
Application	Study Application	Version 1.0	01/26/2016 09:12 AM EST
Consent (English)	YCO_ICF_Comb_Uninfected_17Feb2016	Version	02/20/2016

Institutional Review Board
Emil C. Gotschlich, MD, Chair
Dale Miller, CIP
IRB Specialist (212) 327- 8411
Vanessa Smith, MPS, CIP
IRB Specialist (212) 327-8410
Hospital Bldg., Room 201 Box 331

Type	Document Name	Version	Date Submitted into Workflow
		1.1	07:31 PM EST
Consent	YCO_ICF_Comb_Uninfected_22JAN2016	Version 1.0	01/26/2016 09:12 AM EST
Document - Other Supporting Documentation	Pre-Screening Questionnaire_19Feb2016	Version 1.0	02/20/2016 07:31 PM EST
Document - Other Supporting Documentation	Lorenzi_CITI_Certification	Version 1.0	02/20/2016 07:31 PM EST
Document -	10-1074_IB_25Jan 2016_Clean	Version 1.0	01/26/2016 09:12 AM EST
Document -	YCO_AD_Initial_Submission	Version 1.0	01/26/2016 09:12 AM EST
Document -	yco_comb_table_2_pharmacokinetics	Version 1.0	01/26/2016 09:12 AM EST
Document -	yco_comb_uninfected_table_1_safetyassessments	Version 1.0	01/26/2016 09:12 AM EST
Document -	yco_comb_uninfected_figures_2-4	Version 1.0	01/26/2016 09:12 AM EST
Document -	yco_comb_uninfected_figure_1_studydesign	Version 1.0	01/26/2016 09:12 AM EST
Document -	YCO_CCTS_Report_Initial_Submission	Version 1.0	01/26/2016 09:12 AM EST
Document -	YCO_Comb_Uninfected_HIPAA_Initial_Submission	Version 1.0	01/26/2016 09:12 AM EST
Document -	3BNC117_IB_version2.0_9March2015_clean	Version 1.0	01/26/2016 09:12 AM EST

SCIENCE FOR THE BENEFIT OF HUMANITY

Institutional Review Board
Emil C. Gotschlich, MD, Chair
Dale Miller, CIP
IRB Specialist (212) 327- 8411
Vanessa Smith, MPS, CIP
IRB Specialist (212) 327-8410
Hospital Bldg., Room 201 Box 331

Type	Document Name	Version	Date Submitted into Workflow
Document - Appendix to Protocol	yco_appendix b_toxicity tables	Version 1.0	01/26/2016 09:12 AM EST
Document -	YCO_Protocol_3BNC117 + 10-1074 HIV negative _17Feb16 CLEAN	Version 1.1	02/20/2016 07:31 PM EST
Document -	YCO_Protocol_3BNC117 + 10-1074 HIV negative _23JAN16 CLEAN	Version 1.0	01/26/2016 09:12 AM EST
Document - Other Supporting Documentation	YCO_Figure_5_IT flowchart_16Feb16	Version 1.1	02/20/2016 07:31 PM EST
Document - Other Supporting Documentation	YCO_Figure_5_IT flowchart	Version 1.0	01/26/2016 09:12 AM EST
Document -	YCO_Appendix A_Comb_Uninfected_Time_of_Events_17Feb16	Version 1.1	02/20/2016 07:31 PM EST
Document -	YCO_Appendix A_Comb_Uninfected_Time_of_Events_25JAN16	Version 1.0	01/26/2016 09:12 AM EST

The number assigned to this protocol **is YCO-0899.**

Please note that submission for Continuing Review of this protocol is due no later than 9:30 am **on December 20, 2016.**

****Enrollment may not begin until The Rockefeller University IRB acknowledges receipt of FDA approval for this study.**

Institutional Review Board
Emil C. Gotschlich, MD, Chair
Dale Miller, CIP
IRB Specialist (212) 327- 8411
Vanessa Smith, MPS, CIP
IRB Specialist (212) 327-8410
Hospital Bldg., Room 201 Box 331

SCIENCE FOR THE BENEFIT OF HUMANITY

It is the Principal Investigator's responsibility, as required by Federal regulations, to (1) Submit any proposed changes in approved studies to the IRB for review and approval prior to initiation, except where necessary to eliminate apparent immediate hazards to the subjects; (2) To promptly inform the IRB, appropriate institutional officials, the Office for Human Research Protections (OHRP) and the FDA, if applicable of any unanticipated problems involving risks to subjects or others and research related injuries; (3) To obtain the informed consent of the participant in the manner and format approved; and (4) To resubmit to the IRB for continuing review at the interval determined by the IRB to be appropriate to the risk, but not less than once a year.

Sincerely yours,

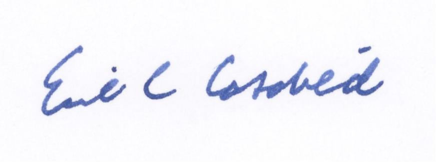

Signature applied by Emil C Gotschlich on 02/23/2016 12:08:27 PM EST

Emil C. Gotschlich, MD
Chair, Institutional Review Board
The Rockefeller University
Federal Wide Assurance # 00004658
